# Expert Augmented Prediction of Circulatory and Respiratory Instability from High Resolution Vital Signs

**DOI:** 10.1038/s41746-026-02993-9

**Published:** 2026-07-13

**Authors:** Luhao Wang, Bin Gu, Yao Nie, Xingliang Jin, Chuanxi Chen, Fei Pei, Guangzhen Li, Ke Xiao, Xu Huang, Jin Liu, Hao Yan, Yu Xu, Sivasubramanium V. Bhavani, Jianfeng Wu, Xiangdong Guan

**Affiliations:** 1 Department of Critical Care Medicine, the First Affiliated Hospital, Sun Yat-sen University, Guangzhou, Guangdong, China; 2 Guangdong Clinical Research Center for Critical Care Medicine, Guangzhou, Guangdong, China; 3 Shenzhen Mindray Bio-Medical Electronics Co., Ltd., Shenzhen, Guangdong, China; 4 Institute of Precision Medicine, The First Affiliated Hospital, Sun Yat-sen University, Guangzhou, China; 5 School of Computer Science, Wuhan University, Wuhan, China; 6 British Heart Foundation Cardiovascular Epidemiology Unit, Department of Public Health and Primary Care, University of Cambridge, Cambridge, UK; 7 Division of Pulmonary, Allergy, Critical Care & Sleep Medicine, Emory University School of Medicine, Atlanta, GA USA

**Keywords:** Computational biology and bioinformatics, Diseases, Health care, Mathematics and computing, Medical research

## Abstract

Timely detection of circulatory and respiratory instability (CRI) is critical in intensive care units (ICUs), yet existing early warning systems often rely on single-parameter indices that underutilize continuous vital-sign data or on delayed, difficult-to-interpret multimodal clinical data. Leveraging routinely collected high-frequency vital-sign monitoring, we developed an interpretable, expert-augmented early warning system based on 1-second-resolution heart rate, blood pressure, respiratory rate, and oxygen saturation data. Machine‑learning models were trained on 627,958 h of continuous vital‑sign data from 1702 ICU patients at the First Affiliated Hospital of Sun Yat‑sen University and externally validated in the MIMIC‑III cohort. Models incorporating trend-based, reference, and statistical features derived from vital-sign trajectories achieved strong predictive performance (AUROC > 0.8) in both internal and external validation, outperforming conventional single-parameter indices and achieving performance comparable to models incorporating laboratory and demographic variables. Increasing temporal resolution improved predictive accuracy, with trend-based features contributing most strongly to model predictions. To improve clinical interpretability, tree-based models were transformed into physiologically meaningful decision rules and refined through expert-augmented learning, resulting in the Expert-Augmented Early Warning System (EAEWS). EAEWS generated accurate, low-frequency alerts with transparent explanations aligned with bedside monitoring, and may provide a scalable framework for real-time CRI detection in ICUs.

## Introduction

Critically ill patients in intensive care units (ICUs) require continuous monitoring, as their clinical status can deteriorate rapidly and timely intervention is often lifesaving^1^. However, the volume, velocity and complexity of data generated in modern ICUs can overwhelm clinicians, making it challenging to recognize early signs of physiological deterioration and intervene before critical events occur^2^. Reliance on human vigilance alone is therefore increasingly insufficient in such data-rich environments^3–5^. To address this, artificial intelligence (AI) has emerged as a promising tool to support earlier detection of patient instability and enhance clinical decision-making^6–10^.

Circulatory and respiratory failure are among the most common and life-threatening events in the ICU^11,12^, as they rapidly impair oxygen delivery and even short delays in intervention can substantially reduce survival^13,14^. Recent AI-driven early warning systems improve predictive capability beyond traditional intermittent vital-sign monitoring, by integrating multimodal patient data, including continuous vital signs, laboratory tests, and other clinical information, to capture both cross-sectional and temporal patterns that precede clinical deterioration^15–17^. These approaches have demonstrated promising accuracy in detecting impending circulatory and respiratory instability^18,19^. However, laboratory and other diagnostic tests are not always available in real time and may be delayed beyond the optimal intervention window, particularly in resource-limited ICU settings. Moreover, despite strong predictive performance, the large number and heterogeneity of input variables used in these models often render their decision processes opaque, making it difficult for clinicians to understand which physiological signals drive model predictions. This lack of interpretability remains a major barrier to the adoption of AI-based early warning systems in routine clinical practice^9^.

Vital sign monitoring remains the fundamental and universally implemented component in the ICU care, with modern bedside devices now capable of capturing physiological signals at high temporal resolution. These systems generate continuous streams of rich, high-frequency data, yet their potential to guide real-time patient management remains underexploited. Existing studies, including our own^20–22^, have demonstrated that dynamic patterns and temporal fluctuations in vital-sign trajectories contain clinically relevant information and can improve early detection of physiological instability^23–26^. Building on these insights, focusing exclusively on these widely accessible vital-sign data and integrating them with explainable AI approaches enables the development of early warning systems that not only provide accurate prediction of circulatory and respiratory instability, but also yield interpretable, physiologically meaningful insights.

Here, we built a clinical cohort of 1702 ICU patients at Sun Yat-sen University (SYSU) Hospital, comprising continuous 1-second-resolution vital-sign monitoring, comprehensive clinical records, and expert-annotated circulatory and respiratory instability (CRI) events across entire ICU stays. Leveraging these high-resolution signals, we developed machine-learning models for CRI prediction that incorporated both instantaneous and temporal features, and translated these models into a clinician-interpretable early warning system through expert-augmented learning. Through internal validation and external replication in the MIMIC-III, we show that models based solely on high-resolution vital signs achieve predictive performance comparable to those incorporating broader clinical information, with finer temporal resolution consistently improving accuracy. Our system retains predictive performance while providing transparent, physiologically grounded decision rules, enhancing its clinical relevance and interpretability.

## Results

### Overview of Study Design and Cohorts

**Fig. 1:**
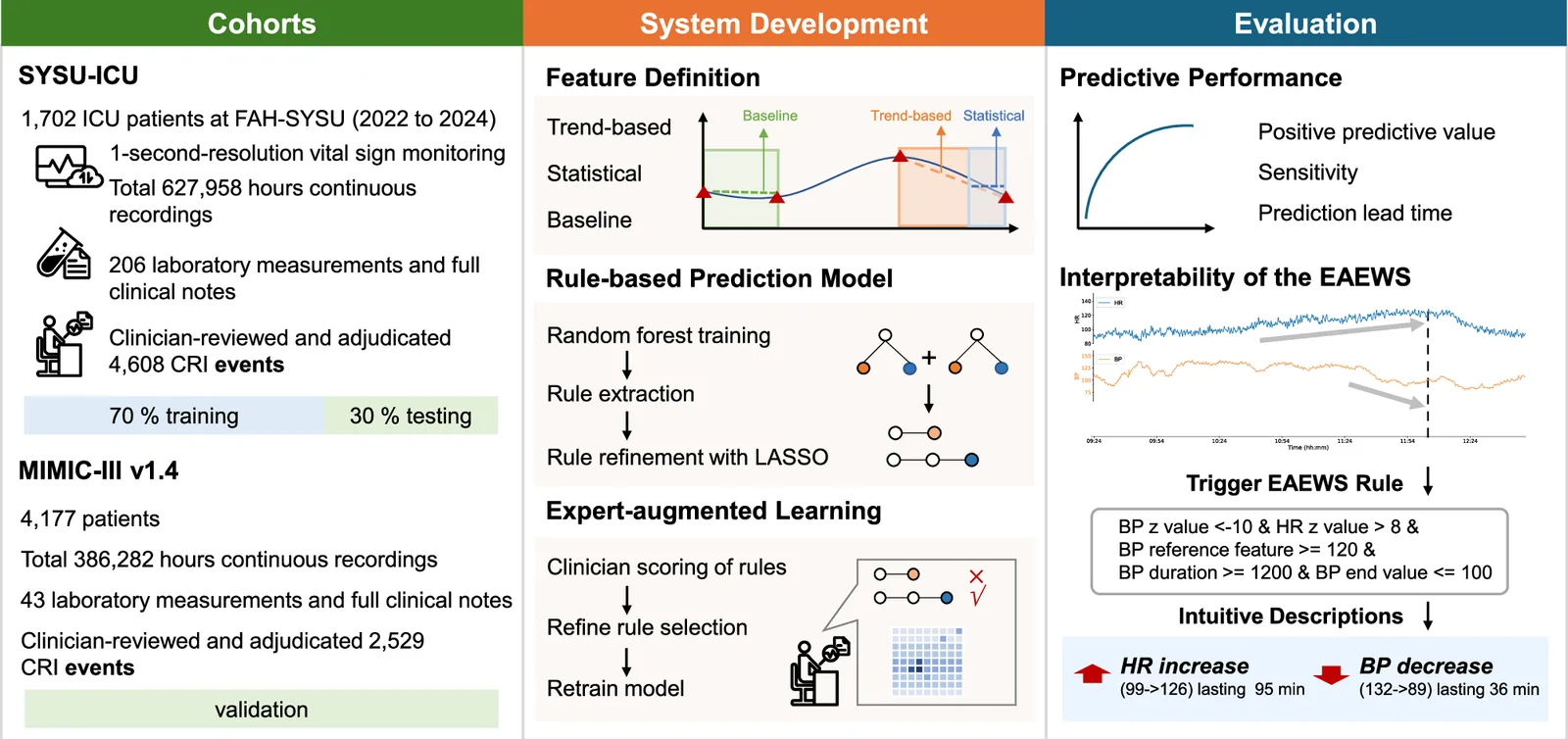
Study overview. **Cohorts:** The SYSU-ICU cohort comprised 1,702 ICU patients admitted to the First Affiliated Hospital of Sun Yat-sen University (FAH-SYSU) between 2022 and 2024, with continuous 1-second-resolution vital-sign monitoring (627,958 total hours), comprehensive clinical records including 206 laboratory measurements, and 4,608 clinician-reviewed and adjudicated circulatory and respiratory instability (CRI) events. The cohort was split into training (70%) and internal testing (30%) sets. External validation was performed using the MIMIC-III v1.4 cohort, which included 4,177 ICU patients with 386,282 h of continuous recordings, 43 laboratory measurements, full clinical notes, and 2,529 clinician-reviewed and adjudicated CRI events. **System Development:** The system was developed in three stages. **Feature definition**: baseline, trend-based, and statistical features were derived from high-resolution vital-sign time series. **Rule-based prediction model**: a Random Forest model was trained to predict CRI events, decision rules were extracted from the trained model, and refined using LASSO-based feature selection. **Expert-augmented learning**: clinicians reviewed and scored the extracted rules, which were then selectively retained and used to retrain the final EAEWS. **Evaluation:** The EAEWS was evaluated in terms of predictive performance (PPV, sensitivity, and prediction lead time) and its interpretability was demonstrated through transparent, rule-based decision logic.

**Fig. 2:**
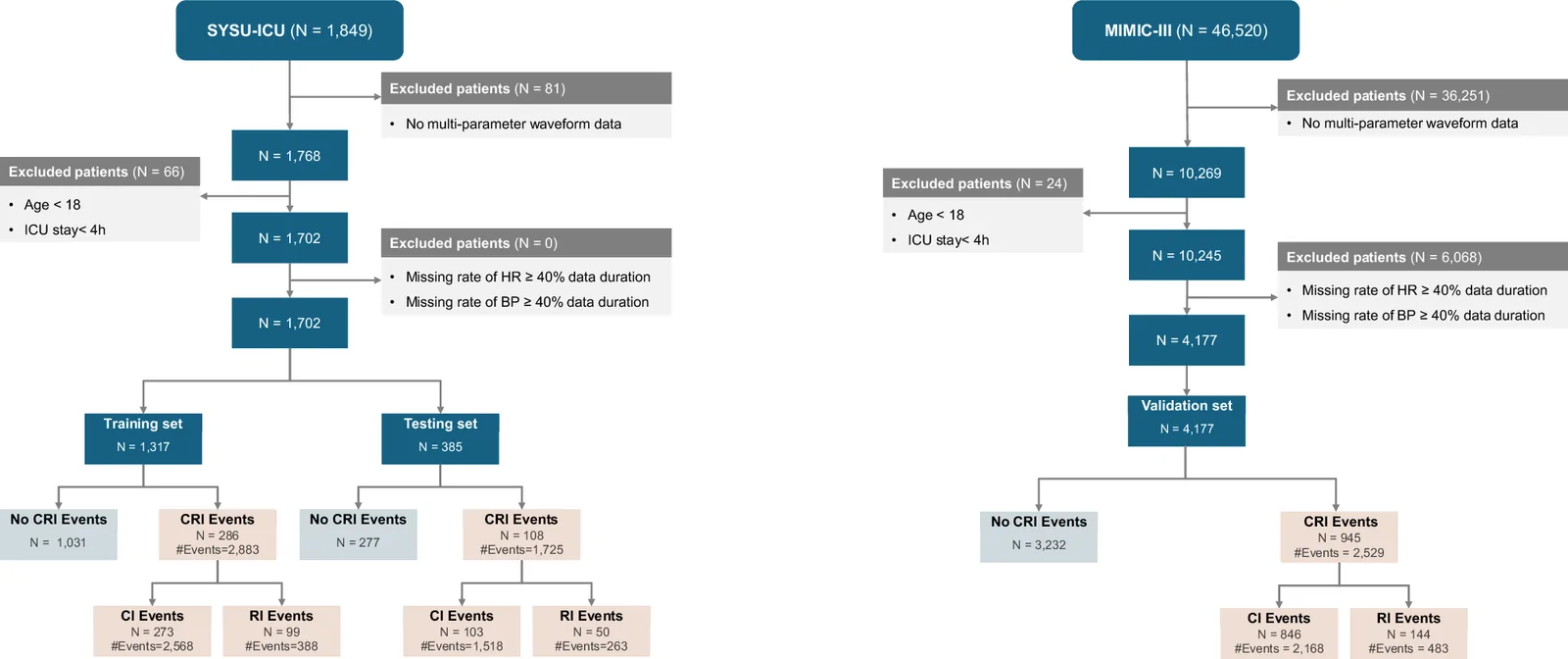
Study cohorts, quality control, and data partitioning. Flowchart showing patient inclusion, exclusion criteria, and dataset partitioning for the SYSU-ICU cohort (training and internal testing sets) and the MIMIC-III cohort (external validation).

We developed an interpretable dynamic early-warning system that continuously processes 1-second-resolution vital-signs (heart rate (HR), blood pressure (BP), respiratory rate (RR), and peripheral oxygen saturation (SpO_2_)) to predict forthcoming CRI events in the ICU (Fig. 1). Model development and internal validation (training and testing sets) were performed using data from the ICU of the First Affiliated Hospital of Sun Yat-sen University (SYSU-ICU), China (**Methods**), with external validation in the MIMIC-III v1.4 dataset^27^. The SYSU-ICU cohort included 1,702 patients, contributing 4,608 CRI events and 627,958 h of continuous physiological monitoring. In MIMIC-III, 4,177 patients with 2,529 CRI events and 386,282 monitored hours were retained for external validation. Baseline demographic and clinical characteristics of patients in the two cohorts were provided in Table 1, and detailed data quality control procedures and inclusion criteria are summarised in Fig. 2.

**Table 1:** The patients’ characteristic in SYSU-ICU and MIMIC-III

Characteristic	SYSU-ICU Training *n* = 1317	SYSU-ICU Testing *n* = 385	*P*-value	SYSU-ICU CRI *n* = 394	SYSU-ICU no CRI *n* = 1308	*P*-value	MIMIC-III CRI *n* = 945	MIMIC-III no CRI *n* = 3232	*P*-value
Male	821(62.34)	251(65.19)	0.31	254(64.47)	818(62.53)	0.49	579(61.27)	1899(58.75)	0.17
Age	61(49,71)	63(52,75)	0.004*	66(53,75)	60(48,70)	<0.001*	67(57,77)	65(54,75)	<0.001*
Preexisting condition- No. (%)									
Diabetes	271(20.57)	71(18.44)	0.36	87(22.08)	255(19.49)	0.26	318(33.65)	940(29.06)	0.007*
Hypertension	490(37.2)	139(36.1)	0.69	157(39.84)	472(36.08)	0.18	617(65.29)	1991(61.55)	0.04*
Coronary heart disease	203(15.41)	58(15.06)	0.87	72(18.27)	189(14.44)	0.06	407(43.06)	1296(40.06)	0.10
Chronic kidney failure	46(3.49)	18(4.67)	0.28	19(4.82)	45(3.44)	0.21	222(23.49)	446(13.78)	<0.001*
Tumor	598(45.4)	185(48.05)	0.36	183(46.44)	600(45.87)	0.84	160(16.93)	648(20.03)	0.03*
AKI	13(0.98)	2(0.51)	0.39	10(2.53)	5(0.38)	<0.001*	477(50.47)	838(25.90)	<0.001*
COPD	93(7.06)	25(6.49)	0.7	30(7.61)	88(6.72)	0.54	243(25.71)	646(19.97)	<0.001*
Heart Failure	30(2.27)	10(2.59)	0.72	11(2.79)	29(2.21)	0.51	362(38.31)	938(29.00)	<0.001*
Advanced Life Support-No. (%)									
CRRT	295(22.39)	115(29.87)	0.003*	182(46.19)	228(17.43)	<0.001*	199(21.06)	240(7.42)	<0.001*
Vasoactive drugs	892(67.72)	264(68.57)	0.76	354(89.84)	802(61.31)	<0.001*	679(71.85)	1061(32.80)	<0.001*
IABP	1(0.07)	1(0.25)	0.36	1(0.25)	1(0.07)	0.37	69(7.3)	147(4.54)	0.001*
Mechanical ventilation	1074(81.54)	315(81.81)	0.9	272(84.48)	1032(78.89)	<0.001*	632(66.88)	1254(38.76)	<0.001*

CRI Circulatory and respiratory instability event. AKI Acute Kidney Injury. COPD Chronic Obstructive Pulmonary Disease. CRRT Continue Renal Replacement Therapy. IABP Intra-Aortic Balloon Pump. *p < 0.05.

The development workflow consisted of four main components (see details in **Methods**): (1) **Event definition and labelling:** CRI events were defined using predefined physiological criteria and verified by clinical experts in both SYSU-ICU and MIMIC-III cohorts. In the SYSU-ICU cohort, the overall negative-to-positive ratio was 48:1 (training: 49:1; testing: 46:1). In the MIMIC-III cohort, the ratio was 55:1. (2) **Physiological feature derivation:** We constructed a rich set of derived features from high-resolution vital-sign time series, including statistical summaries, variability and trend measures to capture clinically relevant physiological patterns. (3) **Prediction model development:** A random forest model was trained to identify key predictive features and preliminary decision rules, which were subsequently refined using a LASSO model to enhance explainability. (4) **Expert-augmented learning:** Clinical experts reviewed, selected, and iteratively refined the machine-derived rules, which was integrated with machine learning to ensure physiological plausibility and interpretability, resulting in an expert-augmented CRI early-warning system.

### High-resolution Vital-sign Dynamics Enhance CRI Prediction

Using the 40 predefined physiological features derived from high-resolution vital-sign time series, we benchmarked multiple machine learning models for CRI prediction, including Random Forest (RF), XGBoost, GBM, and LSTM networks. In external validation using the MIMIC-III cohort, boosting-based methods achieved the highest AUROC (XGBoost: 0.852; GBM: 0.837), followed by RF (0.832) and LSTM (0.811) (Supplementary Fig. 4). Given its competitive performance and its suitability for downstream expert augmentation and rule-based interpretability, the RF model was selected for subsequent development of the CRI prediction framework using the SYSU-ICU training set. These models were subsequently compared with the final Expert-Augmented Early Warning System (EAEWS) using sensitivity, PPV, and F1 score at default decision threshold of 0.5 (Supplementary Fig. 13). (**Methods**).

**Fig. 3:**
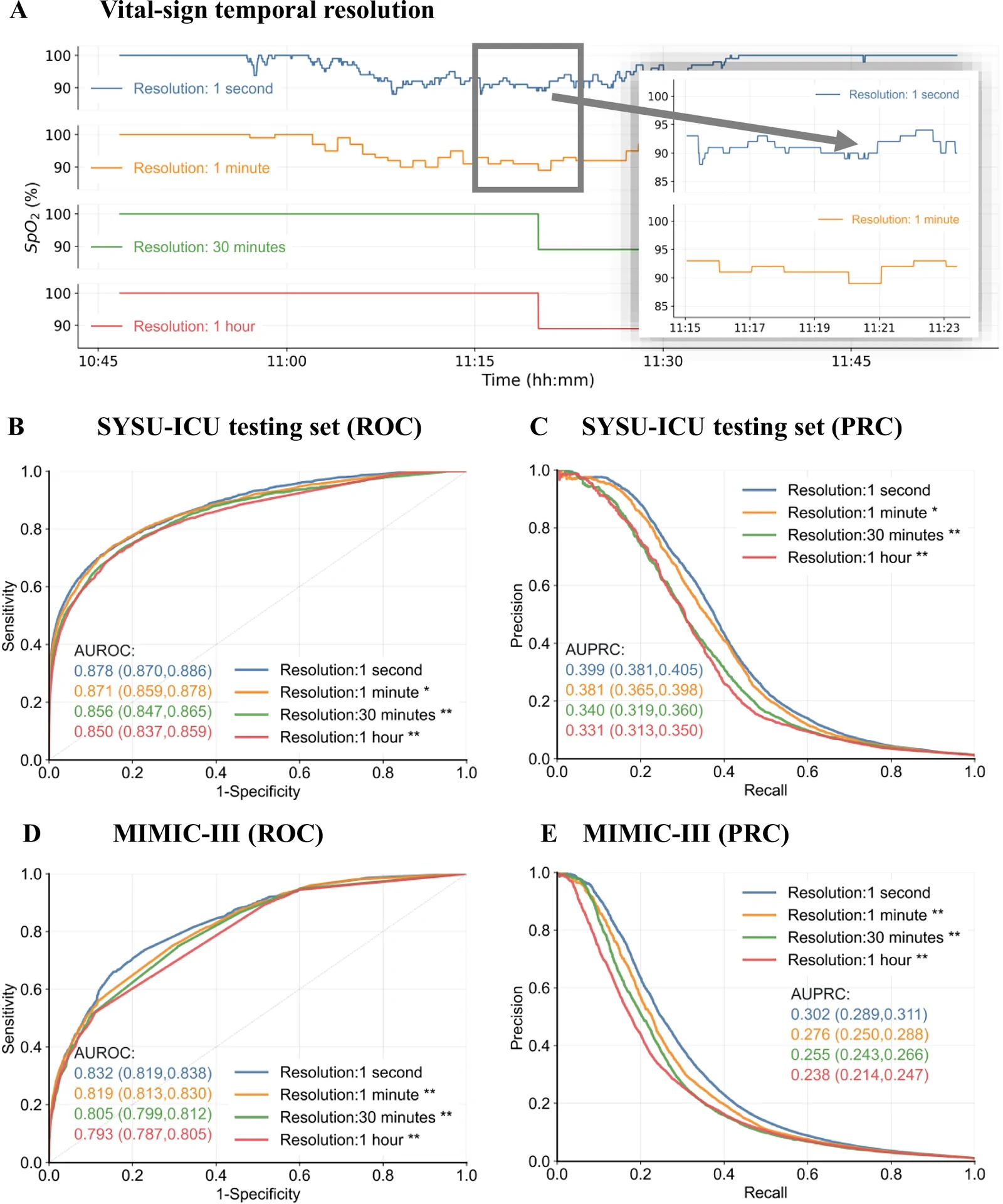
Impact of vital-sign temporal resolution on early warning random forest model performance. **A** Example SpO_2_ time series represented at four temporal resolutions (1 s, 1 min, 30 min and 1 h), illustrating the progressive loss of fine-grained physiological dynamics with temporal down sampling. **B** Receiver operating characteristic (ROC) curves for CRI prediction in the SYSU-ICU testing cohort using Random Forest models trained on vital-sign data at different temporal resolutions and the 40 predefined vital features (**Methods**). **C** Precision-recall curves (PRC) for the SYSU-ICU testing cohort corresponding to panel B. **D** ROC curves for CRI prediction in the external MIMIC-III cohort. **E** Precision-recall curves for the MIMIC-III cohort corresponding to **D**. * Denotes statistical significance compared to the resolution 1 s. *p < 0.05, **p < 0.01.

To assess the impact of vital-sign monitoring resolution on model performance, we compared models trained on data sampled at 1-s, 1-min, and 1-h intervals. Lower temporal resolution resulted in a marked decline in predictive accuracy (Fig. 3). For example, the AUROC decreased from 0.83 (95%CI: 0.82–0.84) to 0.82 (95%CI: 0.81–0.83, *p* < 0.01) when moving from 1-second to 1-minute data in the MIMIC-III external validation. SHAP analysis (Supplementary Fig. 9) further demonstrated that, relative to the 1‑minute model, the 1‑second model prioritized trend‑related features derived from rapidly fluctuating parameters (e.g. SpO_2_) among the top predictors, whereas mean‑value features exhibited comparatively lower importance.

**Fig. 4:**
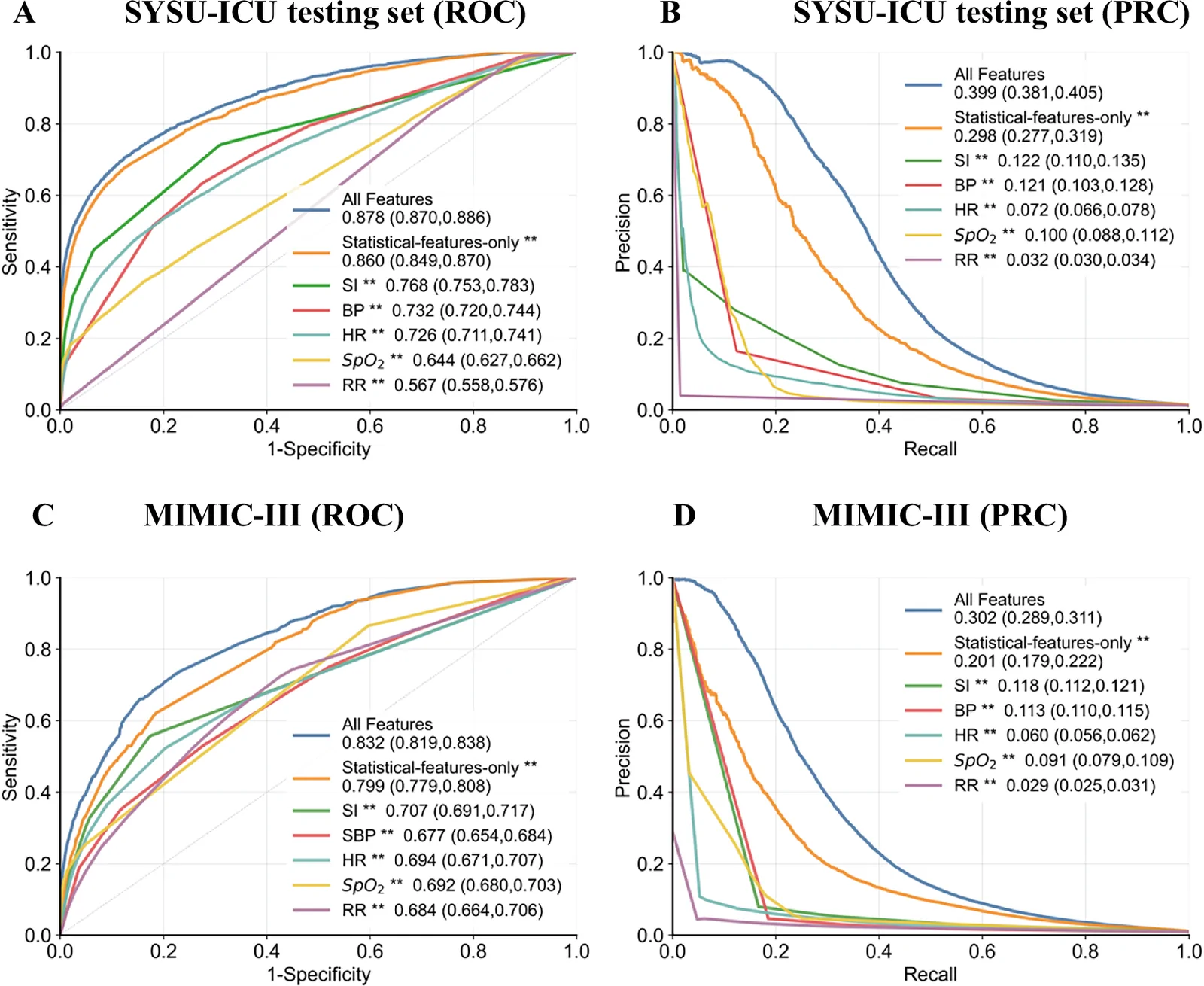
Comparative performance of vital-sign–based early warning models in SYSU-ICU and MIMIC-III. **A** Receiver operating characteristic (ROC) curves comparing the performance of different vital-sign-based models for CRI prediction in the SYSU-ICU testing set. **B** Corresponding precision-recall curves (PRC) for the SYSU-ICU testing set shown in **A**. **C** ROC curves for CRI prediction in the external MIMIC-III cohort. **D** Corresponding precision-recall curves for the MIMIC-III cohort shown in panel C. Models compared include an **All Features** model incorporating 40 predefined vital-sign features (**Methods**); a **Statistical-features-only** model using a subset of these features and excluding baseline and trend-based features; and models using individual physiological features alone, including **shock index** (**SI**), **peripheral oxygen saturation** (**SpO**_**2**_), **blood pressure** (**BP**), **heart rate** (**HR**), and **respiratory rate** (**RR**). SI was defined as the ratio of mean HR to mean BP. All models used the same 1-second–resolution vital-sign data, were trained on the SYSU-ICU training set using the Random Forest method, and were evaluated on the SYSU-ICU testing set, with external validation performed in the MIMIC-III cohort. * Denotes statistical significance compared to the All Features. *p < 0.05, ** *p* < 0.01.

Next, we compared the performance of the 1-second resolution random forest model with conventional single-parameter CRI risk predictors, including RR, HR, SpO_2_, BP, the Shock Index (SI), and model without trend‑related features^17,28^.The high-resolution vital-sign-based model consistently outperformed SI, BP and other single-parameter based models in terms of both AUROC and AUPRC in the SYSU-ICU internal validation as well as the MIMIC-III external validation (Fig. 4). In the MIMIC-III cohort, the vital sign model achieved an AUROC of 0.83 (95%CI 0.82–0.84), compared with 0.80 (95%CI 0.78–0.81) for vital sign model excluding trend‑related features, 0.71 (95%CI 0.69–0.72) for SI, and 0.68 (95%CI 0.65–0.68) for the BP model.

Using the same data split, we compared the 1-second resolution models (RF, GBM, LSTM and XGBoost) across baseline and extended settings, in which models were constructed using either the 40 predefined high-resolution vital-sign features alone or the same features combined with demographic and laboratory variables (Supplementary Table 14). In the MIMIC-III external validation cohort, compared with models using vital signs alone, the addition of demographic and laboratory variables increased the AUROC from 0.83 (95%CI 0.82-0.84) to 0.86 (95%CI 0.85-0.87) for RF and from 0.85 (95%CI 0.84-0.86) to 0.89 (95%CI 0.88-0.89) for XGBoost (Supplementary Figs. 4 and 5). In the RF framework, we further evaluated models that combined vital signs with either laboratory features or demographic features separately, demonstrating comparable model performance (Supplementary Fig. 6).

We also conducted sensitivity analyses using alternative prediction windows (1, 3, and 5 h) under the same RF framework. As the prediction window increased, AUROC, AUPRC, and PPV progressively declined, whereas sensitivity increased from the 1-h to the 3-h window and then decreased at 5-hour windows. In the MIMIC-III external validation cohort, AUROC decreased from 0.84 to 0.81, AUPRC from 0.31 to 0.28, and PPV from 0.73 to 0.54, while sensitivity increased slightly from 0.33 to 0.34 at 3-h windows before declining to 0.22 at 5-hour window (Supplementary Figs. 7 and 8).

### Expert-augmented Rule-based Modelling Improves CRI Prediction

To enable interpretability, we transformed the tree-based random forest model into a set of machine-learning IF-THEN rules for CRI prediction using a tree-traversal approach^29^, and subsequently refined these rules using LASSO to retain only the most predictive rules (**Methods**). Here, a rule is defined as a conjunction of feature-threshold conditions connected by AND operators (e.g., BP trend *z-value* < –1.0 AND HR trend *z-value* > 0.6) and represents a physiologically interpretable pattern with potential clinical relevance. A rule-based model is then constructed by combining multiple such rules using OR logic (Fig. 7), such that an alert is issued when all conditions of any single rule are satisfied.

From the 1-second-resolution vital-sign random forest model, 3,198 rules were initially extracted. LASSO pruning substantially reduced the rule set, while improving predictive performance (F1 score increased from 0.48 to 0.52 in the SYSU-ICU testing set), to 389 rules in the model^30^. Following expert-informed rule review and retraining, 180 rules were incorporated into the final Expert-Augmented Early Warning System (**EAEWS**), yielding a further improvement in performance (F1 score 0.56 in the SYSU-ICU testing set). The full EAEWS development workflow is described in the Methods. Among the 180 rules in the EAEWS, HR and BP were included in all rules, whereas RR and SpO_2_ were included in 27% and 38% of the rules, respectively.

**Fig. 5:**
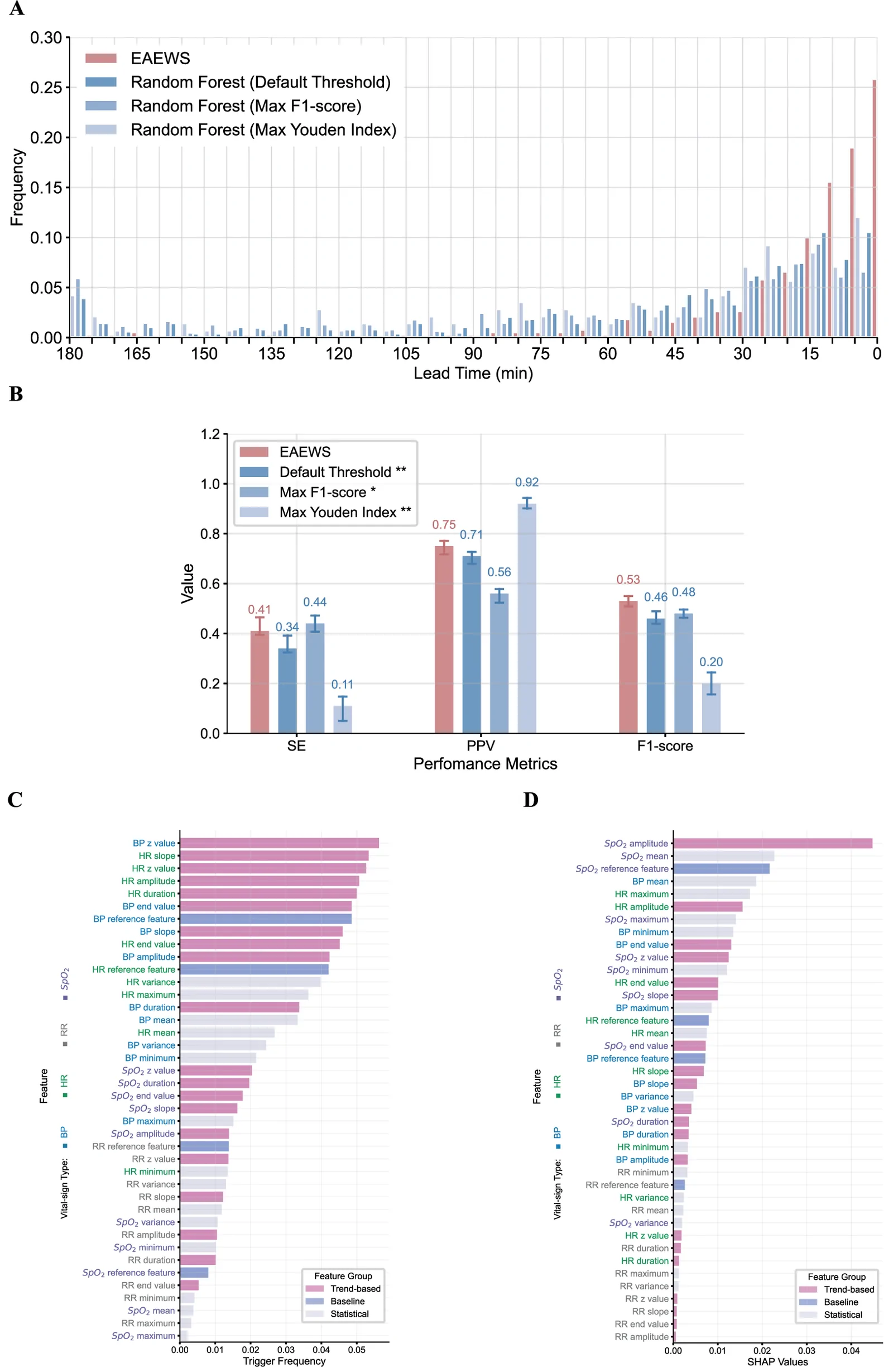
Comparative early-warning performance and feature contributions of the EAEWS and Random Forest models. **A** Distribution of early warning lead times for true-positive CRI events in external validation. For each true positive predicted CRI event in the MIMIC-III cohort, the earliest correct alert occurring within the 3-hour window preceding the annotated CRI onset was identified. The time difference between this alert and the CRI onset was defined as the early warning lead time. Lead times were grouped into 5-minute bins, and the frequency in each bin was calculated as the proportion of true positive CRI events whose earliest alert fell within that bin. Distributions are shown for alerts generated by the **EAEWS** and by a **Random Forest** model using three decision thresholds: the default probability threshold (0.5), the threshold maximizing the F1 score and the threshold maximizing the Youden Index. Random forest thresholds were determined using the SYSU‑ICU training set. **B** Model performance under different decision thresholds in external validation. Performance of EAEWS was compared with that of the Random Forest model under the three corresponding decision thresholds, evaluated on the MIMIC‑III cohort using sensitivity (SE), positive predictive value (PPV), and F1 score. Error bars represent 95% confidence intervals (CIs) for the differences. McNemar test was applied to compare EAEWS with other different decision thresholds. *p < 0.05, ** *p* < 0.01. **C** Global feature contributions in EAEWS ranked by trigger frequency in MIMIC-III. Feature contributions in EAEWS were quantified using trigger frequency, defined as the proportion of times a given feature appeared in satisfied rule conditions across all triggered alerts, relative to the total number of feature occurrences across all 40 features and all triggered alerts. Only features contained in rule conditions that were satisfied at the time of alert generation were counted. **D** Global feature attribution in the Random Forest model assessed using SHAP values. Feature contributions in the Random Forest model were evaluated using SHAP‑based feature attribution on the MIMIC-III cohort. All Random Forest models here used the same 1‑second–resolution vital‑sign data and the same set of 40 predefined vital‑sign features (**Methods**).

Using the SYSU-ICU testing set as a tuning set, we selected classification thresholds for converting the 1-second-resolution random forest model into a binary classifier, using either Youden Index or F1-score as optimization criteria. We compared the performance of EAEWS with that of these threshold-tuned random forest classifiers in the external MIMIC-III cohort. EAEWS demonstrated consistently overall better performance relative to all random forest classifiers, regardless of the threshold-selection strategy (Fig. 5 and Supplementary Fig. 10). In the MIMIC-III validation, EAEWS achieved an F1-score of 0.53, compared with 0.46 using the default probability threshold (0.5), 0.48 when tuned to maximize F1-score, and 0.20 when tuned to maximize the Youden Index. Feature contribution analyses suggested that trend-related features were prominent within EAEWS, as reflected by their high trigger frequency, whereas SHAP analyses of the random forest model highlighted these statistical features (Fig. 5 and Supplementary Fig. 10). While these interpretability metrics are not directly comparable, the results may provide an indication that EAEWS places greater emphasis on trend-related features.

We also evaluated the timeliness of CRI prediction for each model within the 3-hour risk window. Lead time was defined as the interval between the earliest warning issued by the model and the annotated onset of a CRI event. In the MIMIC-III, EAEWS correctly predicted **1045 of the 2529** CRI events, with alerts occurring most frequently immediately before event onset and a median lead time of 20 minutes [IQR: 5, 23]. For comparison, the random forest classifiers correctly identified **851,** **1109**, and **281** CRI events at the default probability threshold (0.5), the F1-score-optimized threshold, and the Youden Index-optimized threshold, respectively, yielding median lead times of 30 minutes [IQR:13, 74], 40 minutes [IQR:17, 89], and 29 minutes [IQR:14, 79] (Fig. 5 and Supplementary Fig. 10).

Consistency of EAEWS was evaluated, among correctly predicted events, as the proportion of minutes with positive alerts (one alert per minute) between the first correct alert issued within the 3 h prediction window and the event onset. In the main analysis, the model demonstrated stable temporal performance, with a median consistency of 0.750 [0.643, 0.900] in the SYSU-ICU testing cohort and 0.741 [0.591, 0.931] in the MIMIC-III external validation cohort. In addition, the distribution of correct prediction proportion over time showed an increasing trend as the event approached in both cohorts (Supplementary Fig. 14).

### Complementary Circulatory and Respiratory Contributions to CRI Prediction

**Fig. 6:**
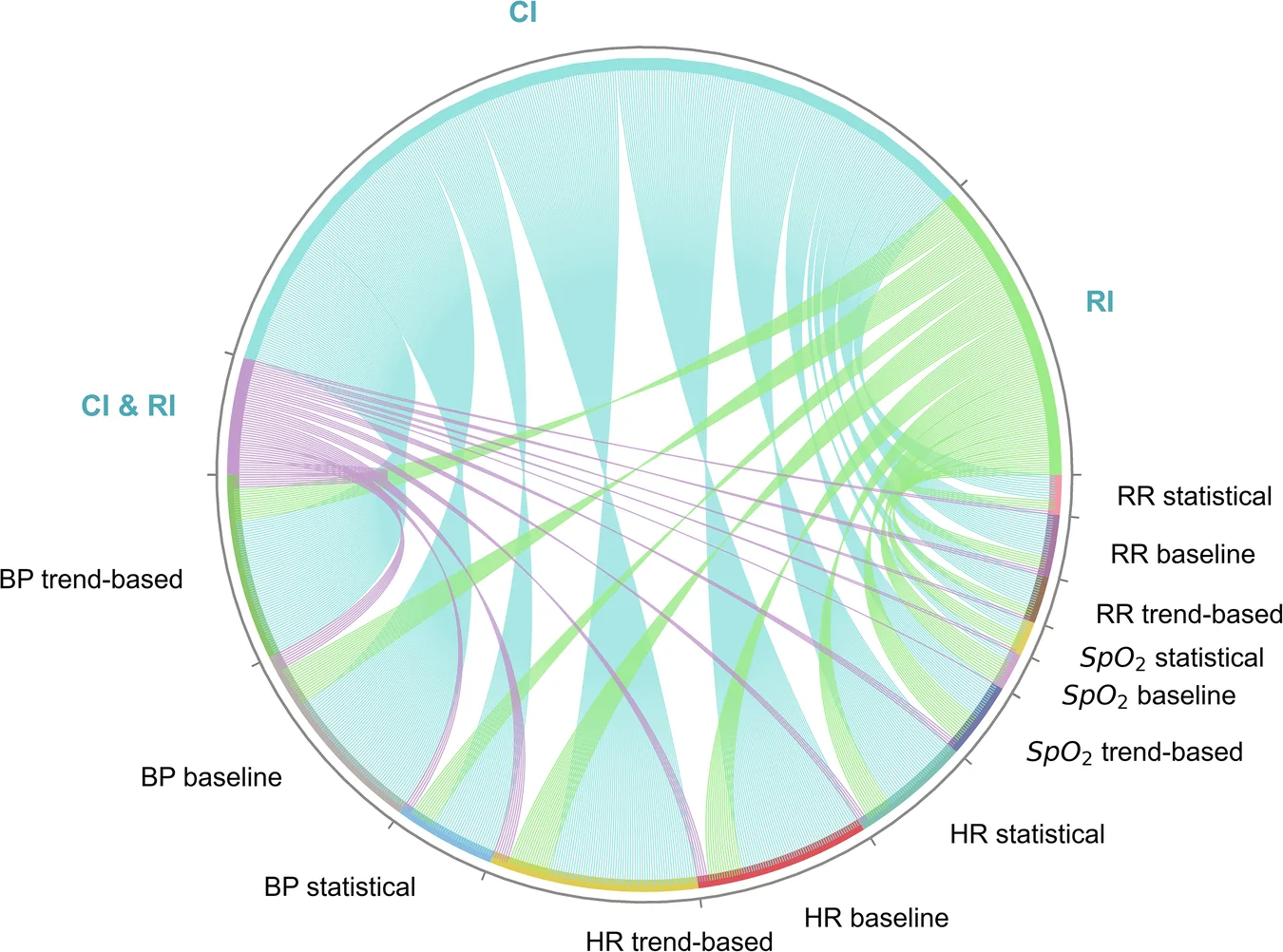
Circulatory- and respiratory-derived feature categories contributing to correct prediction of circulatory and respiratory instability in MIMIC-III. The chord diagram summarizes the feature-threshold conditions that contributed to correct rule activation for circulatory and respiratory instability (CRI) predictions in the MIMIC-III cohort. The upper semicircle represents correctly predicted event types, stratified as circulatory instability only (CI), respiratory instability only (RI), and combined circulatory and respiratory instability (CI&RI). The lower semicircle shows aggregated feature categories corresponding to the feature-threshold conditions of the decision rules involved in these correct predictions. The original 40 model features were grouped into 12 combined feature categories based on vital-sign modality (HR, BP, RR and SpO_2_) and feature group (baseline, trend-based, and statistical). Chords connect event types to feature categories that contributed to correct rule activation. For each event type, the width of each feature-category segment represents the proportion of features belonging to that category among all feature categories appearing in satisfied rule conditions for correctly triggered alerts of that event type.

HR and BP primarily capture circulatory dynamics, whereas RR and SpO_2_ reflect respiratory status; however, all four vital signs have been shown to be informative for predicting both CI and RI events in ICU patients^31–34^. We examined the feature-threshold conditions that constitute decision rules in EAEWS correctly predicting CRI events (referred to as triggering conditions), stratified into three categories: CI-only events (*N* = 732), RI-only events (*N* = 221), and combined CI&RI events (*N* = 92). Each rule comprises multiple logical conditions (e.g., BP z value < -10 & HR z value > 8 & BP end value < 100 & RR z value >= 5 & SpO_2_ z value < -3), which we grouped by vital sign–feature type pairs (12 categories in total). We quantified the proportion of conditions contributing to correct rule activation across event types (Fig. 6 and Supplementary Fig. 11).

For CI-only events, conditions derived from HR and BP accounted for the majority of triggering conditions (82.3%), as expected, while RR- and SpO_2_-derived conditions also contributed substantially (17.7%). For RI-only events, although HR- and BP-derived conditions remained dominant (69.1%), RR- and SpO_2_-derived conditions made a meaningful contribution (30.9%). Notably, all 180 rules contained at least one CI-related condition (HR and/or BP), whereas only a subset of rules (68) additionally incorporated RI-related conditions (RR and/or SpO_2_). Among RI events correctly predicted in the MIMIC-III cohort, 16.74% were first detected by rules that included RR- and/or SpO_2_-derived conditions, with no earlier activation of rules containing only CI-related conditions; a similar pattern was observed in the SYSU testing set cohort (11.62%).

Although circulatory features formed the core of the prediction model, respiratory (RI) features still contributed meaningful complementary information. When using CI features alone, model performance was substantially reduced (e.g., F1 score 0.38 vs 0.56 in SYSU testing set; 0.33 vs 0.53 in MIMIC-III, Supplementary Fig. 16), whereas combining CI and RI features led to notable improvements across both cohorts.

### Interpretable Rule-based Alerts Compatible with Bedside Vital-sign Monitoring

**Fig. 7:**
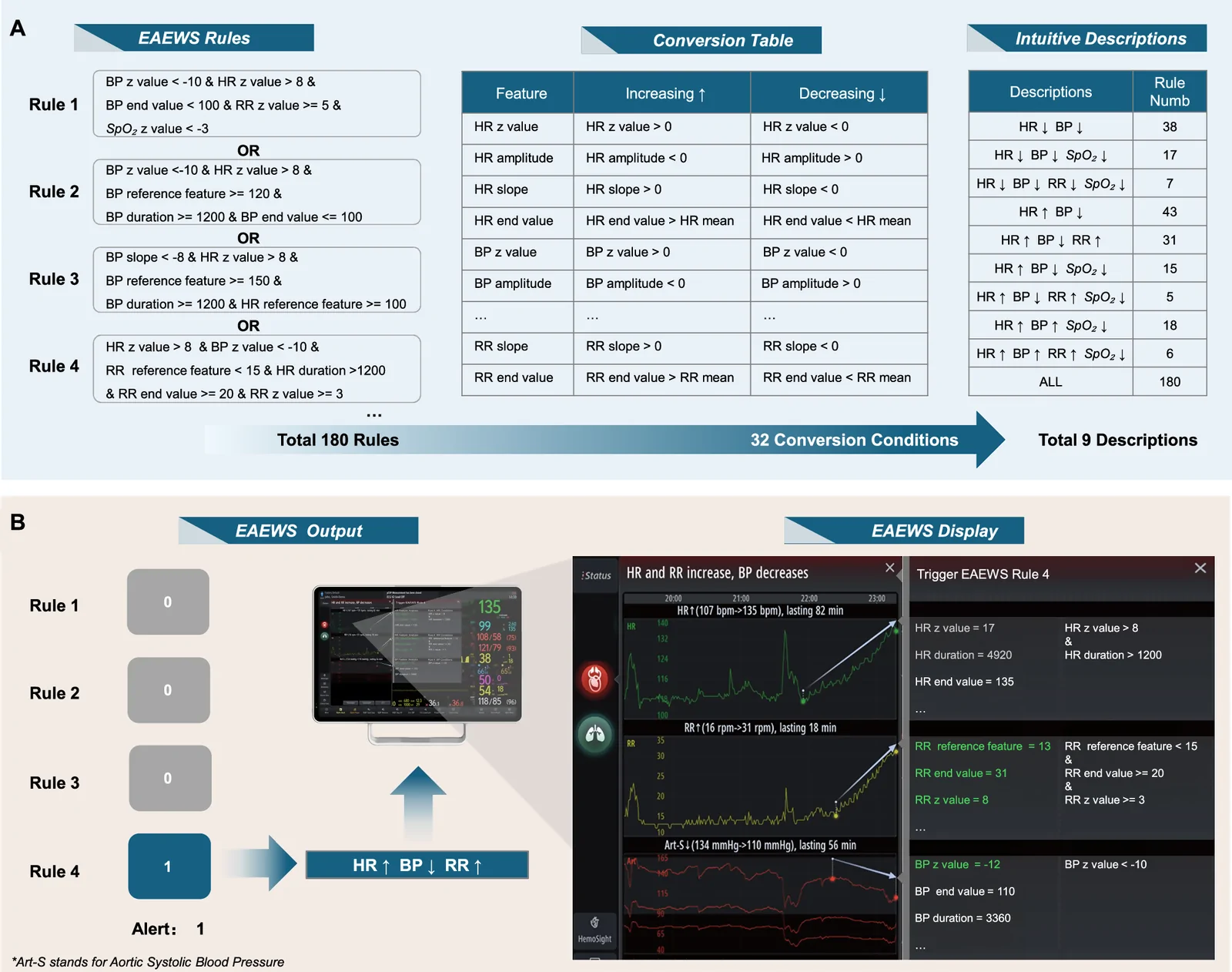
Interpretability and bedside integration of the Expert-Augmented Early Warning System (EAEWS). The EAEWS is designed to provide transparent, clinically intuitive alerts that can be readily integrated into existing ICU vital-sign monitoring workflows. For each alert, the system outputs not only a warning signal but also an interpretable explanation directly linked to changes in routinely monitored vital signs. **A** Translation of machine-learned EAEWS decision rules into intuitive clinical descriptions. The original set of 180 decision rules, expressed as feature-threshold-based conditions on temporal dynamics, captures combinations of trend changes across four routinely monitored vital signs. Because these trend patterns are not directly observable on conventional bedside monitors, rules with similar combinations of trend changes were grouped and mapped to directional descriptors (increase or decrease) for each vital sign. This procedure condensed the original rule set into nine clinically intuitive descriptions that summarize underlying trend behavior and can be displayed alongside the absolute vital-sign values shown on bedside monitors. **B** Example of the clinician-facing EAEWS display integrated with real-time vital-sign monitoring. Alerts are presented alongside standard waveform and numeric displays, with concise descriptions of the underlying trend-change patterns represented by directional arrows overlaid on the vital-sign traces; rule-level information underlying each alert can be accessed in an accompanying panel, with vital-sign values meeting the feature-threshold conditions of the triggered rule(s) highlighted in green.

Rules in EAEWS comprised a small number of feature-threshold conditional statements, with a mean of 8.9 conditions per rule (95% range: 6–11); each alert triggered few rules, with a mean of 1.15 rules (95% range: 1–2) in the SYSU-ICU testing set and 1.09 rules (95% range: 1–2) in MIMIC-III. This compact rule structure enables EAEWS to integrate naturally with existing bedside vital-sign monitors and supports real-time interpretability. The 180 EAEWS rules were grouped into nine predefined vital-sign trend patterns (for example, HR increase with BP decrease), defined by combinations of trend changes across the four vital signs that are not directly visible in raw waveform displays (Fig. 7). For each alert, activated rules were mapped to one of these nine trend-pattern groups and displayed alongside the corresponding vital-sign waveforms (Fig. 7), providing an intuitive summary of the physiological processes underlying the alert, while preserving access to detailed rule logic.

## Discussion

We demonstrated that machine learning models trained on multidimensional continuous vital signs at a second-by-second resolution can predict circulatory and respiratory instability events with AUROC values exceeding 0.8 in both internal and external validation. These models substantially outperformed conventional single-parameter indices and achieved performance comparable to models that incorporated additional laboratory and demographic variables. Although multimodal clinical information may provide incremental gains, our findings suggest that vital-sign dynamics alone, that captured at higher temporal resolution, can provide informative signals for impending CRI prediction, particularly in settings where laboratory and diagnostic data are not available in real time or may be delayed beyond the optimal intervention window^35^. However, the magnitude and clinical relevance of these improvements remain to be established, and further prospective studies are needed to evaluate their utility in real-world settings.

We then developed the Expert-augmented Early Warning System (EAEWS) by transforming the machine-learning models into a rule-based prediction framework and refining these rules through expert-augmented learning, yielding a decision process that is transparent and clinically interpretable. We showed that the EAEWS improved predictive performance relative to the original machine-learning models, while offering clear, physiologically grounded explanations for each alert. By integrating expert insights with data-driven modelling, the system mitigates the typical trade-off between predictive accuracy and interpretability. This human-in-the-loop approach aligns with a growing consensus that clinically effective AI systems should combine the complementary strengths of machine learning and domain expertise to achieve both robust performance and trustworthy decision support^36^.

Our analyses indicated that trend-based features derived from HR and BP contributed more strongly to system decisions than absolute vital-sign values. This pattern may reflect both the central role of hemodynamic change as a shared upstream process preceding circulatory and respiratory failure in critically ill patients^37,38^ and the ability of trend dynamics to capture evolving physiological changes that are difficult to discern from visual inspection of real-time bedside waveforms^39^. Consistent with this interpretation, isolated respiratory instability appeared to be relatively uncommon before CRI events in our data. However, our results also demonstrated that respiratory-derived conditions from RR and SpO_2_ provide complementary, non-redundant information, particularly for a subset of CRI events for which early detection based on circulatory conditions alone is less effective. Together, these observations support joint modelling of circulatory and respiratory features, highlighting the importance of trend-based dynamics for early detection of evolving circulatory and respiratory instability in the ICU.

Our study has several limitations. First, as with other retrospective analyses, event definitions were constrained by available data. Clinical interventions (e.g., vasopressors or oxygen therapy) may alter MAP and SpO_2_ and thus influence labeling. Under our conservative criteria, only threshold-based events were labeled; therefore, cases in which early interventions prevented deterioration were classified as non-events. This may lead to under-ascertainment, reflecting the limitation that counterfactual outcomes cannot be directly observed. Second, although EAEWS achieved a high positive predictive value (PPV > 0.7), its sensitivity was relatively modest (SE < 0.5). As a result, alerts issued by EAEWS were highly precise and infrequent, with low alert rates in both the MIMIC-III (0.049 alerts per patient-hour) and SYSU-ICU testing set (0.055 alerts per patient-hour). This may reflect the conservative nature of expert-derived rules used during model development. As an initial attempt to integrate expert knowledge with data‑driven modeling, this conservative strategy may help improve clinical acceptability and trust. Third, although the model was trained using a 3-h prediction window to estimate the risk of CRI within the subsequent 3 h, observed alerts were more frequently concentrated closer to event onset, with a median lead time of approximately 20 min. This reflects the inherent challenge of long-horizon prediction in physiological time-series data, where early signals are often weaker and less specific, whereas signals closer to the event are more pronounced and detectable. Accordingly, the model should be interpreted as operating within a predefined 3-h risk detection window, rather than as a fixed lead-time predictor. While the effective lead time is relatively short, such alerts may still be clinically actionable in the ICU setting, enabling focused assessment and timely intervention during acute deterioration. Notably, prior work (the Hypotension Prediction Index) has also reported clinically useful alerts within short lead-time windows (5–15 min)^23^, supporting the potential utility of this temporal range^25,40^. Fourth, comparisons between vital-sign-only models and models incorporating laboratory tests were restricted to laboratory variables available in the study datasets. Consequently, the observed performance differences may depend on the specific laboratory tests included and may not reflect the full potential of multimodal models incorporating a broader range of laboratory data.

Future research should focus on two key directions to advance CRI prediction: first, incorporating additional hemodynamic and respiratory variables, integrating broader multimodal data sources (including an expanded panel of laboratory tests to fully unlock the potential of multimodal modeling), and exploring more advanced explainable models to enhance predictive performance and robustness; second, conducting further prospective evaluations to quantify the magnitude and clinical impact of the achieved lead times, while assessing how prediction horizons, alert timing, and clinical decision-making interact in real-world clinical settings.

## Methods

### Data and Quality Control

This study utilized two datasets: a cohort collected at the First Affiliated Hospital of Sun Yat-sen University and the MIMIC-III database^27^. Ethical approval for the cohort collected at the First Affiliated Hospital of Sun Yat-sen University was obtained from the IEC for Clinical Research and Animal Trials at the First Affiliated Hospital of Sun Yat-sen University (Approval No. [2023] 363). Access to the MIMIC-III database was granted after the author completed the required Collaborative Institutional Training Initiative (CITI) training (Completion ID: 53168708). This study was conducted in accordance with the Declaration of Helsinki.

In the SYSU-ICU dataset, we included 1,849 patients admitted to the intensive care unit of the First Affiliated Hospital of Sun Yat-sen University between December 1, 2022, and September 30, 2024. Given the retrospective design of this data analysis study, informed consent was waived by the ethics committee.

All patients underwent continuous monitoring with Mindray BeneVision N series, which records vital signs at second-level resolution, including heart rate (HR), blood pressure (BP), respiratory rate (RR), and peripheral oxygen saturation (SpO_2_). We excluded patients with ICU stays shorter than 4 h, those younger than 18 years or had <60% coverage for HR and BP data. After applying these criteria, 1,702 patients remained, with a total of 627,958 monitoring hours data available for analysis. During continuous monitoring, signal loss may occur due to medical procedures or clinical interventions interference. The overall missing rates were 23.2%, 30.4%, 23.1%, and 25.5% for HR, BP, RR, and SpO_2_ respectively in the SYSU-ICU study. For the four vital signs, missing data with gaps shorter than 3 h were imputed using forward mean imputation, with the mean calculated from the preceding 1 h of continuous data. Signal preprocessing followed our previously published methodology and the device operator manual^41,42^. Briefly, signal quality was assessed using a signal quality index (SQI), and low-quality or artifact-contaminated signals were excluded. The SpO_2_ signals were additionally smoothed using a 6-second time window, with a 2-second sliding step for each update.

Circulatory and respiratory instability (CRI) events in the SYSU-ICU dataset were defined based on clinically meaningful physiological thresholds and corresponding interventions, as established previously^28,43^. An event was labeled as CRI if all of the following three criteria were met: (1) The patient exhibited hypotension, defined as a mean arterial pressure (MAP) < 65 mmHg for more than one minute, or hypoxemia, defined as an SpO_2_ < 90% for more than one minute. (2) When events met the first condition, expert review was conducted to determine whether an intervention would have been clinically warranted. In events with a corresponding clinical intervention documented in the electronic medical record near the time of hypotension or hypoxemia, experts judged whether the intervention was related to the event (specific intervention types are listed in Table 2). For events without a documented intervention, experts assessed whether an intervention would have been appropriate during the event window and, if so, identified the intervention that should have been taken. Events with recorded interventions that did not meet the first criterion were not classified as CRI events. (3) Two independent clinical experts were invited to assess the event using the patient’s electronic medical records, including vital signs and other relevant clinical information. If cases of disagreement, a third expert was invited to adjudicate the event. The final event label was determined by consensus. Negative labels were defined from time segments remaining after exclusion of predefined time windows. For patients with CRI events, these exclusion windows included the 3 h preceding and 1 h following each event, as well as the first 3 h after ICU admission. For patients without CRI events, only the first 3 h after ICU admission were excluded. All remaining time segments were labeled as negative. (Supplementary Fig. 3). For patients with positive events, feature data were extracted from the period preceding the labeled event; for patients without positive events, all feature data were included.

**Table 2:** Circulatory and Respiratory Related Interventions

Pharmacological Interventions	Definition
**Vasoactive agent Treatment**	Treated with inotropes and vasopressors: Dobutamine, Dopamine, Epinephrine and Norepinephrine, Vasopressin
**Fluid Therapy**	Crystalloids and colloids infusion (2400 ml in 8 h, 3000 ml in 12 h, 700 ml in 1 h, 1500 ml total in 4 h, 500 ml twice in 4 h)
**Emergency Blood Transfusion**	Platelets, red blood cells (packed red cells), or plasma (4 U over the course of 24 h, 2 U in two hours followed by fluid therapy within 12 h, 2 U not followed by fluid therapy within the following 24 h) transfusion
**Circulation Support**	ECMO, IABP
**Respiratory Support**	Conducted invasive mechanical ventilation (transition from non-invasive to invasive), or non-invasive respiratory support (transition from no intervention to non-invasive, excluding transition from invasive to non-invasive)

*ECMO* Extracorporeal Membrane Oxygenation, *IABP* Intra-Aortic Balloon Pump.

In the SYSU-ICU dataset, a total of 4608 CRI events were identified among the 1702 patients. They included 4086 (88.7%) circulatory events and 651 (14.1%) respiratory events with 129 (2.8%) overlapping events involving both circulatory and respiratory instability. Among these, 1034 (22.4%) circulatory events and 181 (3.9%) respiratory events were identified by experts despite the absence of a documented intervention (Supplementary Table 9). The SYSU-ICU cohort were used for model training and internal validation (70%/30% split by admission date; Fig. 2), owing to the high completeness of its EHR data and the resulting higher labeling quality of CRI events (Supplementary Table 1-8). All data splits (training, testing and validation) were performed at the patient level to prevent intra-patient data leakage, ensuring that no samples from the same patient appeared in more than one set.

In the MIMIC-III dataset. We used version 1.4 of the MIMIC-III dataset to externally validate the CRI risk prediction models as it includes a waveform database with about 22,000 recordings from 10,000 patients, enabling extraction of high-resolution vital-sign data. This dataset includes 46,520 patients admitted to the critical care units of Beth Israel Deaconess Medical Center between 2001 and 2012. After subsetting to the patients with waveform data and applying the same quality-control criteria as in the SYSU-ICU study, 4177 patients with a total of 386,282 monitoring hours were retained for analysis (Fig. 2). The missing rates for HR, BP, RR, and SpO_2_ data in the MIMIC-III are 2.4%, 15.4%, 47.2%, and 49.9% respectively. The same imputation approach used in the SYSU-ICU study was applied to the four vital signs in MIMIC-III. CRI events were defined using the same clinical and expert assessment combined approach as in the SYSU-ICU dataset, yielding a total of 2529 events: 2168 (85.7%) circulatory events, 483 (19.1%) respiratory events, and 122 (4.8%) overlapping events. Among these, 694 (27.4%) circulatory events and 208 (8.2%) respiratory events were identified by experts despite the absence of a documented intervention (Supplementary Table 9). Clinical characteristics of the two cohorts are presented in Table 1.

### Development of Machine Learning Models for CRI Prediction Using High Resolution Vital Signs

We first developed a random forest model using high-resolution vital-sign data. Vital-sign data recorded after the first 3 h following ICU admission were used for model inputs, which include HR, BP, RR and SpO_2_. We used systolic blood pressure (SBP) to represent BP because of its greater sensitivity in detecting physiological deterioration.

Feature extraction focused on time-series signal characteristics with both clinical interpretability and physiological relevance. The feature definition process combined insights from time-series signal processing methodologies^44–46^, clinical expertise in patient assessment, and prior clinical early warning studies^28,47^. We derived three categories of features for each vital sign: (1) trend-based features (slope, z value, amplitude, end value and duration); (2) baseline/reference features, and (3) statistical features (mean, variance, maximum, minimum) (Supplementary Table 12). In total, we retained 40 features, all updated every minute (Supplementary Table 13).

We applied two windowing strategies to capture temporal dynamics: a fixed 15-minute window and a trend-adaptive window. First, statistical features were calculated within the fixed preceding 15-minute window. Next, the Mann-Kendall (MK) test^48^ was used to detect inflection points and define trend-adaptive windows. In the MK framework, the normalized z-value characterizes temporal trends, where the sign indicates the direction of change (positive for increasing trends and negative for decreasing trends), and the absolute value reflects the strength of the trend. Z-value statistics were calculated for the forward and backward sequences of each data segment to identify the intersection point, i.e. the inflection point. The detailed formula for z-value calculation is provided below.

For time series$\,{x}_{1}$,$\,{x}_{2}$,…${x}_{n}$, the S statistic is first calculated as follows:

$$S={\sum }_{i=1}^{n-1}{\sum }_{j=i+1}^{n}{{sgn}}({x}_{j}-{x}_{i})$$

where $\mathrm{sgn}\left(x\right)$ is the sign function, defined as:

$${sgn}\left(x\right)=\left\{\begin{array}{c}1\qquad{if}\,x > 0\\ 0\qquad{if}\,x=0\\ -1\qquad{if}\,x < 0\end{array}\right.$$

Then we calculate S variance ${Var}\left(S\right)$:

$${Var}\left(S\right)=\frac{1}{18}[n\left(n-1\right)\left(2n+5\right)-{\sum }_{k=1}^{p}{t}_{k}({t}_{k}-1)(2{t}_{k}+5)]$$

where, ${t}_{k}$ is the number of tied values in the k-th group, and p is the number of tied groups.

The normalized z-value of the trend is calculated as follows:

$$z=\left\{\begin{array}{ll}\frac{S-1}{\sqrt{{Var}(S)}}\qquad{if}\,S > 0\\ 0\qquad{if}\,S=0\\ \frac{S+1}{\sqrt{{Var}(S)}}\qquad{if}\,S < 0\end{array}\right.$$

We determine whether the trend-adaptive window constitutes a stable segment by using the z-value. The baseline feature was defined as the mode value of the all-stable segments over the previous 12 hours (Supplementary Table 11). The trend features were calculated in the most recent trend-adaptive windows detected (Supplementary Table 10). An illustration of the calculation for these features is shown in Supplementary Fig. 2.

We then trained random forest models using SYSU-ICU training set to predict the occurrence of CRI events within the subsequent 3-hour period at any given time point. Hyperparameters, including tree depth, number of child nodes, and number of trees, were optimized using grid search based on the SYSU-ICU testing set. The SHAP method was used to analyze the contribution of each feature to the random forest model.

The model takes 40 input features and generates a binary prediction (0, no CRI risk; 1 high CRI risk) at 1-min intervals. To enhance interpretability, we converted the trained tree-based model into a set of machine-learned IF-THEN rules, which were further refined using LASSO to retain the most predictive rules^29^. LASSO regression with L1 regularization was applied for optimization, with a hyperparameter α = 0.0014. Rules were processed in batches to train the LASSO using the SYSU-ICU training set.

### Development of the Expert-Augmented Early Warning System

To develop an interpretable early warning system for circulatory and respiratory instability (CRI) in the ICU, we designed a three-stage pipeline that combines the predictive power of machine learning with clinical expertise for interpretability: (1) extraction of physiologically meaningful features from high-resolution vital sign data; (2) machine learning-based model training and rule generation; and (3) integration of clinical expertise through expert-augmented rule refining and retraining.

Following LASSO rule selection from the random forest model, the construction of the expert-augmented early warning system (EAEWS) involved two additional steps: expert-informed rule selection and retraining. When evaluating the EAEWS system, we treated continuously triggered rules as a single alert event. The model was evaluated based on the Sensitivity (SE) and Positive Predictive Value (PPV) of alert-event correlations. The same evaluation method was also adopted for retraining.

Our model uses 40 features (comprising 10 derived metrics for each of the 4 vital signs). In the first step, these features were used to train the RF model, generating an initial pool of 3198 rules; these were subsequently filtered via LASSO regression, resulting in 389 rules (see details from the above section).

To integrate clinical knowledge with algorithmic performance, we applied a dual-evaluation approach^30^. Each machine learning-derived rule from the model training stage was first evaluated quantitatively using mean squared error (MSE) against labeled outcomes in the SYSU-ICU training set, and ranked by predictive accuracy. In parallel, three clinical experts were invited independently to review each rule, assigning a plausibility score from 1 to 3 (1 = poor alignment with clinical knowledge, 2 = required refinement, and 3 = strong consistency with clinical practice). The final expert score was defined as the average of the three ratings for each rule. Rules with both an expert score of 1 and an accuracy ranking in the lowest tertile were excluded. After rule selection, the 389 rules were refined to 180 final decision rules.

Then we conducted rule retraining. We refined the 180 decision rules by incorporating expert knowledge again. For each of the initial 180 rules, we presented the characteristics of all alerted cases in SYSU-ICU training set, the 40 feature values at the trigger point, and the raw vital sign data from the 3 hours preceding the trigger to a panel of 15 clinical experts. Using a validation matrix (180 rules * 40 features), experts evaluated each rule and categorized into two types: modifications to existing rules and additions of new rule conditions. In cases of conflicting expert opinions, the majority vote prevailed. Based on this feedback, the feature composition of each rule was reconstructed. Importantly, expert refinement was conducted exclusively on the SYSU-ICU training set. Experts were blinded to validation performance, and no information from the SYSU-ICU testing set or MIMIC-III dataset was used during rule optimization. After rule reconstruction, these rules were applied to the dataset, and grid search was employed to re-optimize the decision thresholds for the updated feature combination.

Finally, we conducted rule translation. Our warning system displays the corresponding decision rules when an alert is issued. While the system presents the logic behind each trigger, raw computational rules are often difficult to interpret. To address this, we translated these raw rules based on trend feature conditions into 9 clinically intuitive descriptions (e.g., ‘HR increase’, ‘BP decrease’).

Through the following process, the 180 decision rules were consolidated into 9 distinct decision logic patterns: (1) According to the conversion table illustrated in Fig. 7, we translated all features and their thresholds from the 180 decision rules into the corresponding vital‑sign “Increase” or “Decrease” patterns. (2) If multiple rules for the same vital sign met the criteria for either an increase or a decrease, they were consolidated and treated as a single increase or decrease event (rules in which the same vital sign simultaneously exhibited both increasing and decreasing trends were excluded during rule selection).

### Evaluation Method

Performance of the trained random forest model was evaluated using the Area Under the Receiver Operating Characteristic Curve (AUROC) as well as the Area Under the Precision-Recall Curve (AUPRC). Since EAEWS employed fixed decision thresholds based on rule logic, Receiver Operating Characteristic Curve (ROC) and Precision-Recall Curve (PRC) analyses were not applicable. Instead, we evaluated alert performance of the machine learning models using SE, PPV, and F1 score from alert–event correlation analysis across predefined thresholds, in comparison with the EAEWS system. We calculated the difference between the first EAEWS system alert time and the CRI event time in a correct prediction as lead time.

For PPV, consecutive EAEWS rule triggers were counted as a single alert. However, if there was at least a gap of a minute between triggers, they were considered as distinct alerts^47^. For SE calculations, if a CRI event is successfully predicted, it was counted as a true positive prediction regardless of how many alerts were triggered in the 3 h leading up to the event. For consistency, we calculated it as the mean proportion of time with correct alerts, defined as the number of minutes with correct alerts divided by the total number of minutes from the first correct alert to event onset.

In this study, CRI events were defined as positive outcomes, while the periods during which patients remained clinically stable were treated as the negative segments.

For model interpretability, we analyzed feature contributions of the machine learning models using SHAP value, while the relative importance of features in the EAEWS system was assessed based on rule‑triggering frequency.

### Statistical analysis

Statistical analyses were conducted according to distribution characteristics of the data in this study. For normally distributed continuous variables, we reported means with standard deviations (SD); for non-normally distributed continuous variables, we reported medians with interquartile ranges [25th, 75th percentiles]. Categorical variables were summarized as frequencies and percentages. Group comparisons for categorical variables were performed using chi-square tests or Fisher’s exact test where appropriate, with results reported as relative risks with 95% confidence intervals. Continuous variables were compared using Student’s t-test, while Wilcoxon rank-sum test was used to analyze ordinal data. Confidence intervals (CIs) were estimated using a nonparametric bootstrap with resampling performed at the patient level (with replacement). Each bootstrap sample was of the same size as the original cohort, and 1000 iterations were conducted. The 95% CIs were derived from the 2.5th and 97.5th percentiles of the bootstrap distribution. For the random forest, XGBoost, GBM, and LSTM models, threshold-independent metrics (AUROC and AUPRC) were computed directly, whereas threshold-dependent metrics (PPV, sensitivity and F1 score) were calculated at the fixed threshold (for the random forest model, thresholds included the default value of 0.5, maximizing the F1 score, and maximizing the Youden index; for XGBoost, GBM, and LSTM, only the default threshold of 0.5 was used). For the EAEWS system, which outputs binary predictions, PPV, sensitivity and F1 score were computed directly.

Pairwise comparisons of AUROC and AUPRC between machine learning models were performed using a paired patient-level bootstrap (1000 iterations; sample size equal to the original cohort). The two-sided P values were calculated as the proportion of re-centered absolute differences greater than or equal to the observed absolute difference, with a continuity correction. Pairwise comparisons of binary classification outcomes for CRI prediction were performed using the McNemar test, based on discordant prediction pairs (i.e., cases correctly classified by one model but not the other). Statistical significance was defined as a two-sided *P* value < 0.05.

All analyses were conducted in Python (version 3.12). Data processing and statistical analyses were performed using NumPy v1.26.4 and Pandas packages v2.2.2. The trend feature extraction methods can be found in tsfeature time series analysis tool^49–51^. Predictive models were trained and evaluated with Scikit-learn v1.5.1, xgboost v2.1.2. Data visualization and model interpretation employed Matplotlib v3.9.2 and SHAP v0.47.1.

## Supplementary information

Supplementary0520

## Data Availability

Clinical datasets are not publicly available due to patient privacy considerations and commercial restrictions but may be requested from the corresponding authors with appropriate institutional approval.

## Code availability

The code and example MIMIC-III data supporting the main conclusions of this study are publicly available on GitHub (https://github.com/FAH-SYSU-SICU1-Datascience/Early-Prediction-of-CRI-with-EAML), excluding components subject to commercial or intellectual property constraints.
