# Supplementary material for: Expert Augmented Prediction of Circulatory and Respiratory Instability from High Resolution Vital Signs
Source: NPJ Digit Med. 2026 Jul 13;9:853. doi: 10.1038/s41746-026-02993-9 (PMC13737065; doi:10.1038/s41746-026-02993-9)
Supplement: Supplementary file 1 [file 41746_2026_2993_MOESM1_ESM.pdf]

Expert Augmented Prediction of Circulatory and Respiratory Instability from High Resolution Vital Signs

Supplementary data

Supplementary Content

Supplementary Note 1. Database and event labeling 3

Supplementary Note 2. Explanation of feature sources 4

Supplementary Note 3. Model Training and Feature Sets 5

Supplementary Table 1. MIMIC-III distribution of patients by nursing notes number 7

Supplementary Table 2. MIMIC-III distribution of nursing notes interval 8

Supplementary Table 3. SYSU-ICU distribution of patients by nursing notes number 9

Supplementary Table 4. SYSU-ICU distribution of nursing notes interval 10

Supplementary Table 5. MIMIC-III distribution of patients by laboratory measurements 11

Supplementary Table 6. MIMIC-III distribution of laboratory measurements interval 12

Supplementary Table 7. SYSU-ICU distribution of patients by laboratory measurements 13

Supplementary Table 8. SYSU-ICU distribution of laboratory measurements interval 14

Supplementary Table 9. Distribution of CRI events 15

Supplementary Table 10. Calculation of trend-based features 16

Supplementary Table 11. Calculation of baseline features 17

Supplementary Table 12. Feature description 18

Supplementary Table 13. Machine learning input features 19

Supplementary Table 14. Laboratory and basic parameters for model training 20

Supplementary Table 15. Trend before label in SYSU-ICU. 21

Supplementary Table 16. Trend before label in MIMIC-III 22

Supplementary Table 17. Statistics of Rule Parameter Composition. 23

Supplementary Table 18. Model performance of single-vital-sign features across resolutions in SYSU-ICU testing set. 24

Supplementary Table 19. Model performance of single-vital-sign features across resolutions in MIMIC-III 25

Supplementary Figure 1. EAEWS system development overview 26

Supplementary Figure 2. Feature extraction from high-resolution vital-sign time series. 27

Supplementary Figure 3. Temporal labeling strategy and the relationship between input features and events 28

Supplementary Figure 4. The ROC and PRC of different machine learning models using 40 vital features as input 29

Supplementary Figure 5. The ROC and PRC of different machine learning models using vital features incorporating laboratory and demographic features as input 30

Supplementary Figure 6. The ROC and PRC of random forest models using vital signs, laboratory, and demographic features 31
Supplementary Figure 7. The ROC and PRC of random forest models using different search windows........ 32
Supplementary Figure 8. Sensitivity, PPV, and F1 score of the random forest model using different search windows........ 33
Supplementary Figure 9. Comparison of feature contribution for difference data resolution. 34
Supplementary Figure 10. Comparative early-warning performance and feature contributions of the EAEWS and Random Forest models 35
Supplementary Figure 11. Circulatory- and respiratory-derived feature categories contributing to correct prediction of circulatory and respiratory instability in SYSU-ICU testing set........ 37
Supplementary Figure 12. The Sensitivity, PPV, and F1 score of EAEWS using different search windows 38
Supplementary Figure 13. Comparison of EAEWS and different machine learning models at the default threshold 39
Supplementary Figure 14. Distribution of EAEWS output consistency across lead time....... 40
Supplementary Figure 15. Comparison of AUROC and AUPRC improvement for 1-Second and 1-Minute Inputs Using Single Vital-Sign Features........ 41
Supplementary Figure 16. Sensitivity, PPV, and F1 score comparison between the circulatory instability (CI)-only and full EAEWS 42
Mandated checklist: TRIPOD_AI 43

Supplementary Note 1. Database and event labeling

Data quality was evaluated with particular attention to the completeness of intervention records and the availability of biochemical information. Based on this assessment, we selected the SYSU-ICU database for model development due to its higher data quality. The detailed analysis is summarized below.

For nursing notes, intervention events were aligned with patient waveforms and admission and discharge timestamps to ensure temporal consistency. For laboratory measurements, outliers in laboratory measurements results were excluded. Compared with the MIMIC-III database, the SYSU-ICU database provides more comprehensive nursing and laboratory documentation, characterized by a higher frequency of nursing entries per patient and shorter intervals between consecutive records.

During CRI event labeling, we primarily relied on predefined intervention criteria. For borderline cases requiring additional clinical context, supplementary nursing documentation and laboratory findings (Supplementary Tables 1–8) were systematically reviewed to identify emergency interventions versus routine medical orders. When calculating the intervals between nursing records or laboratory measurements, multiple entries within 3 minutes were treated as a single record to avoid redundancy.

We also analyzed the distribution of CRI events confirmed by experts (Supplementary Table 9). The proportion of expert-confirmed labels lacking corresponding clinical notes records was lower in the SYSU-ICU than in MIMIC-III. In SYSU-ICU, 73.9% of intervention events were preceded by documented hypotension or hypoxemia within 3 hours, while only 26.1% occurred without such preceding events; similar patterns were observed in MIMIC-III (70.3% vs. 29.7%).

Supplementary Note 2. Explanation of feature sources

Feature selection: Among cardiovascular parameters, a strong physiological correlation exists between SBP, diastolic blood pressure (DBP), and MAP, with each parameter providing distinct hemodynamic information. Empirical evidence from prior investigations demonstrates that SBP exhibits greater sensitivity than DBP and MAP in detecting early signs of clinical deterioration[1]. Therefore, based on its superior predictive performance and clinical interpretability, SBP was selected as the blood pressure variable for our model.

Supplementary Note 3. Model Training and Feature Sets

Laboratory measurements and patient demographic information were incorporated as supplementary inputs in the machine learning model performance comparison. This section describes the model training methodology and the specific feature sets used.

We constructed a random forest model using the SYSU-ICU training set and compared the differences before and after introducing laboratory measurements and basic information. We extracted a total of 43 MIMIC-III laboratory measurements and 206 SYSU-ICU laboratory measurements. After referring to the laboratory measurements in the[1,2], we took the intersection of all laboratory measurements, which is 25 indicators (Supplementary Table 14). All laboratory measurements were forward filled for up to 24 hours. We extract 6 features from demographic features based on expert recommends (Supplementary Table 14).

Using the SYSU-ICU training set, we developed and compared several original random forest models incorporating different combinations of input features: (1) vital signs only, (2) vital signs plus laboratory test results, (3) vital signs plus baseline information (demographic characteristics and comorbidities), and (4) all three feature types combined. The results showed adding laboratory and baseline variables yielded only modest gains in predictive performance, as measured by both AUROC and AUPRC, on the SYSU-ICU testing set and the external MIMIC-III cohort (Supplementary Figure 2). The AUROC increased from 0.83 to 0.86 on the MIMIC-III validation when both additional feature types were included.

Supplementary References:

1. Rahman A, Chang Y, Dong J, Conroy B, Natarajan A, Kinoshita T, Vicario F, Frassica J, Xu-Wilson M: **Early prediction of hemodynamic interventions in the intensive care unit using machine learning**. *Crit Care* 2021, **25**(1):388.
2. Hyland SL, Faltys M, Huser M, Lyu X, Gumbsch T, Esteban C, Bock C, Horn M, Moor M, Rieck B *et al*: **Early prediction of circulatory failure in the intensive care unit using machine learning**. *Nat Med* 2020, **26**(3):364-373.

Supplementary Table 1. MIMIC-III distribution of patients by nursing notes number

Nursing note number	Patient proportion (%)	Cumulate proportion (%)
**0**	52.11	52.11
**> 0**	7.42	59.52
**> 5**	7.42	66.94
**> 10**	9.21	76.15
**> 20**	11.15	87.30
**> 50**	7.27	94.57
**>100**	5.43	100

Supplementary Table 2. MIMIC-III distribution of nursing notes interval

Nursing note interval (h)	Patient proportion (%)	Cumulate proportion (%)
**< 1**	13.39	13.39
**< 4**	16.63	30.01
**< 8**	9.75	39.76
**< 12**	38.13	77.89
**< 24**	21.17	99.06
**≥ 24**	0.94	100

Supplementary Table 3. SYSU-ICU distribution of patients by nursing notes number

Nursing note number	Patient proportion (%)	Cumulate proportion (%)
**> 5**	3.70	3.70
**> 10**	14.10	17.80
**> 20**	25.26	43.07
**> 50**	21.15	64.22
**>100**	42.55	100

Supplementary Table 4. SYSU-ICU distribution of nursing notes interval

Nursing note interval (h)	Patient proportion (%)	Cumulate proportion (%)
**< 1**	56.60	56.60
**< 4**	35.38	91.39
**< 8**	6.64	98.62
**< 12**	1.35	99.98
**< 24**	0.01	99.99
**≥ 24**	0.01	100

Supplementary Table 5. MIMIC-III distribution of patients by laboratory measurements

measurements number	Patient proportion (%)	Cumulate proportion (%)
**0**	0.05	0.05
**> 0**	8.09	8.13
**> 10**	48.44	56.58
**> 50**	20.48	77.06
**> 100**	14.11	91.17
**> 200**	7.82	99.00
**> 500**	1.00	100

Supplementary Table 6. MIMIC-III distribution of laboratory measurements interval

measurements interval (h)	Patient proportion (%)	Cumulate proportion (%)
**< 1**	22.95	22.95
**< 4**	25.32	48.27
**< 8**	20.07	68.34
**< 12**	10.10	78.44
**< 24**	12.45	90.88
**≥ 24**	9.12	100

Supplementary Table 7. SYSU-ICU distribution of patients by laboratory measurements

measurements number	Patient proportion (%)	Cumulate proportion (%)
**> 0**	11.05	11.05
**> 10**	56.11	67.16
**> 50**	19.04	86.19
**> 100**	9.28	95.48
**> 200**	4.41	99.88
**> 500**	0.12	100

Supplementary Table 8. SYSU-ICU distribution of laboratory measurements interval

measurements interval (h)	Patient proportion (%)	Cumulate proportion (%)
**< 1**	27.84	27.84
**< 4**	19.54	47.39
**< 8**	25.01	72.40
**< 12**	9.80	82.19
**< 24**	6.50	88.70
**≥ 24**	11.30	100

Supplementary Table 9. Distribution of CRI events

Database	Label type	No clinical notes records-n (%)	With clinical notes records-n (%)
**SYSU-ICU**	CRI	1118 (24.3)	3490 (75.7)
	CI	1034 (22.4)	3052 (66.2)
	RI	181 (3.9)	470 (10.2)
**MIMIC-III**	CRI	778 (30.8)	1751 (69.2)
	CI	694 (27.4)	1474 (58.3)
	RI	208 (8.2)	275 (10.9)

Supplementary Table 10. Calculation of trend-based features

**Trend-based feature**	**Description**	**Calculation method**
The trend segment	The segment to calculate the trend feature	The most recent trend adaptive window to the current time
Start value	The value at the beginning of the trend change segment	Value at trend start position
End value	The value at the end of the trend change segment	Value at trend end position
Start position	The time or index where the trend change begins	Start time or index of segment
End position	The time or index where the trend changes ends	End time or index of segment
Duration	The duration of the trend change	End position minus start position
Amplitude	The amplitude difference between the start and end of the trend	Start value minus end value
Z value	Statistical significance of the trend change	Calculated using the Mann-Kendall (MK) method
Slope	The slope of the trend change segment	Obtained by linearly fitting the data and calculating the slope of the fitted line

Supplementary Table 11. Calculation of baseline features

Baseline feature	Description	Calculation method
Stable segment	Data segment with no significant physiological deterioration.	Identified when the absolute z-value of trend segment is less than a predefined threshold and duration exceeds a predefined threshold (set using training data and expert input).
Reference feature	Baseline or reference value for the parameter.	For each stable segment within the 12 hours preceding the current time point, extract the mode. The mean of these modes is taken as the reference feature.
Substitute reference	Substitute used when a patient's reference feature cannot be effectively calculated.	The mean of the parameter across the database is used if no effective reference feature can be determined for the patient.

Supplementary Table 12. Feature description

Feature dimension	Feature name	Feature description
Baseline features	reference feature	Baseline Value of Parameter
Trend-based features	end value	End Value of Parameter Trend Change
Trend-based features	z value	Z-Value of Parameter Trend
Trend-based features	slope	Slope of Parameter Trend
Trend-based features	duration	Duration of Parameter Trend
Trend-based features	amplitude	Amplitude Difference of Parameter Trend
Statistical features	variance	Variance of Parameter
Statistical features	mean	Mean of Parameter
Statistical features	maximum	Maximum Value of Parameter
Statistical features	minimum	Minimum Value of Parameter

Supplementary Table 13. Machine learning input features

Feature dimension	Feature name	
Baseline features	HR reference feature	BP reference feature
	RR reference feature	SpO_2 reference feature
Trend-based features	HR end value	BP end value
	RR end value	SpO_2 end value
Trend-based features	HR amplitude	BP amplitude
	RR amplitude	SpO_2 amplitude
Trend-based features	HR z value	BP z value
	RR z value	SpO_2 z value
Trend-based features	HR slope	BP slope
	RR slope	SpO_2 slope
Trend-based features	HR duration	BP duration
	RR duration	SpO_2 duration
Statistical features	HR variance	BP variance
	RR variance	SpO_2 variance
Statistical features	HR mean	BP mean
	RR mean	SpO_2 mean
Statistical features	HR maximum	BP maximum
	RR maximum	SpO_2 maximum
Statistical features	HR minimum	BP minimum
	RR minimum	SpO_2 minimum

Supplementary Table 14. Laboratory and basic parameters for model training

Groups	Parameters
Coagulation	INR(PT)
	Platelet Count
	PTT
Kidney	Urea Nitrogen
	Creatinine
Liver	Asparate Aminotransferase (AST)
	Bilirubin, Total
	Alanine Aminotransferase (ALT)
	Albumin
Infection	White Blood Cells
	C-Reactive Protein
Respiratory	pCO2
	pO2
Environment	Potassium, Whole Blood
	pH
	Sodium, Whole Blood
	Lactate
	Base Excess
	Calculated Total CO2
	Hemoglobin
	Hematocrit
	Calcium, Total
	Phosphate
	Chloride, Whole Blood
	Glucose
Demographic	Sex
	Age
	COPD
	Heart Failure
	Hypertension
	Coronary heart disease

Supplementary Table 15. Trend before label in SYSU-ICU.

We evaluated changes in vital signs during the 30-minute period before CRI events. A vital sign was classified as changed (positive) if its variation during this period exceeded the prespecified threshold.

Vital-sign	Variation threshold	Positive	Negative	Proportion (%)
	**SYSU-ICU RI-label-only**			
**SBP**	>=30	255	267	48.85
**HR**	>=20	237	285	45.40
**RR**	>=12	219	303	41.95
**SPO2**	>=5	419	103	80.27
**SPO2-RR-only**	SPO2>=5 & RR>=12 & SBP<30 & HR<20	37	485	7.09
	**SYSU-ICU CI-label-only**			
**SBP**	>=30	1909	2048	48.24
**HR**	>=20	1126	2831	28.46
**RR**	>=12	880	3077	22.24
**SPO2**	>=5	843	3114	21.30
**SPO2-RR-only**	SPO2>=5 & RR>=12 & SBP<30 &HR<20	54	3903	1.36
	**SYSU-ICU CRI-label-only**			
**SBP**	>=30	71	58	55.04
**HR**	>=20	65	64	50.39
**RR**	>=12	43	86	33.33
**SPO2**	>=5	79	50	61.24
**SPO2-RR-only**	SPO2>=5 & RR>=12 & SBP<30 &HR<20	3	126	2.33

Supplementary Table 16. Trend before label in MIMIC-III.

Vital-sign	Variation threshold	Positive	Negative	Proportion (%)
		**MIMIC-III RI-label-only**		
**SBP**	>=30	148	213	41.00
**HR**	>=20	120	241	33.24
**RR**	>=12	126	235	34.90
**SPO2**	>=5	246	115	68.14
**SPO2-RR-only**	SPO2>=5 & RR>=12 & SBP<30 & HR<20	26	335	7.20
		**MIMIC-III CI-label-only**		
**SBP**	>=30	1123	923	54.89
**HR**	>=20	562	1484	27.47
**RR**	>=12	470	1576	22.97
**SPO2**	>=5	493	1553	24.10
**SPO2-RR-only**	SPO2>=5 & RR>=12 & SBP<30 &HR<20	47	1999	2.30
		**MIMIC-III CRI-label-only**		
**SBP**	>=30	48	74	39.34
**HR**	>=20	41	81	33.61
**RR**	>=12	47	75	38.52
**SPO2**	>=5	66	56	54.10
**SPO2-RR-only**	SPO2>=5 & RR>=12 & SBP<30 &HR<20	8	114	6.56

Supplementary Table 17. Statistics of Rule Parameter Composition.

Across the three stages of LASSO regression, rule selection, and rule retraining, the number of rules relying exclusively on respiratory variables progressively decreased from 11 to 6 and finally to 0.

Rule Source	Rule Num	SBP	HR	SPO2	RR	SPO2&RR Only
**LASSO regression**	389	306	319	351	247	11
**Rule Selection**	180	152	156	124	80	6
**Rule Retraining**	189	180	180	68	49	0

Supplementary Table 18. Model performance of single-vital-sign features across resolutions in SYSU-ICU testing set.

Random forest models were constructed using SBP-, HR-, SpO2-, and RR-related variables separately as input features, and their performance was compared across different temporal resolutions.

Resolution	AUROC	95%CI	AUPRC	95%CI
		**Model input feature: SPO2 features**		
**1 second**	0.723	(0.774,0.797)	0.138	(0.125,0.150)
**1 minute**	0.703	(0.772,0.790)	0.129	(0.116,0.142)
**30 minutes**	0.685	(0.768,0.784)	0.114	(0.094,0.126)
**1 hour**	0.678	(0.756,0.773)	0.105	(0.086,0.124)
		**Model input feature: SBP features**		
**1 second**	0.786	(0.781,0.806)	0.158	(0.132,0.183)
**1 minute**	0.781	(0.773,0.793)	0.150	(0.129,0.167)
**30 minutes**	0.776	(0.746,0.774)	0.135	(0.117,0.153)
**1 hour**	0.764	(0.734,0.762)	0.129	(0.111,0.147)
		**Model input feature: HR features**		
**1 second**	0.793	(0.690,0.746)	0.146	(0.133,0.159)
**1 minute**	0.783	(0.682,0.715)	0.138	(0.126,0.151)
**30 minutes**	0.760	(0.648,0.689)	0.117	(0.107,0.128)
**1 hour**	0.748	(0.652,0.697)	0.113	(0.099,0.126)
		**Model input feature: RR features**		
**1 second**	0.689	(0.679,0.700)	0.067	(0.057,0.076)
**1 minute**	0.679	(0.665,0.693)	0.061	(0.051,0.071)
**30 minutes**	0.671	(0.658,0.684)	0.045	(0.039,0.052)
**1 hour**	0.667	(0.653,0.681)	0.038	(0.032,0.043)

Supplementary Table 19. Model performance of single-vital-sign features across resolutions in MIMIC-III

Resolution	AUROC	95%CI	AUPRC	95%CI
		**Model input feature: SPO2 features**		
**1 second**	0.749	(0.737,0.757)	0.143	(0.121,0.165)
**1 minute**	0.741	(0.733,0.749)	0.130	(0.109,0.151)
**30 minutes**	0.729	(0.717,0.742)	0.113	(0.104,0.115)
**1 hour**	0.719	(0.706,0.732)	0.098	(0.090,0.106)
		**Model input feature: SBP features**		
**1 second**	0.733	(0.725,0.741)	0.147	(0.136,0.157)
**1 minute**	0.727	(0.718,0.734)	0.140	(0.130,0.150)
**30 minutes**	0.720	(0.712,0.729)	0.130	(0.122,0.135)
**1 hour**	0.715	(0.708,0.723)	0.123	(0.112,0.131)
		**Model input feature: HR features**		
**1 second**	0.760	(0.748,0.772)	0.109	(0.095,0.123)
**1 minute**	0.755	(0.743,0.768)	0.102	(0.090,0.114)
**30 minutes**	0.741	(0.732,0.749)	0.088	(0.080,0.095)
**1 hour**	0.738	(0.729,0.748)	0.080	(0.073,0.087)
		**Model input feature: RR features**		
**1 second**	0.728	(0.714,0.741)	0.052	(0.048,0.056)
**1 minute**	0.720	(0.707,0.733)	0.044	(0.039,0.048)
**30 minutes**	0.713	(0.704,0.722)	0.030	(0.028,0.033)
**1 hour**	0.709	(0.700,0.718)	0.029	(0.027,0.032)

Supplementary Figure 1. EAEWS system development overview

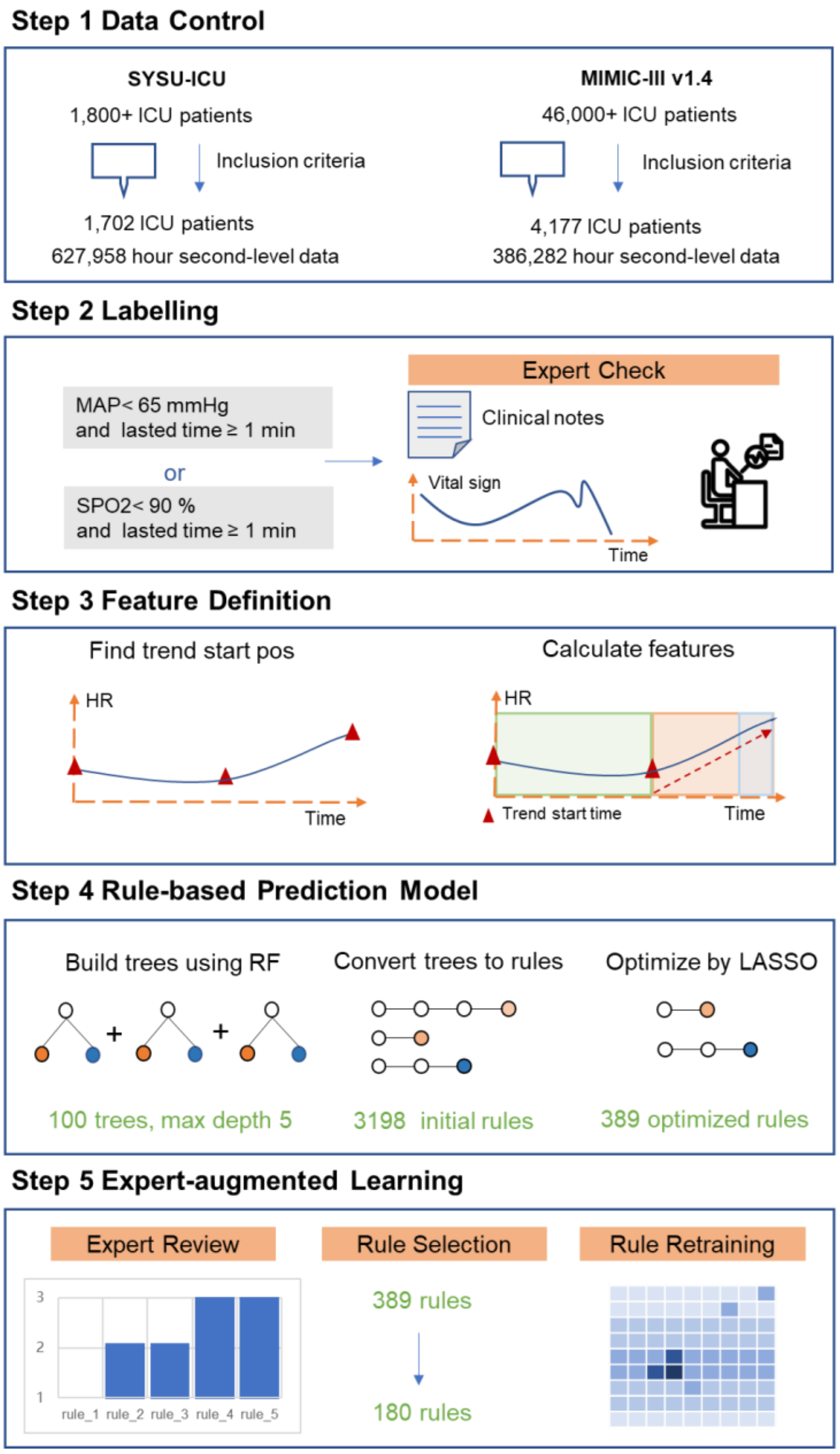

Step1: Patient data were screened based on the inclusion criteria. **Step2**: Circulatory and respiratory instability events, indicated by hypotension and hypoxia, were first flagged using vital-sign data and then further confirmed through electronic medical records and expert verification. **Step3**: We extracted baseline features, trend features, and statistical features from vital sign. **Step4**: A random forest model was trained and converted into rule-based forms, LASSO regression was applied to filter the rules. **Step5**: Rules were initially sorted by model's MSE and scored by experts, receiving the lowest expert score and lowest MSE were removed. The feature composition of each rule was reconstructed by experts. Using grid search method to find the best thresholds of reconstructed rules. RF: Random Forest.

Supplementary Figure 2. Feature extraction from high-resolution vital-sign time series.

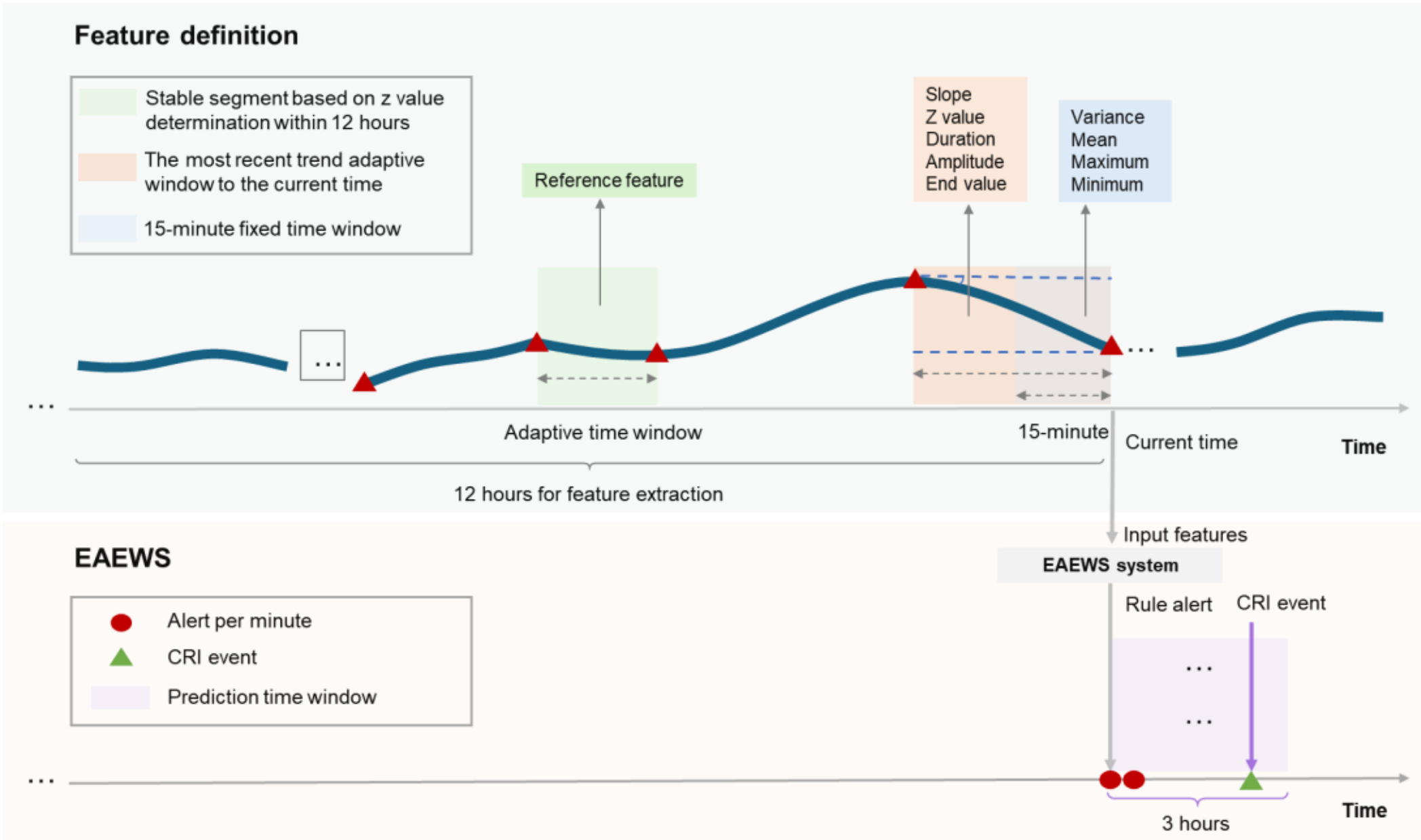

Trend change points in each vital-sign time series were identified and are indicated by red triangles, partitioning the series into consecutive trend segments. For each segment, slope, z-value, duration, amplitude, and end value were computed. Within the 12 hours preceding each prediction time point, segments with z-values below a predefined threshold (shown in green) were used to derive baseline (reference) features. For each prediction, the slope, z-value, duration, amplitude, and end value of the most recent segment (shown in orange) were used as trend-based features. In addition, statistical features—including variance, mean, maximum, and minimum—were calculated from a fixed 15-minute window immediately preceding the prediction time (shown in blue). Feature extraction was performed at a 1-minute resolution, and the resulting features were used as inputs to the Expert-Augmented Early Warning System (EAEWS).

Supplementary Figure 3. Temporal labeling strategy and the relationship between input features and events

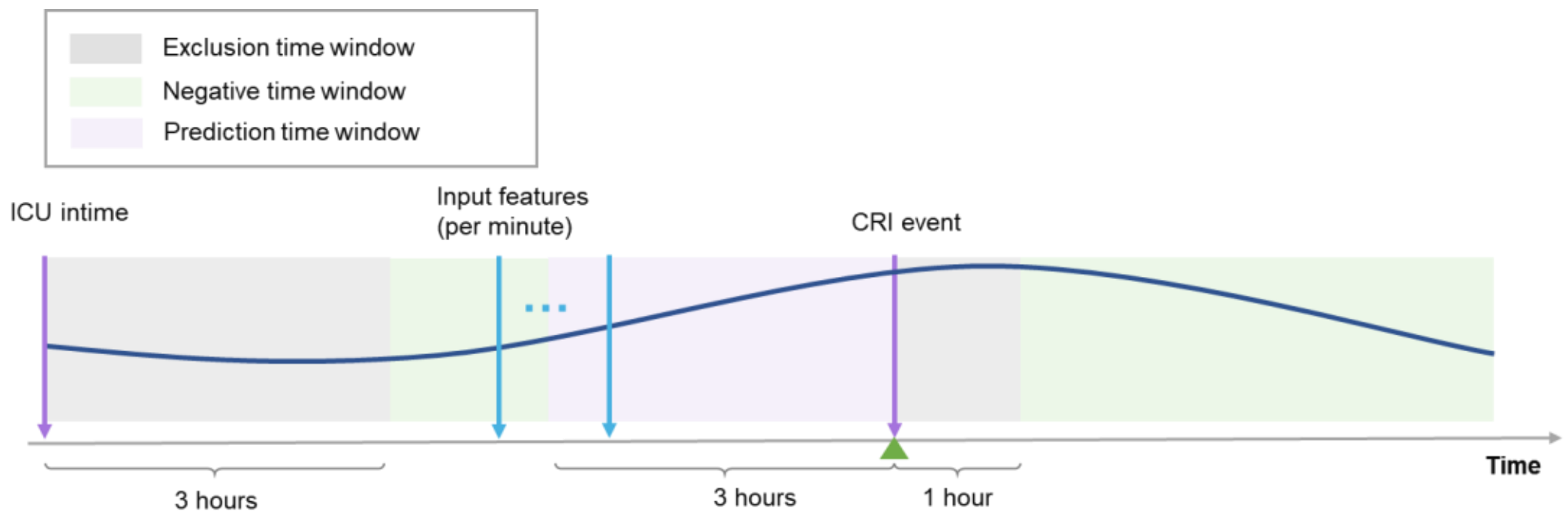

For patients with CRI events, these exclusion windows included the 3 hours preceding and 1 hour following each event, as well as the first 3 hours after ICU admission. For patients without CRI events, only the first 3 hours after ICU admission were excluded. All remaining time segments were labeled as negative. Outside the excluded intervals, 40 predefined features were computed at 1-minute resolution as inputs, and predictions were generated at each minute.

Supplementary Figure 4. The ROC and PRC of different machine learning models using 40 vital features as input

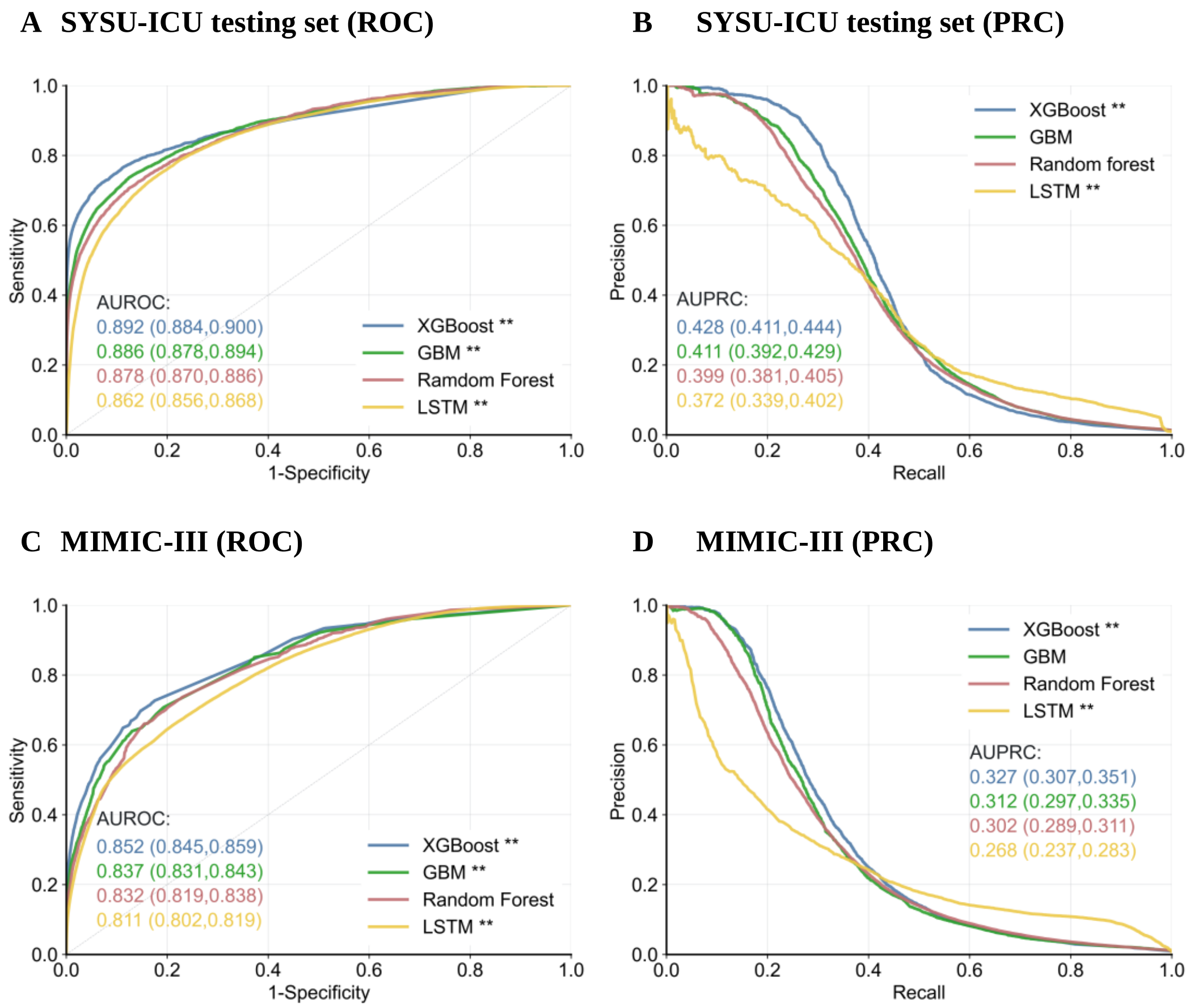

(A) Receiver operating characteristic (ROC) curves for CRI prediction in the SYSU-ICU testing set using different models trained on same feature sets. **(B)** Precision-recall curves (PRC) for the SYSU-ICU testing set corresponding to panel A. **(C)** ROC curves for CRI prediction in the external MIMIC-III cohort. **(D)** Precision-recall curves for the MIMIC-III cohort corresponding to panel C. **Model:** (1) eXtreme Gradient Boosting **(XGBoost)**, using 40 predefined features extracted from high-resolution vital sign data only; (2) Gradient Boosting Machine **(GBM)**, using the 40 vital-sign features extracted from high-resolution vital sign data only; (3) **Random Forest**, using the 40 vital-sign features extracted from high-resolution vital sign data only; and (4) Long Short-Term Memory (**LSTM)**, using 40 vital-sign features extracted from high-resolution vital sign data only; the input features of the LSTM are normalized, and the LSTM model makes a prediction at 5-minute intervals. *** Denotes statistical significance compared to the Random Forest method. Comparisons were performed using the bootstrap method with 1,000 resampling iterations. *p < 0.05, ** p < 0.01.**

Supplementary Figure 5. The ROC and PRC of different machine learning models using vital features incorporating laboratory and demographic features as input

A SYSU-ICU testing set (ROC)

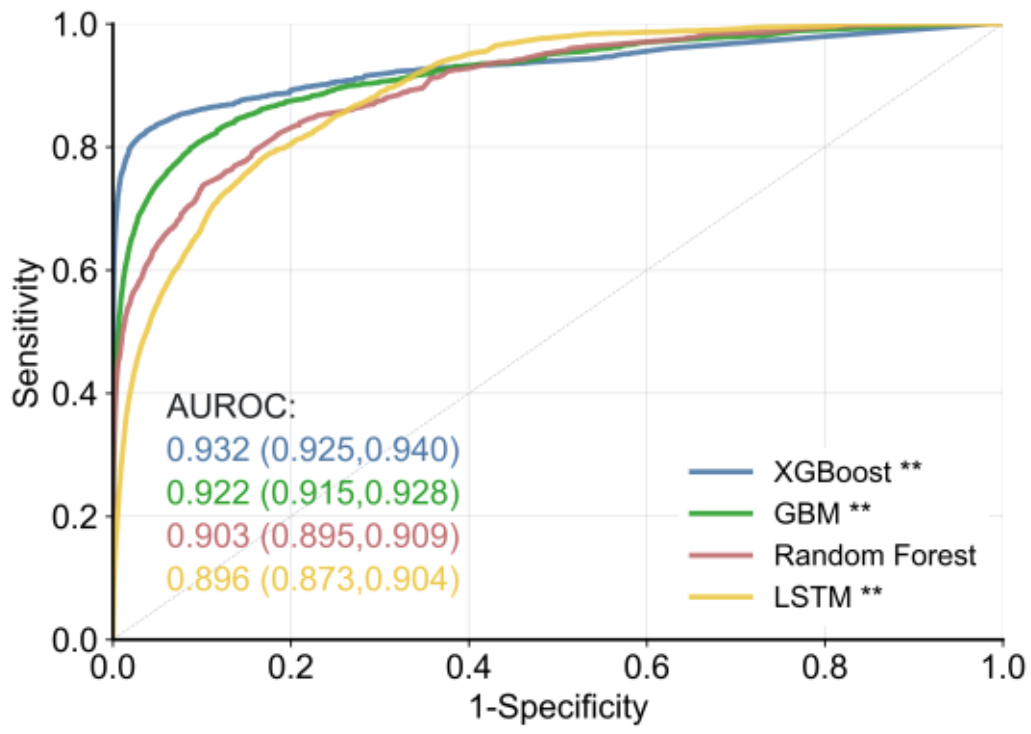

B SYSU-ICU testing set (PRC)

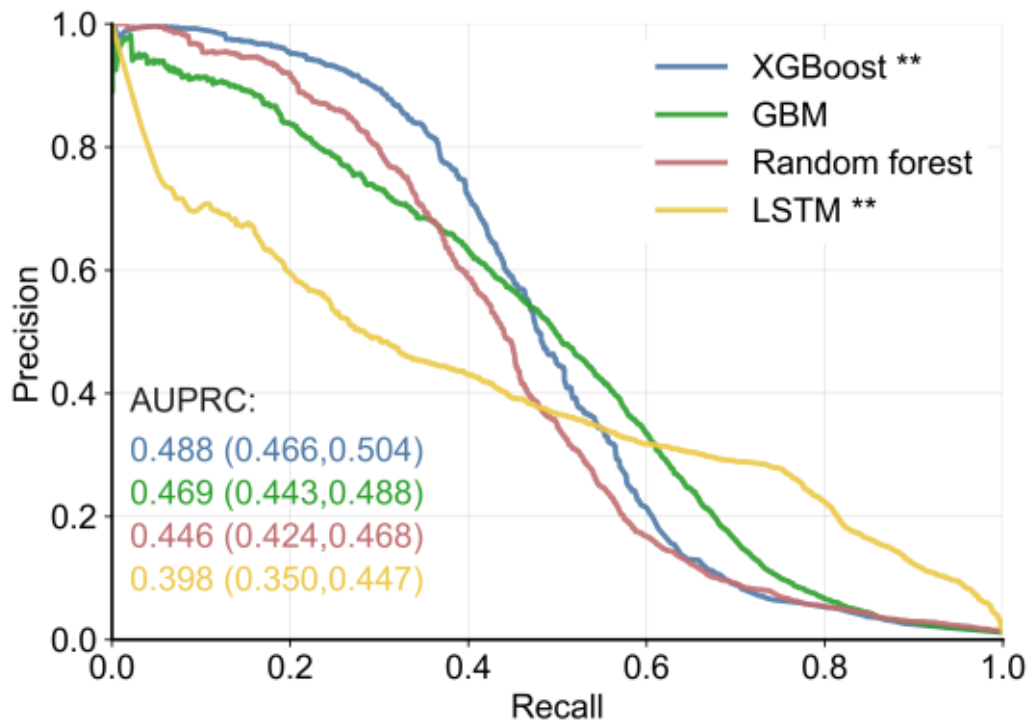

C MIMIC-III (ROC)

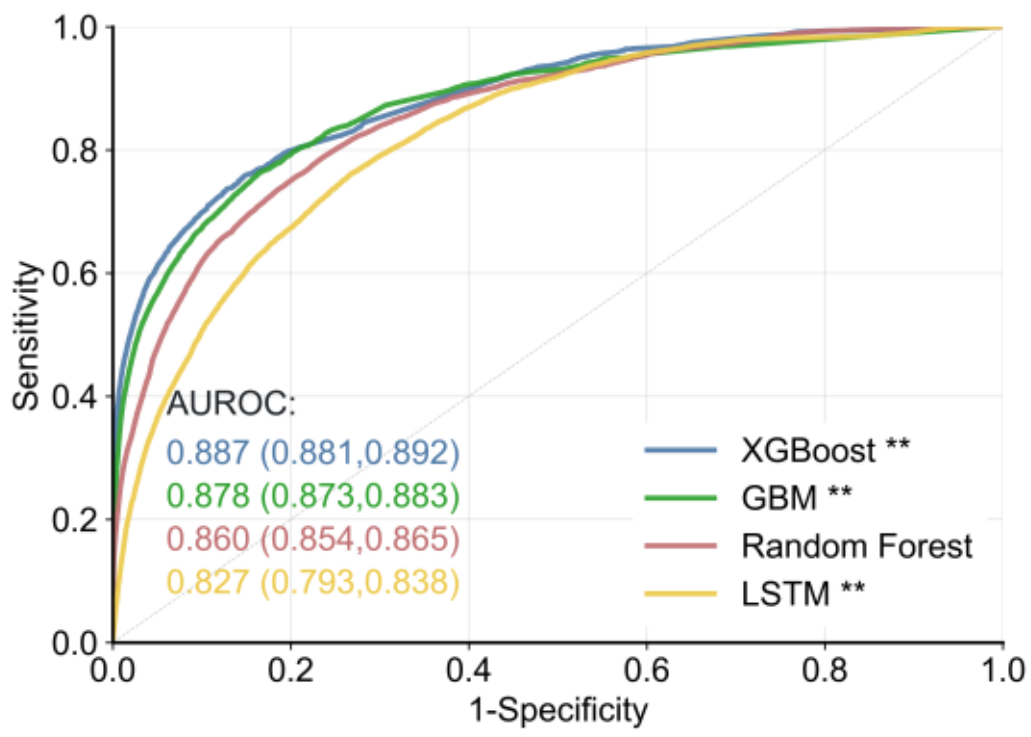

D MIMIC-III (PRC)

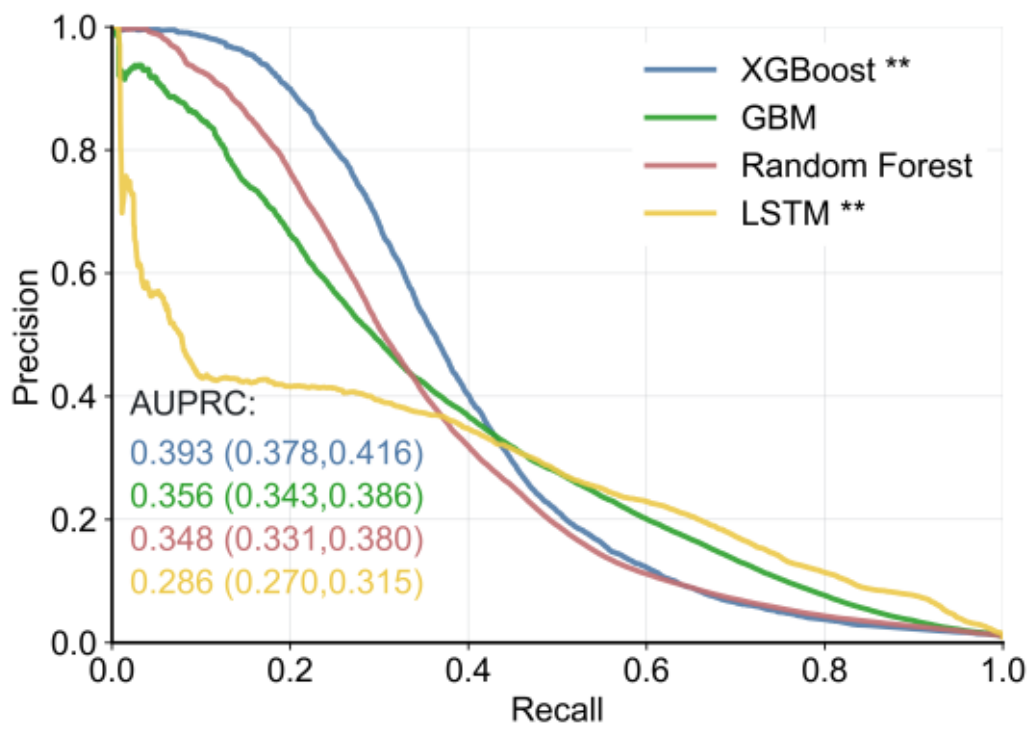

(A) Receiver operating characteristic (ROC) curves for CRI prediction in the SYSU-ICU testing set using different models trained on Vital + Lab + Base feature sets. **(B)** Precision-recall curves (PRC) for the SYSU-ICU testing set corresponding to panel A. **(C)** ROC curves for CRI prediction in the external MIMIC-III cohort. **(D)** Precision-recall curves for the MIMIC-III cohort corresponding to panel C. **Model:** (1) **XGBoost**, integrating 40 vital-sign features, 6 demographic features, and 25 laboratory-measurement features; (2) **GBM**, integrating 40 vital-sign features, 6 demographic features, and 25 laboratory-measurement features; (3) **Random Forest**, integrating 40 vital-sign features, 6 demographic features, and 25 laboratory-measurement features; and (4) **LSTM**, integrating 40 vital-sign features, 6 demographic features, and 25 laboratory-measurement features; The input features of the LSTM are normalized, and the model makes a prediction at 5-minute intervals. *** Denotes statistical significance compared to the Random Forest method. Comparisons were performed using the bootstrap method with 1,000 resampling iterations. *p < 0.05, ** p < 0.01.**

Supplementary Figure 6. The ROC and PRC of random forest models using vital signs, laboratory, and demographic features

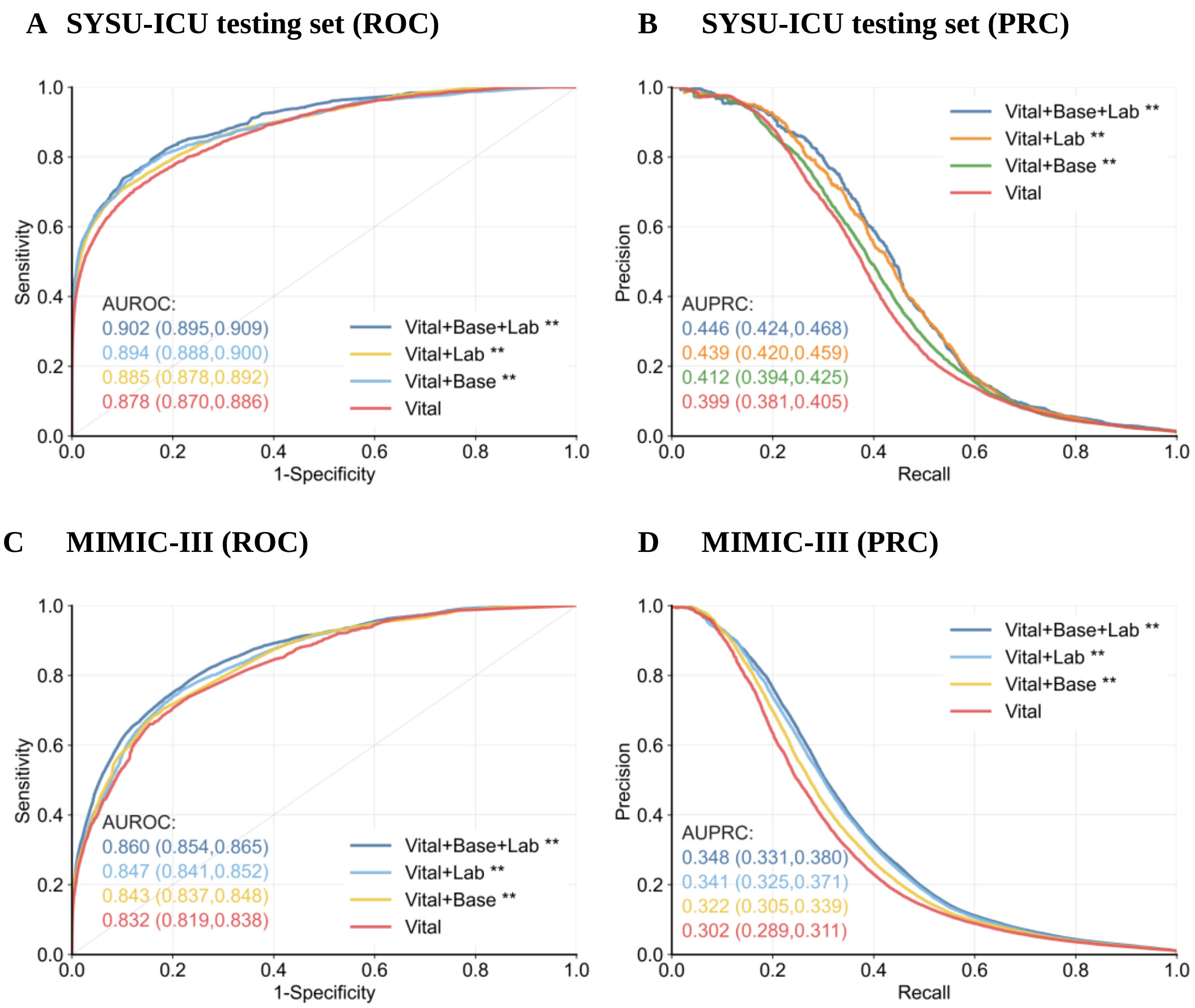

(A) Receiver operating characteristic (ROC) curves for CRI prediction in the SYSU-ICU testing set using random forest models trained on different feature sets. **(B)** Precision-recall curves (PRC) for the SYSU-ICU testing set corresponding to panel A. **(C)** ROC curves for CRI prediction in the external MIMIC-III cohort. **(D)** Precision-recall curves for the MIMIC-III cohort corresponding to panel C. **Model feature sets:** (1) **Vital**, using 40 features extracted from high-resolution vital sign data only; (2) **Vital + Lab**, combining the 40 vital-sign features with 25 laboratory-measurement features; (3) **Vital + Base**, combining the 40 vital-sign features with 6 demographic features; and (4) **Vital + Base + Lab**, integrating 40 vital-sign features, 6 demographic features, and 25 laboratory-measurement features. *** Denotes statistical significance compared to the Vital model. Comparisons were performed using the bootstrap method with 1,000 resampling iterations. *p < 0.05, ** p < 0.01.**

Supplementary Figure 7. The ROC and PRC of random forest models using different search windows

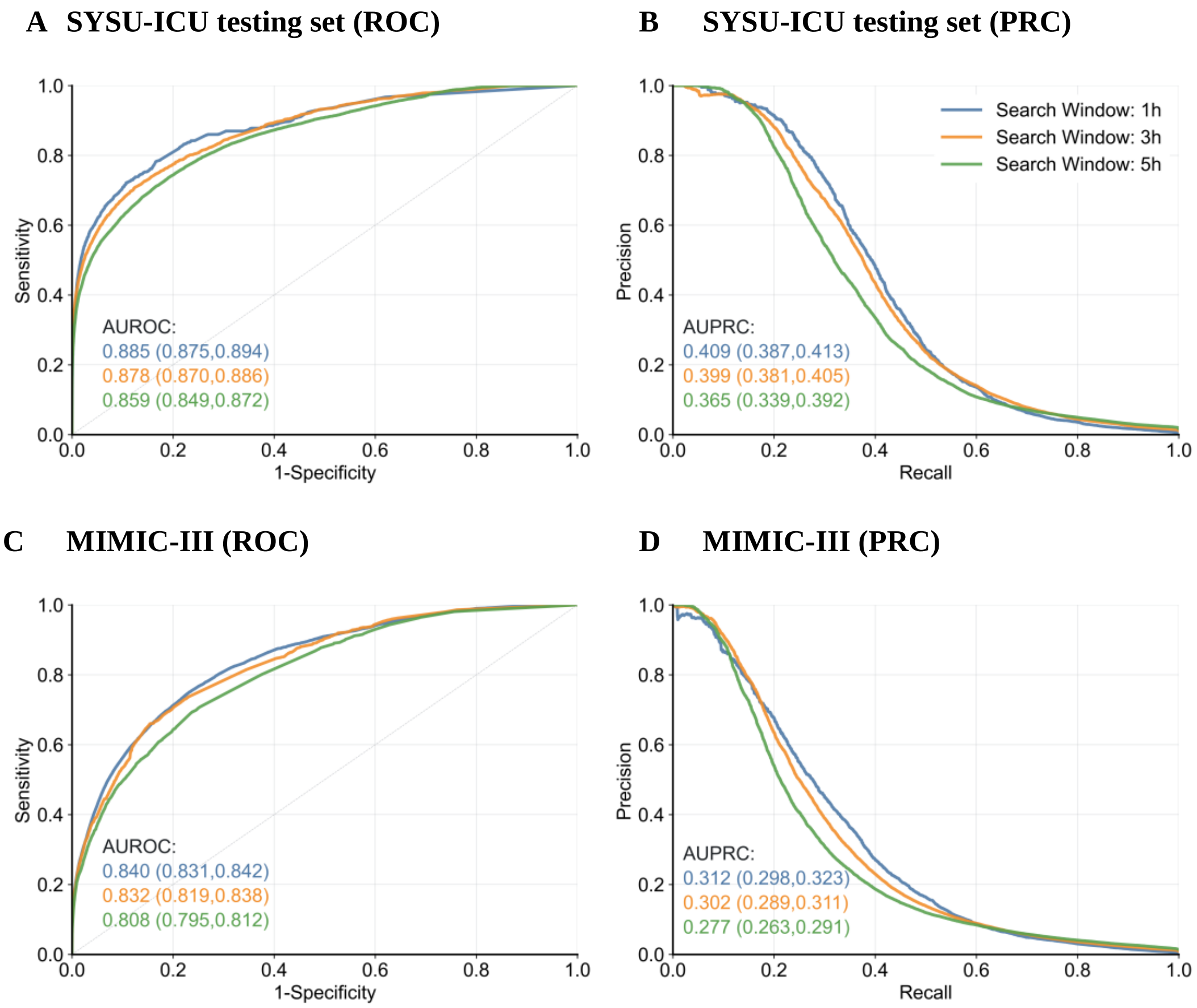

(A) Receiver operating characteristic (ROC) curves for CRI prediction in the SYSU-ICU testing set using random forest models trained on different search window before positive events. **(B)** Precision-recall curves (PRC) for the SYSU-ICU testing set corresponding to panel A. **(C)** ROC curves for CRI prediction in the external MIMIC-III cohort. **(D)** Precision-recall curves for the MIMIC-III cohort corresponding to panel C. **Model search window sets:** (1) **1 h**, 1-hour search window prior to a CRI event was defined, using only 40 features derived from high-resolution vital sign data; (2) **3 h**, 3-hour search window prior to a CRI event was defined, using only 40 features derived from high-resolution vital sign data; (3) **5 h**, 5-hour search window prior to a CRI event was defined, using only 40 features derived from high-resolution vital sign data. The 95% confidence intervals (CIs) were estimated using the bootstrap method with 1,000 resamples.

Supplementary Figure 8. Sensitivity, PPV, and F1 score of the random forest model using different search windows

A SYSU-ICU testing set

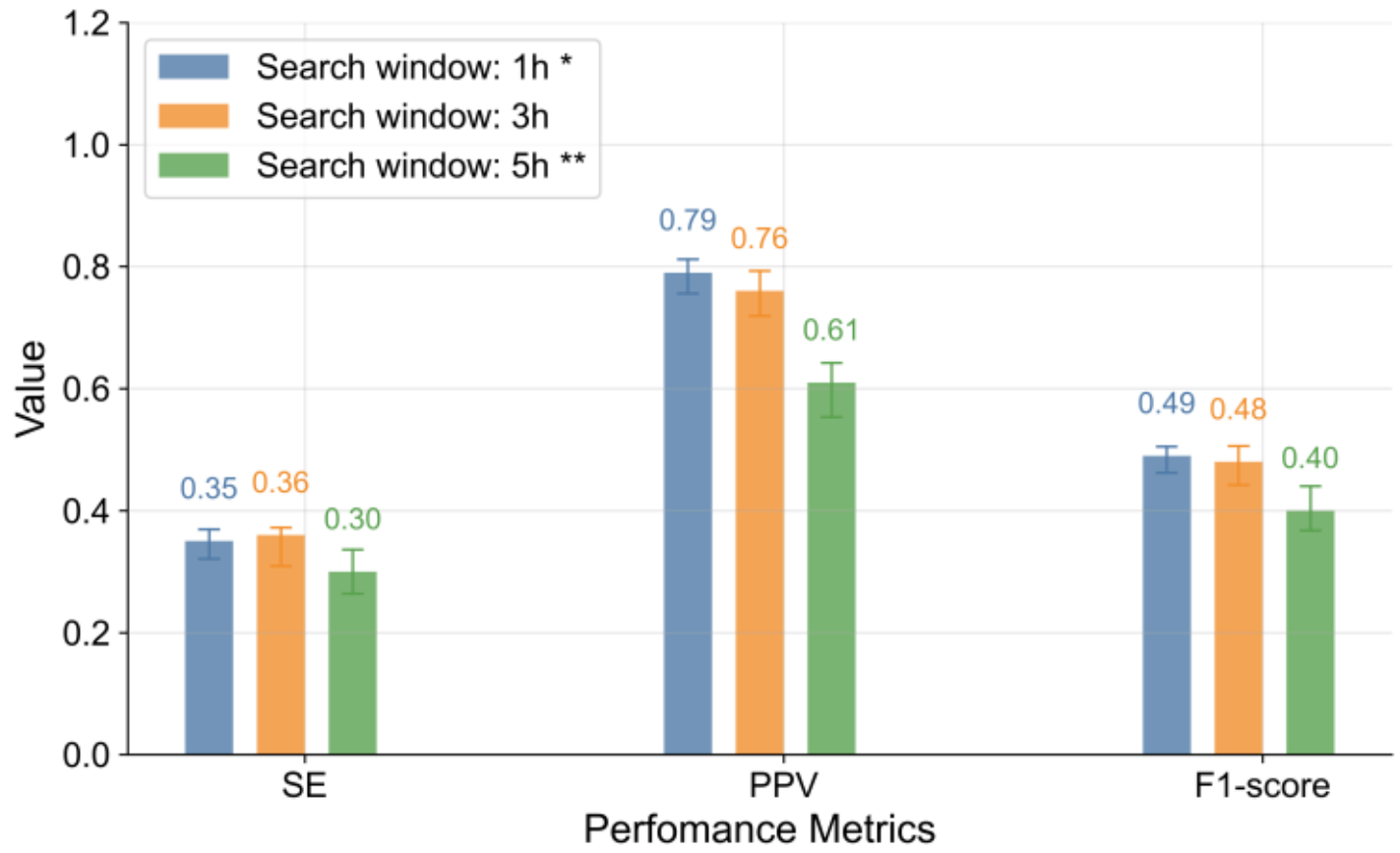

B MIMIC-III

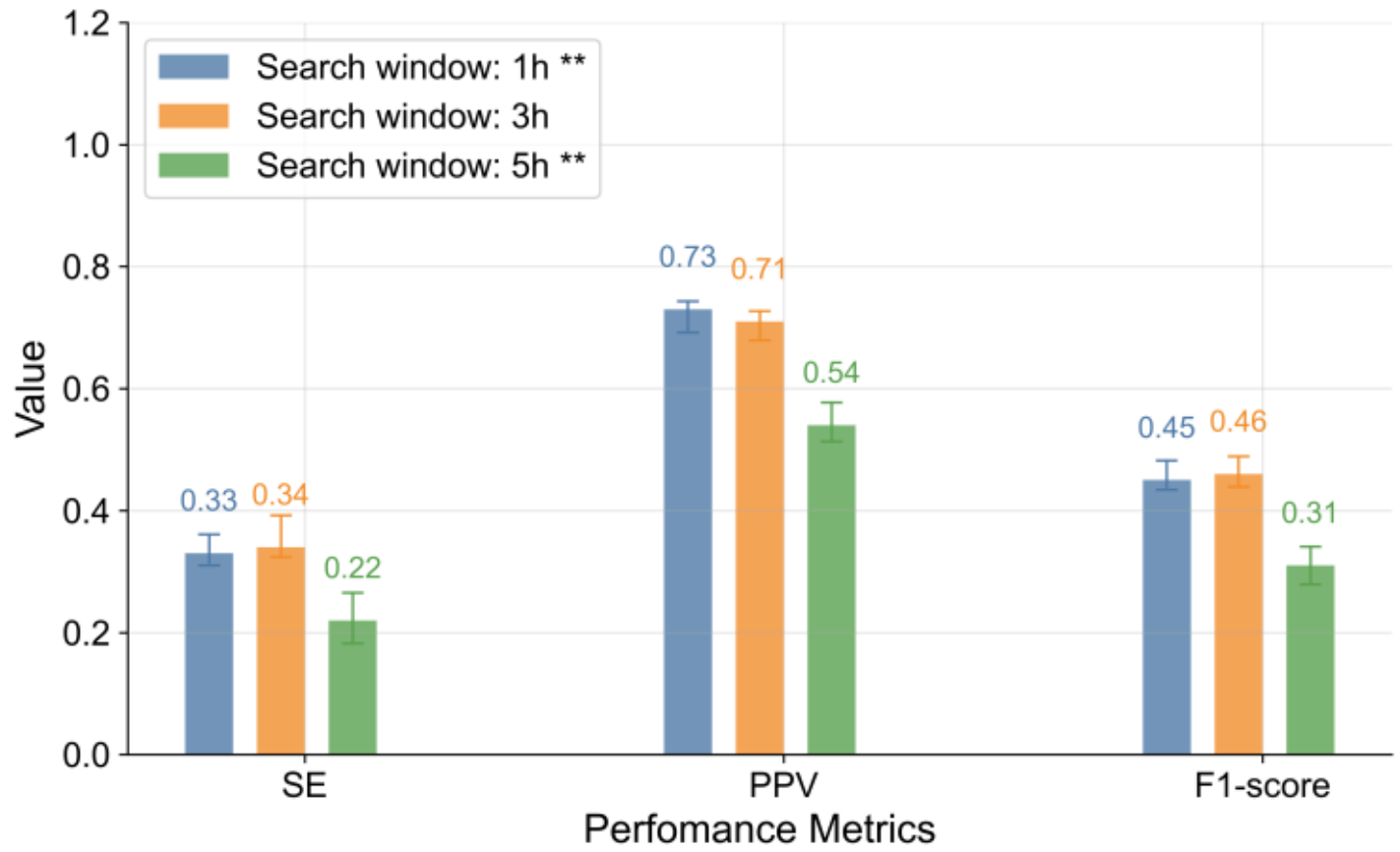

(A) Performance comparison of the random forest models trained on different search window under different search windows (1h, 3h, and 5h) in testing set. (B) Performance comparison of the random forest models under different search windows (1h, 3h, and 5h) in MIMIC-III. Performance was assessed using sensitivity (SE), positive predictive value (PPV), and F1 score at the default threshold of 0.5. Error bars represent 95% confidence intervals (CIs) for the differences. **McNemar test was applied to compare search window:3h with other search windows. *p < 0.05, ** p < 0.01.**

Supplementary Figure 9. Comparison of feature contribution for difference data resolution

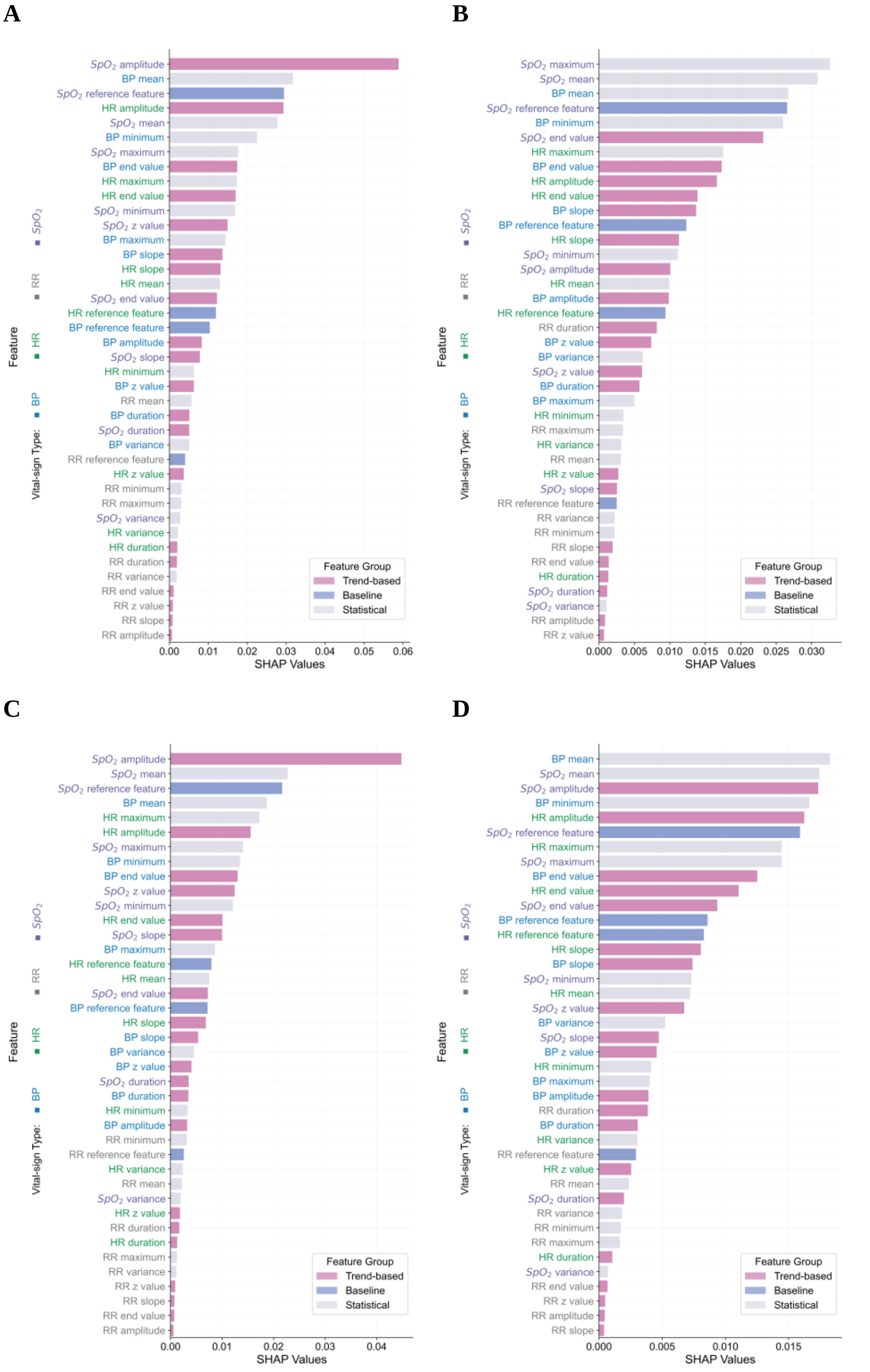

(A) SHAP analysis of machine-learning feature importance in the SYSU-ICU testing set using 1-second–resolution vital-sign data and 40 predefined vital features (**Methods**). **(B)** SHAP values feature importance in the SYSU-ICU testing set using the corresponding 1-minute–resolution data (panel A). **(C)** SHAP values feature importance in the external MIMIC-III cohort using 1-second–resolution data. **(D)** SHAP values feature importance in the external MIMIC-III cohort using the corresponding 1-minute–resolution data (panel C).

Supplementary Figure 10. Comparative early-warning performance and feature contributions of the EAEWS and Random Forest models

A

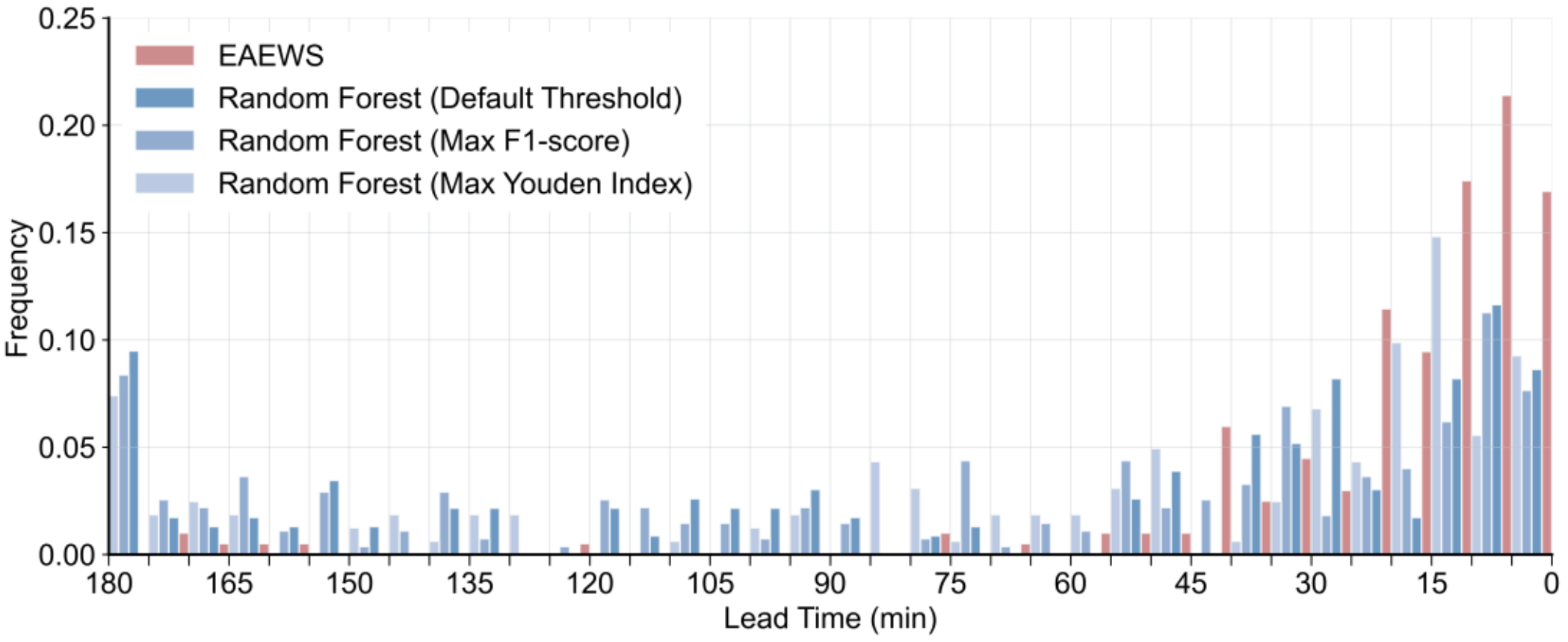

B

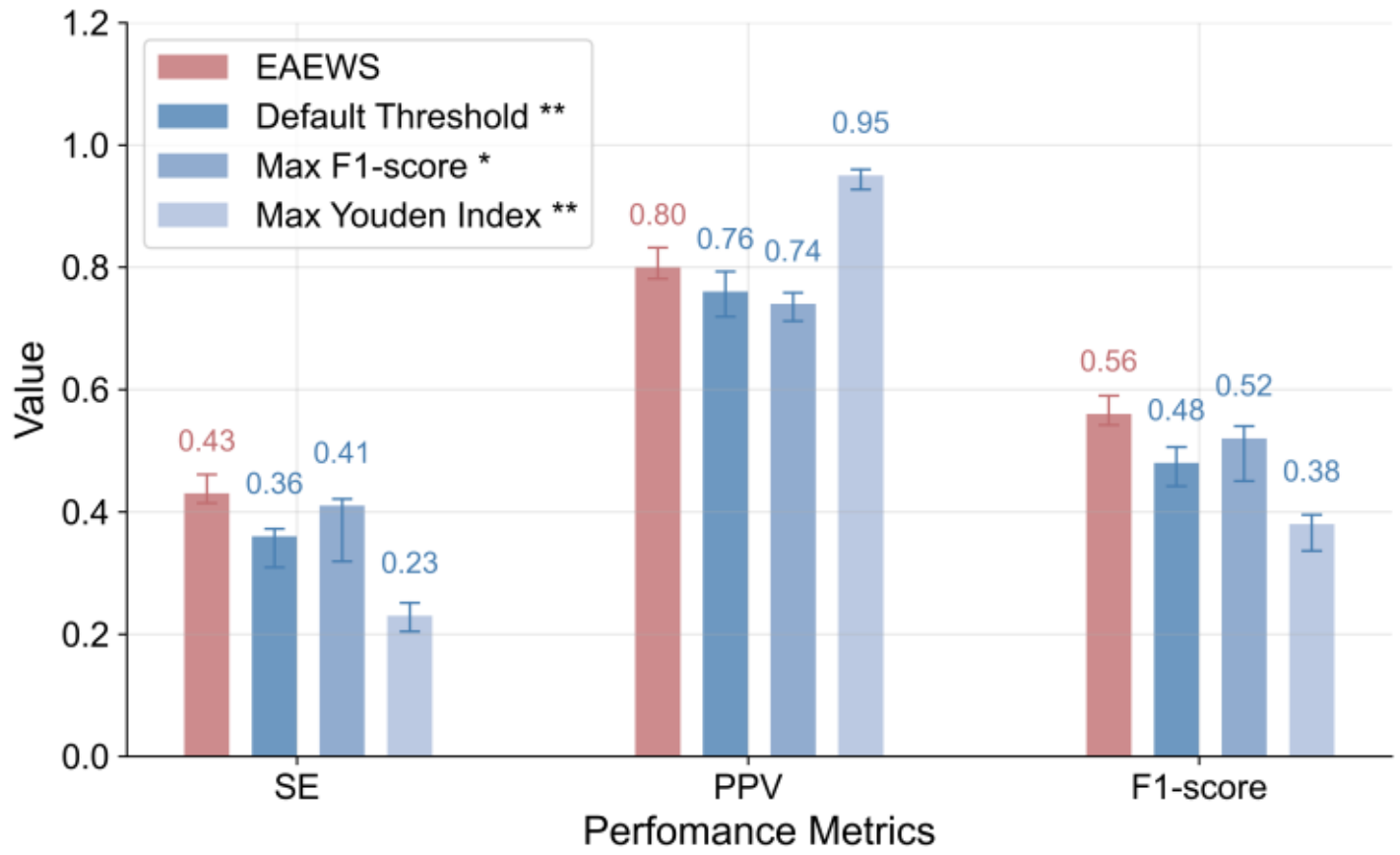

C

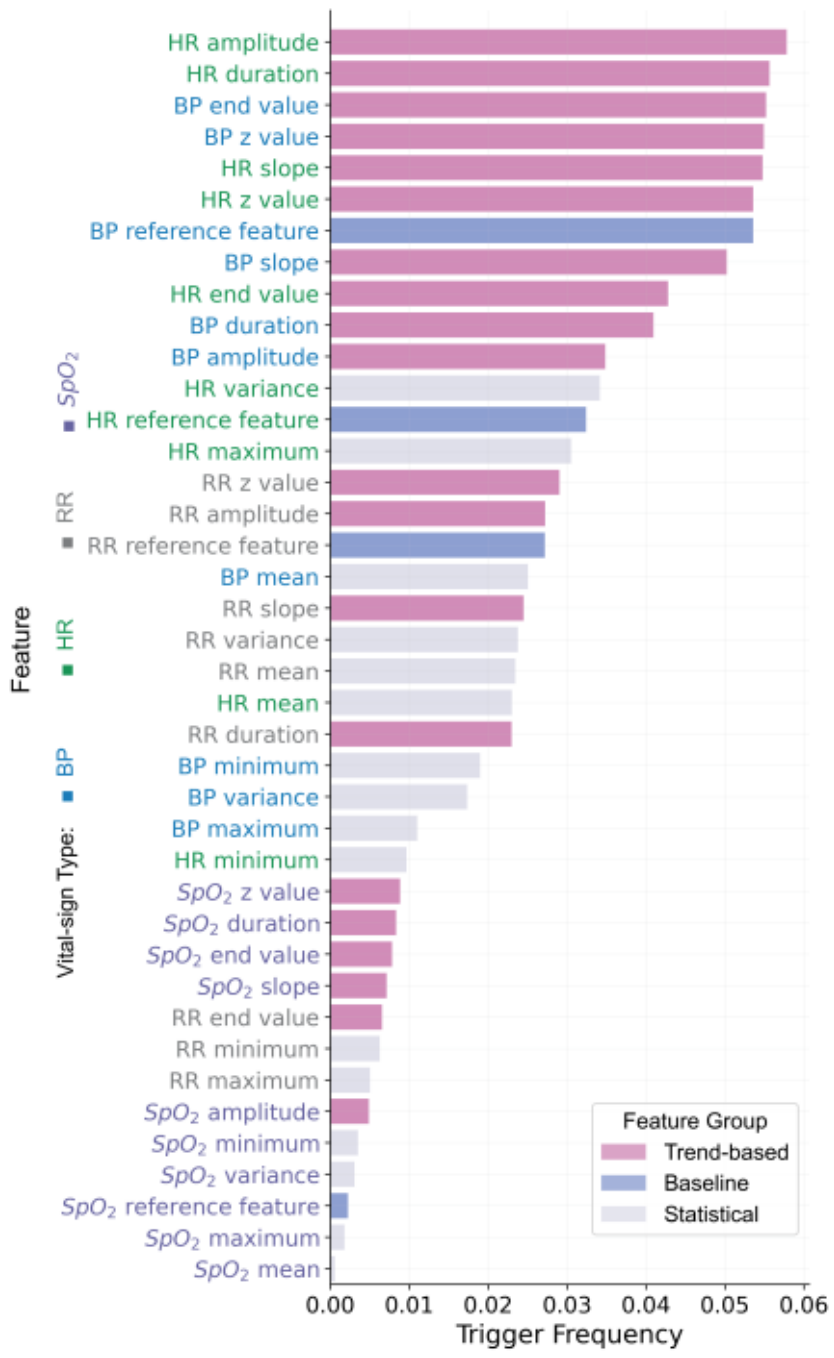

D

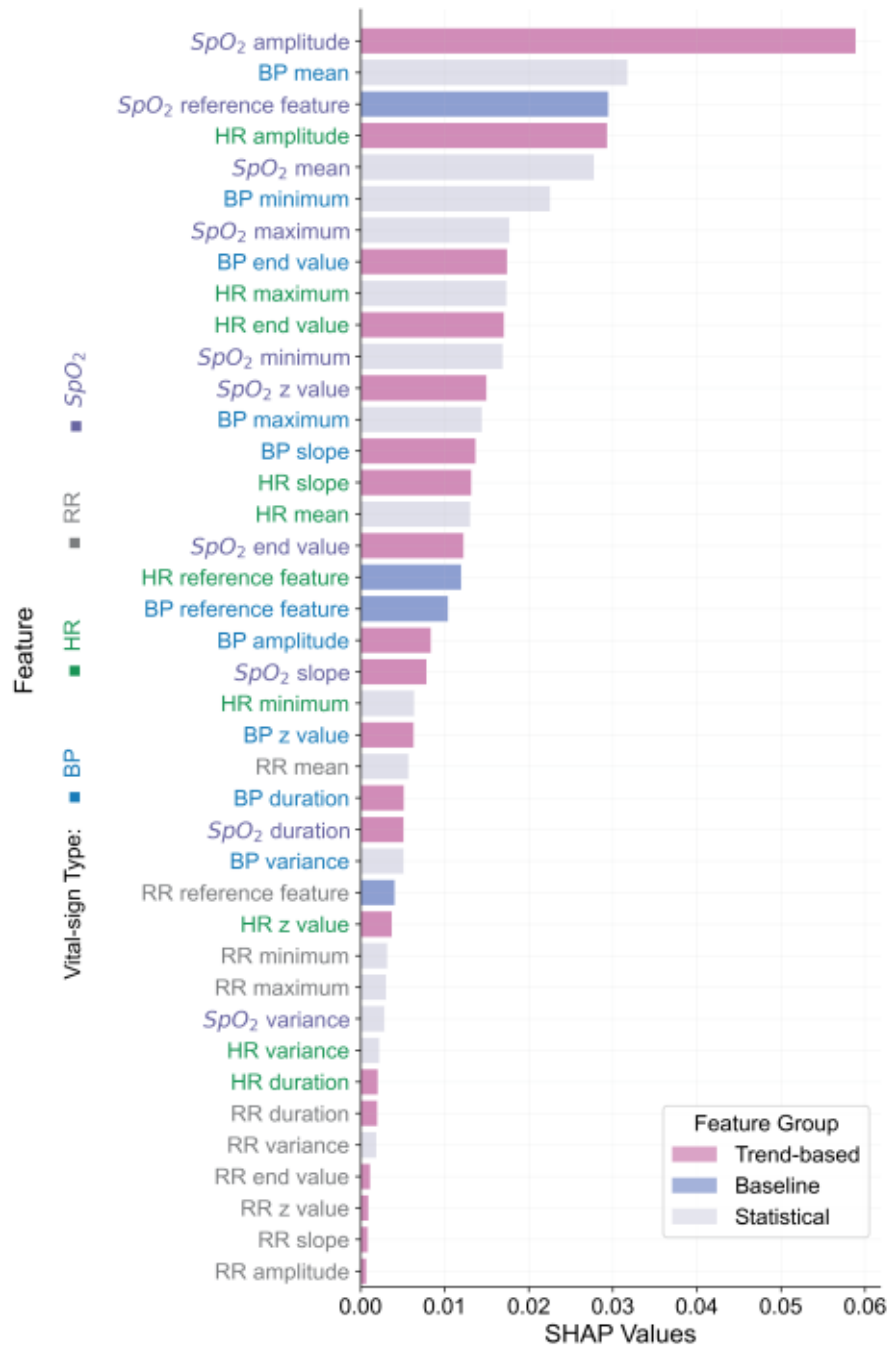

(A). Distribution of early warning lead times for true-positive CRI events in external validation. For each true positive predicted CRI event in the SYSU-ICU testing set, the earliest correct alert occurring within the 3-hour window preceding the annotated CRI onset was identified. The time difference between this alert and the CRI onset was defined as the early warning lead time. Lead times were grouped into 5-minute bins, and the frequency in each bin was calculated as the proportion of true positive CRI events whose earliest alert fell within that bin. Distributions are shown for alerts generated by the **EAEWS** and by a **Random Forest** model using three decision thresholds: the default probability threshold (0.5), the threshold maximizing the F1 score and the threshold maximizing the Youden Index. Random forest thresholds were determined using the SYSU-ICU training set. **(B). Model performance under different decision thresholds in testing set**. Performance of EAEWS was compared with that of the Random Forest model under the three corresponding decision thresholds, evaluated on the SYSU-ICU testing set cohort using sensitivity (SE), positive predictive value (PPV), and F1 score**.** Error bars represent 95% confidence intervals (CIs) for the differences. **McNemar test was applied to compare EAEWS with other different decision thresholds. *p < 0.05, ** p < 0.01. (C) Global feature contributions in EAEWS ranked by trigger frequency in SYSU-ICU testing set.** Feature contributions in EAEWS were quantified using trigger frequency, defined as the proportion of times a given feature appeared in satisfied rule conditions across all triggered alerts, relative to the total number of feature occurrences across all 40 features and all triggered alerts. Only features contained in rule conditions that were satisfied at the time of alert generation were counted. **(D) Global feature attribution in the Random Forest model assessed using SHAP values.** Feature contributions in the Random Forest model were evaluated using SHAP-based feature attribution on the SYSU-ICU testing set. All Random Forest models here used the same 1-second–resolution vital-sign data and the same set of 40 predefined vital-sign features (**Methods**).

Supplementary Figure 11. Circulatory- and respiratory-derived feature categories contributing to correct prediction of circulatory and respiratory instability in SYSU-ICU testing set

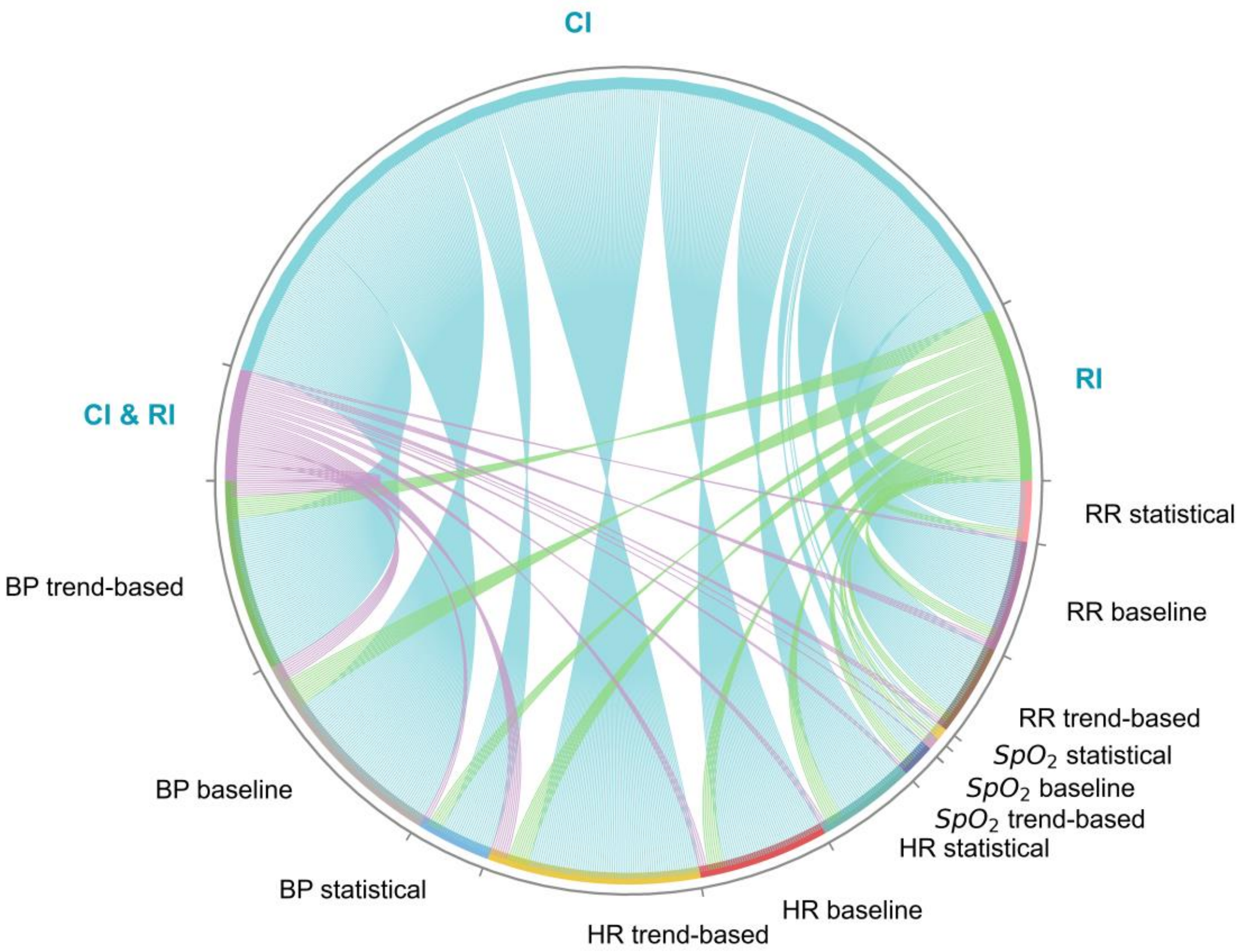

The chord diagram summarizes the feature-threshold conditions that contributed to correct rule activation for circulatory and respiratory instability (CRI) predictions in the SYSU-ICU testing set. The upper semicircle represents correctly predicted event types, stratified as circulatory instability only (CI), respiratory instability only (RI), and combined circulatory and respiratory instability (CI&RI). The lower semicircle shows aggregated feature categories corresponding to the feature-threshold conditions of the decision rules involved in these correct predictions. The original 40 model features were grouped into 12 combined feature categories based on vital-sign modality (HR, BP, RR and SpO_2) and feature group (baseline, trend-based, and statistical). Chords connect event types to feature categories that contributed to correct rule activation. For each event type, the width of each feature-category segment represents the proportion of features belonging to that category among all feature categories appearing in satisfied rule conditions for correctly triggered alerts of that event type.

Supplementary Figure 12. The Sensitivity, PPV, and F1 score of EAEWS using different search windows

A SYSU-ICU testing set

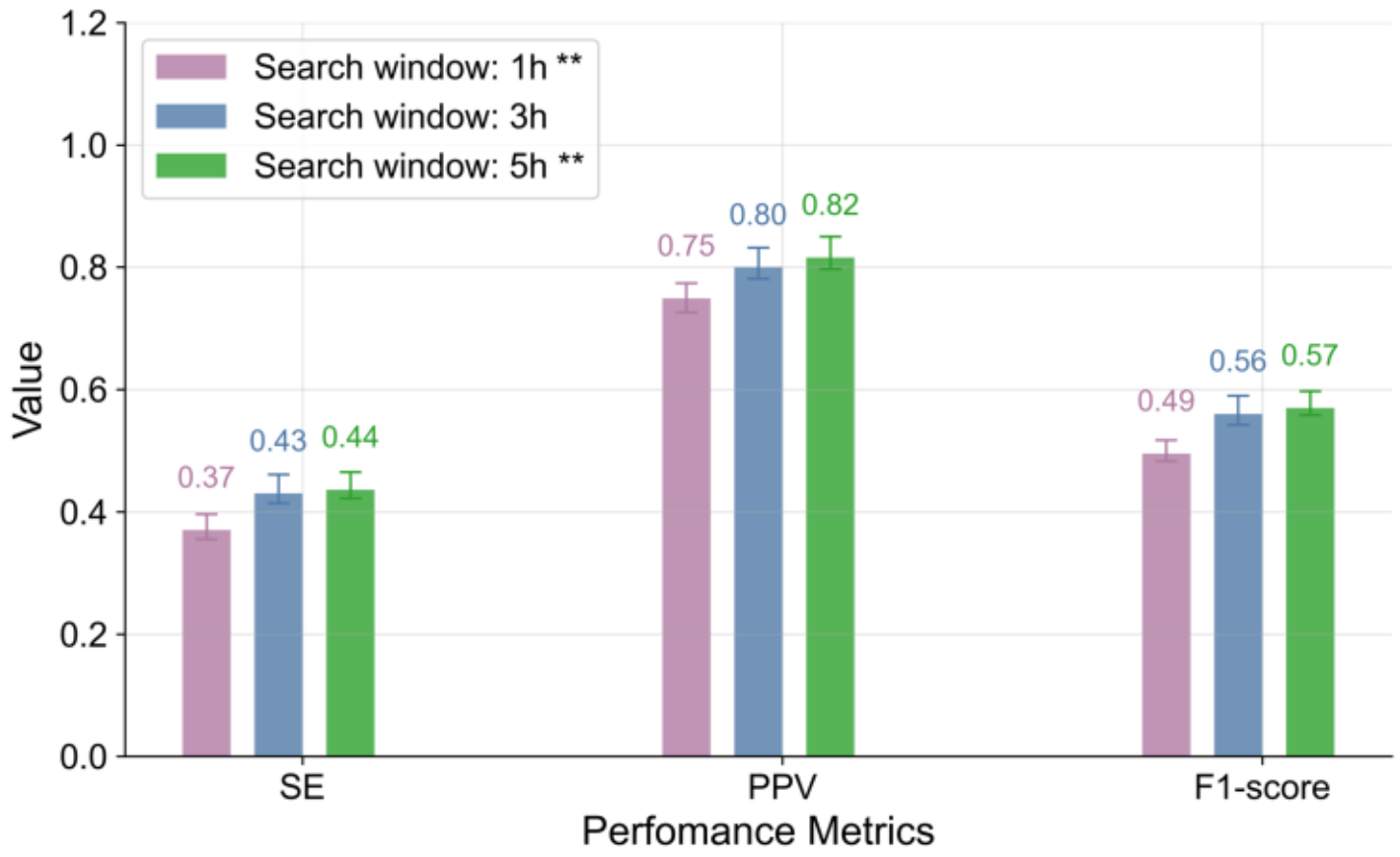

B MIMIC-III

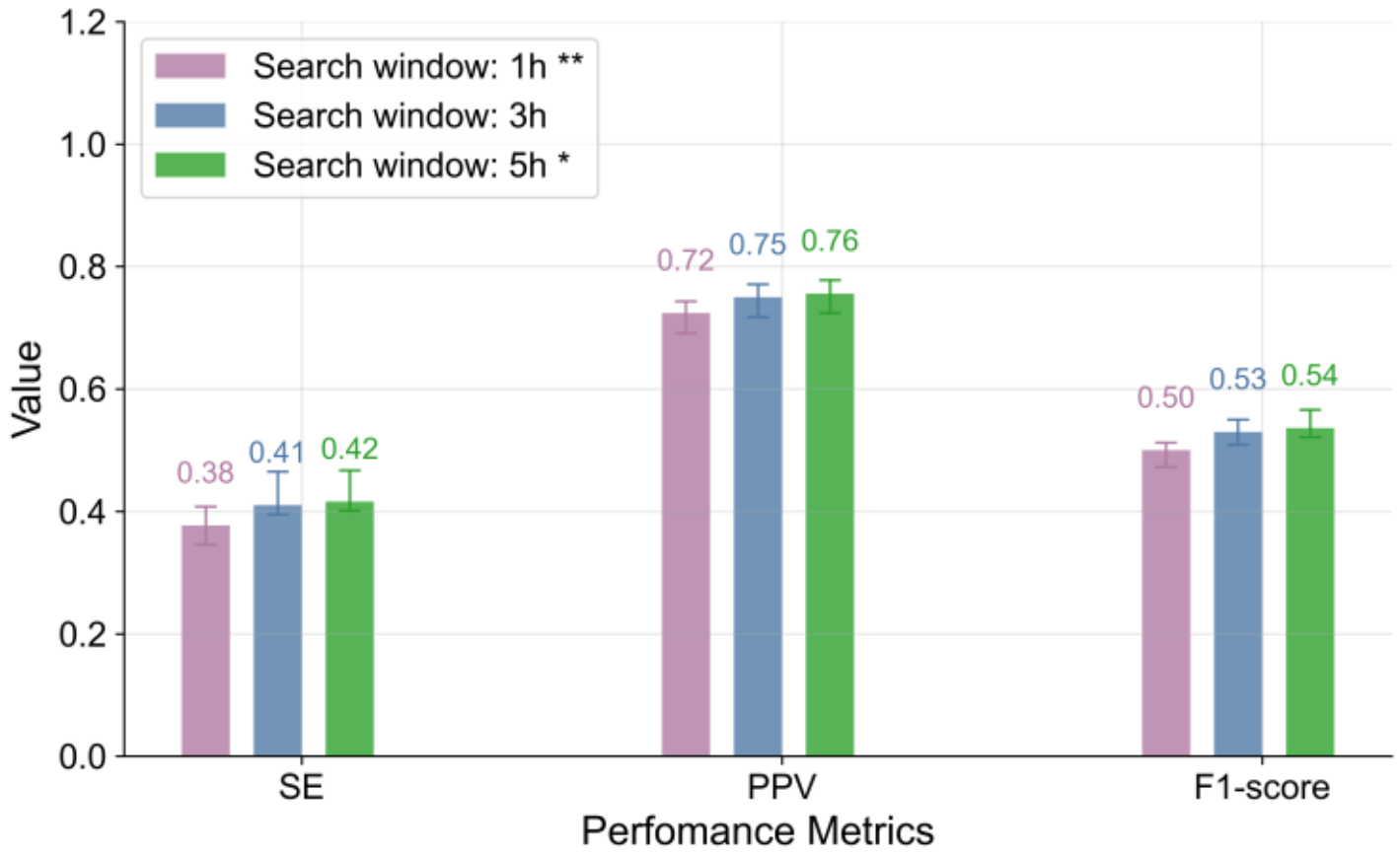

(A) Performance comparison of the EAEWS model under different search windows (1h, 3h, and 5h) in testing set. (B) Performance comparison of the EAEWS model under different search windows (1h, 3h, and 5h) in MIMIC-III. Performance was assessed using sensitivity (SE), positive predictive value (PPV), and F1 score. Error bars represent 95% confidence intervals (CIs) for the differences. **McNemar test was applied to compare search window:3h with other search windows. *p < 0.05, ** p < 0.01.**

Supplementary Figure 13. Comparison of EAEWS and different machine learning models at the default threshold

A SYSU-ICU testing set

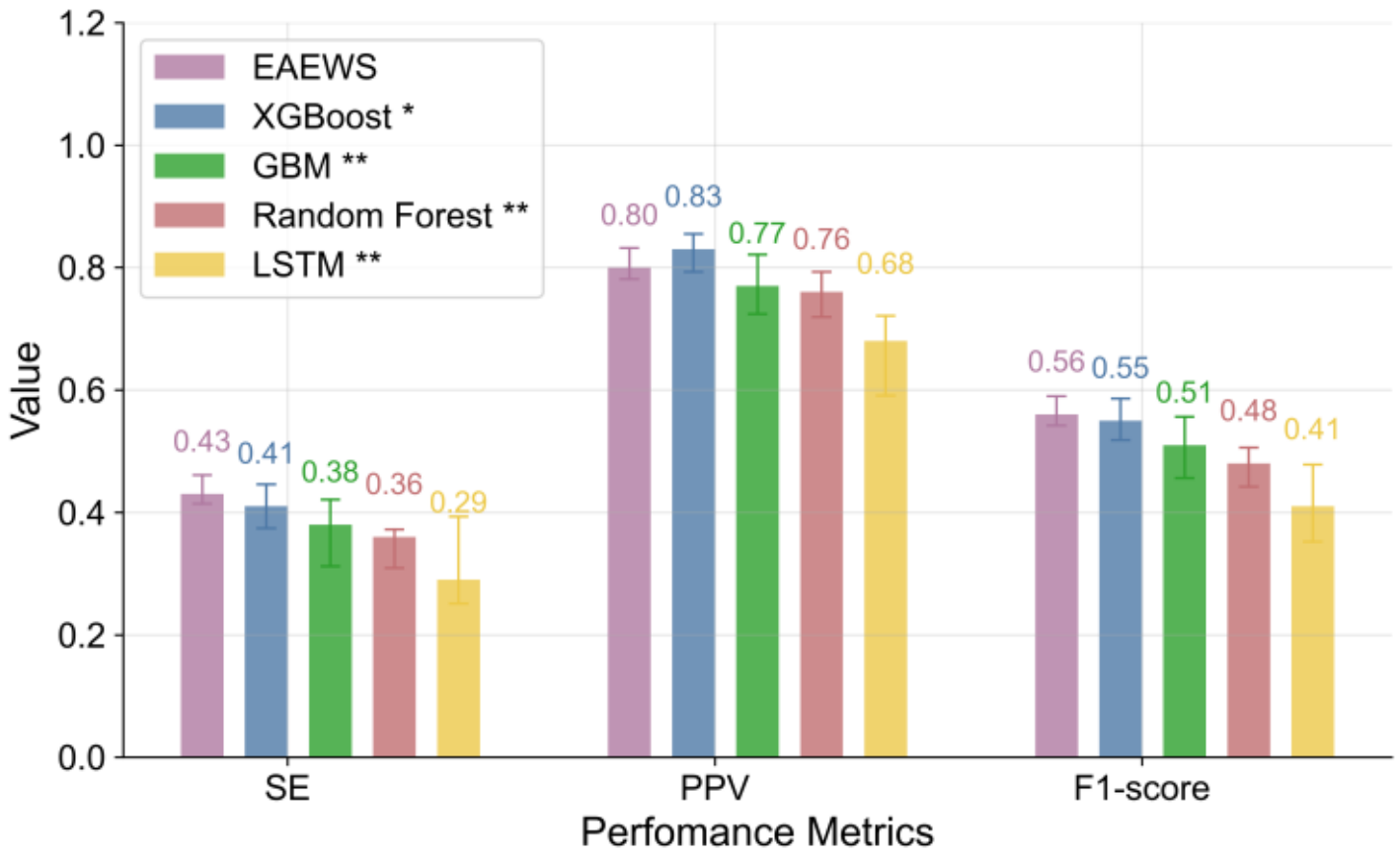

B MIMIC-III

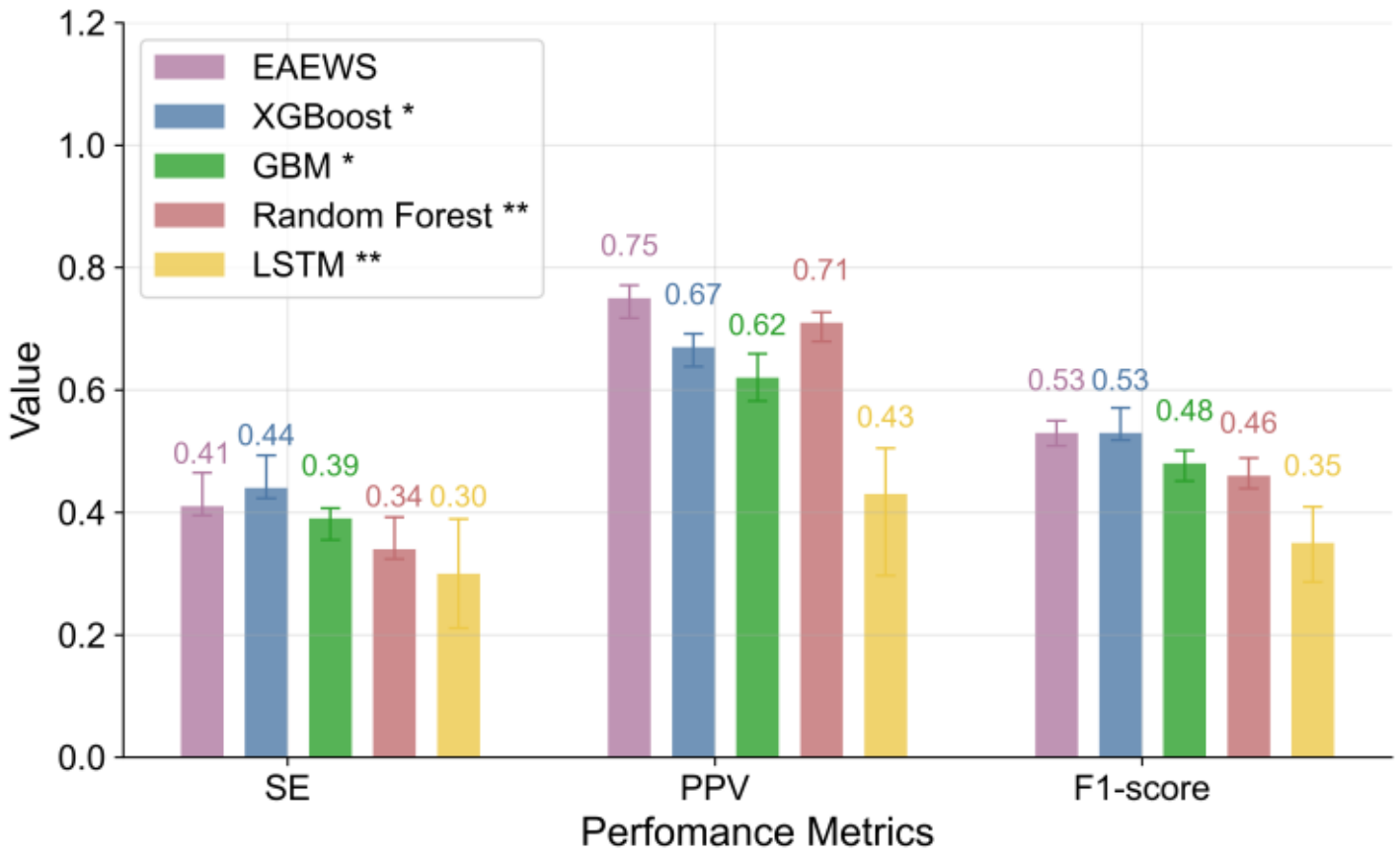

(A) Comparison of model performance using 40 predefined vital-sign variables under default decision thresholds in the SYSU-ICU testing set. (B) Comparison of model performance using 40 predefined vital-sign variables under default decision thresholds in MIMIC-III. Performance of EAEWS was compared with that of the XGBoost, GBM, Random Forest and LSTM model under the default decision thresholds (0.5), evaluated on the SYSU-ICU testing set and MIMIC-III cohort using sensitivity (SE), positive predictive value (PPV), and F1 score. Error bars represent 95% confidence intervals (CIs) for the differences. **McNemar test was applied to compare EAEWS with other machine learning models. *p < 0.05, ** p < 0.01.**

Supplementary Figure 14. Distribution of EAEWS output consistency across lead time

A SYSU-ICU testing set

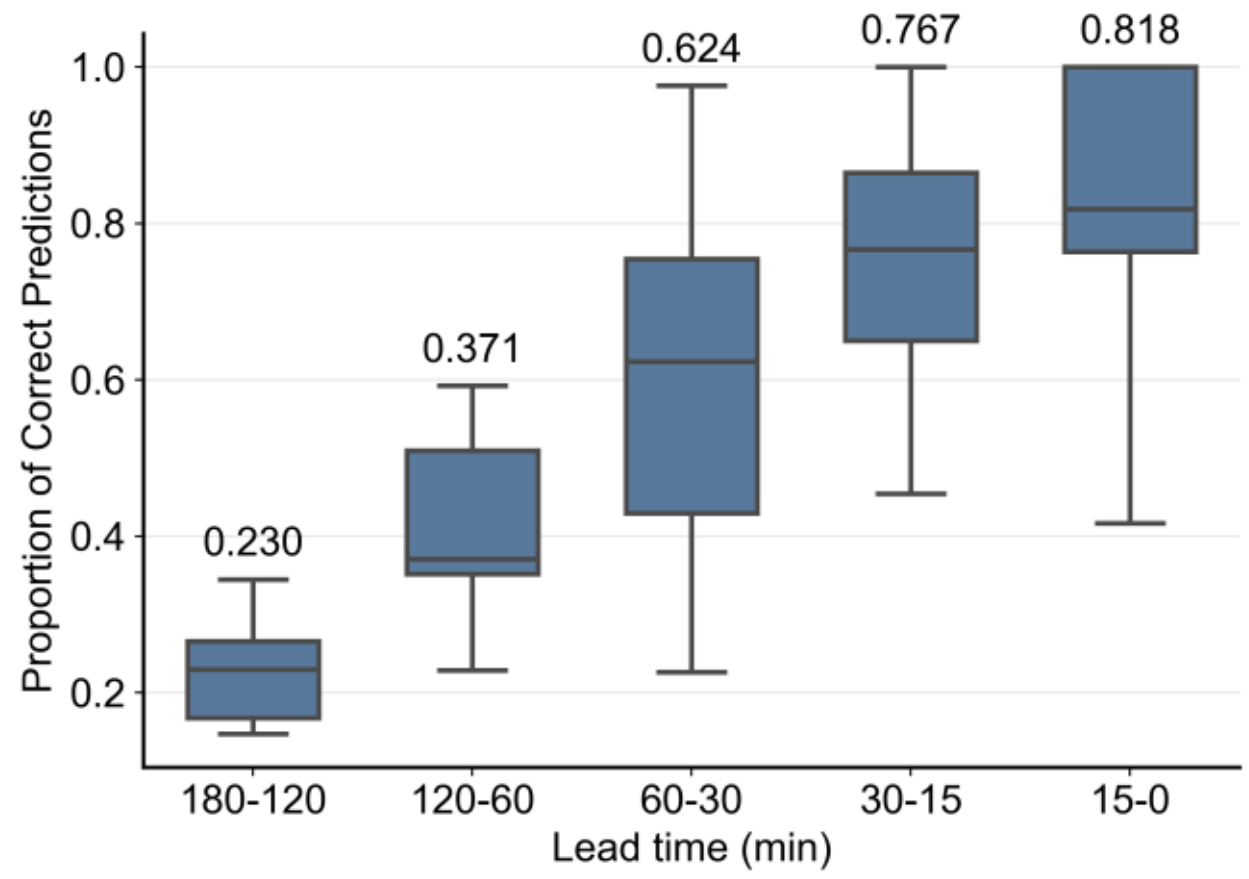

B MIMIC-III

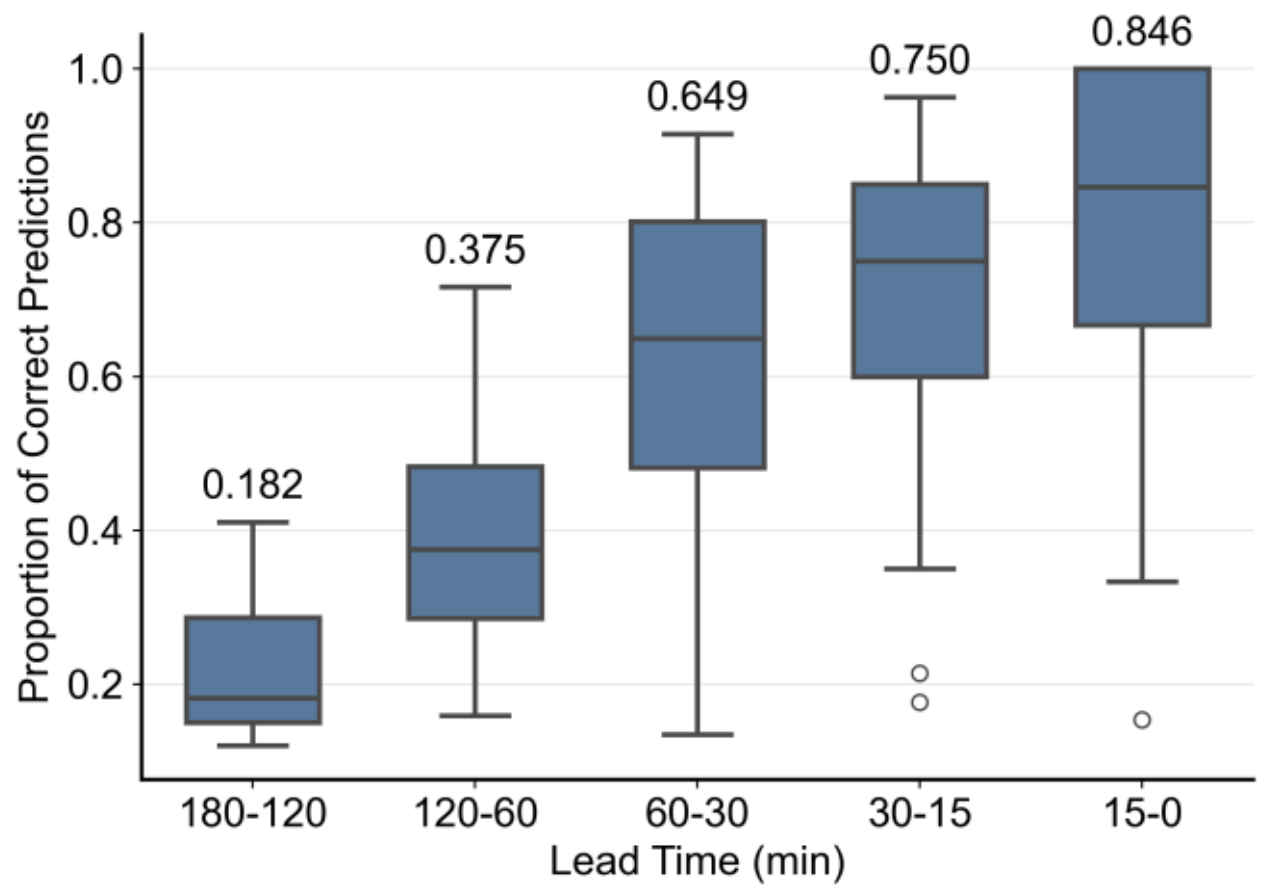

(A) Proportion of correctly predicted events over the 3-hour period before CRI onset in testing set; (B) Proportion of correctly predicted events over the 3-hour period before CRI onset in MIMIC-III. For each correctly predicted event, consistency was defined as the proportion of time during which the model continuously generated positive alerts between the first correct alert and event onset. Specifically, consistency was calculated as: Proportion of correct prediction= mean (number of minutes with correct alerts / total minutes from first correct alert to event onset). Box plots show medians (center lines, value), interquartile ranges (boxes), and ranges within 1.5×IQR.

Supplementary Figure 15. Comparison of AUROC and AUPRC improvement for 1-Second and 1-Minute Inputs Using Single Vital-Sign Features

A SYSU-ICU testing set

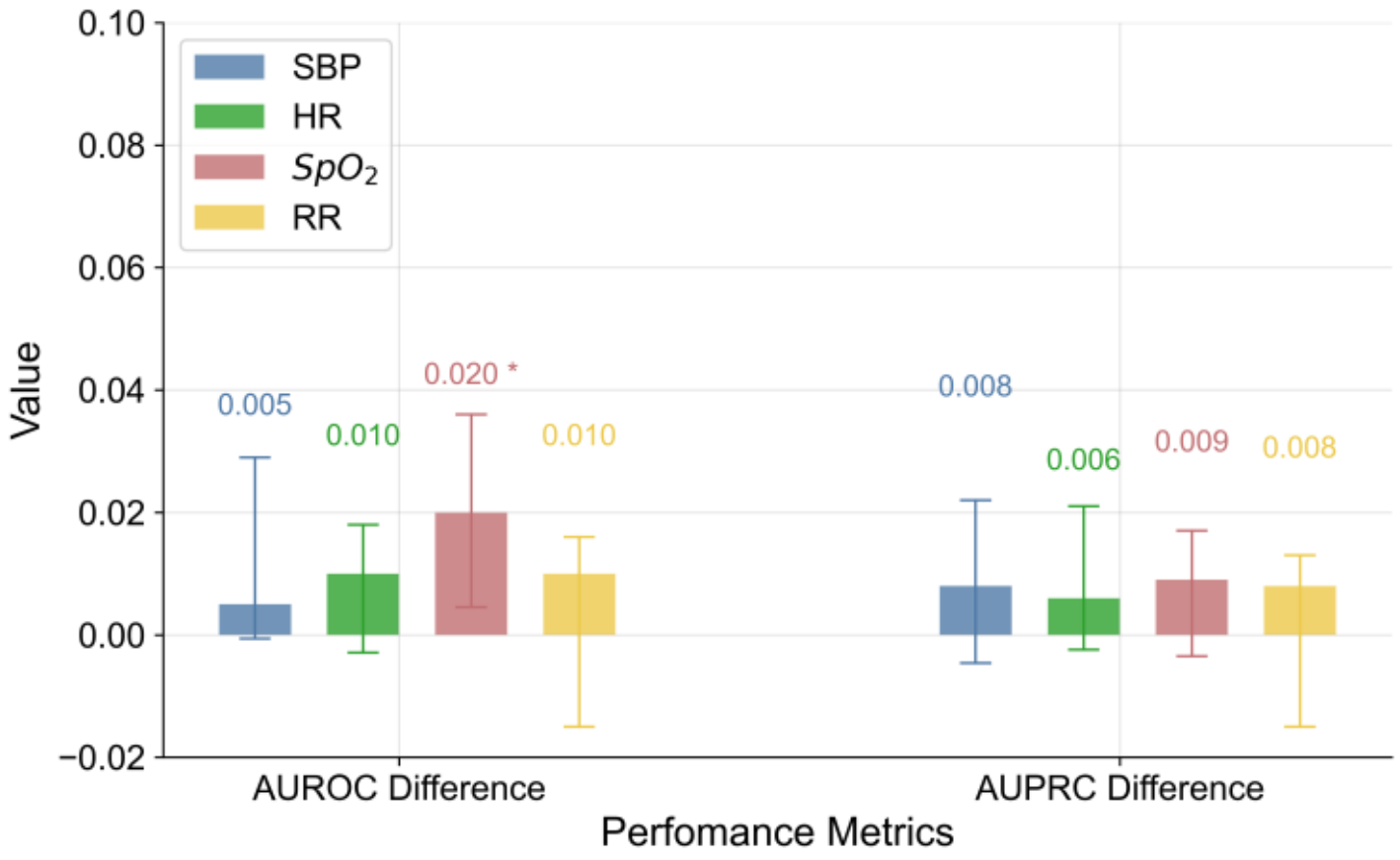

B MIMIC-III

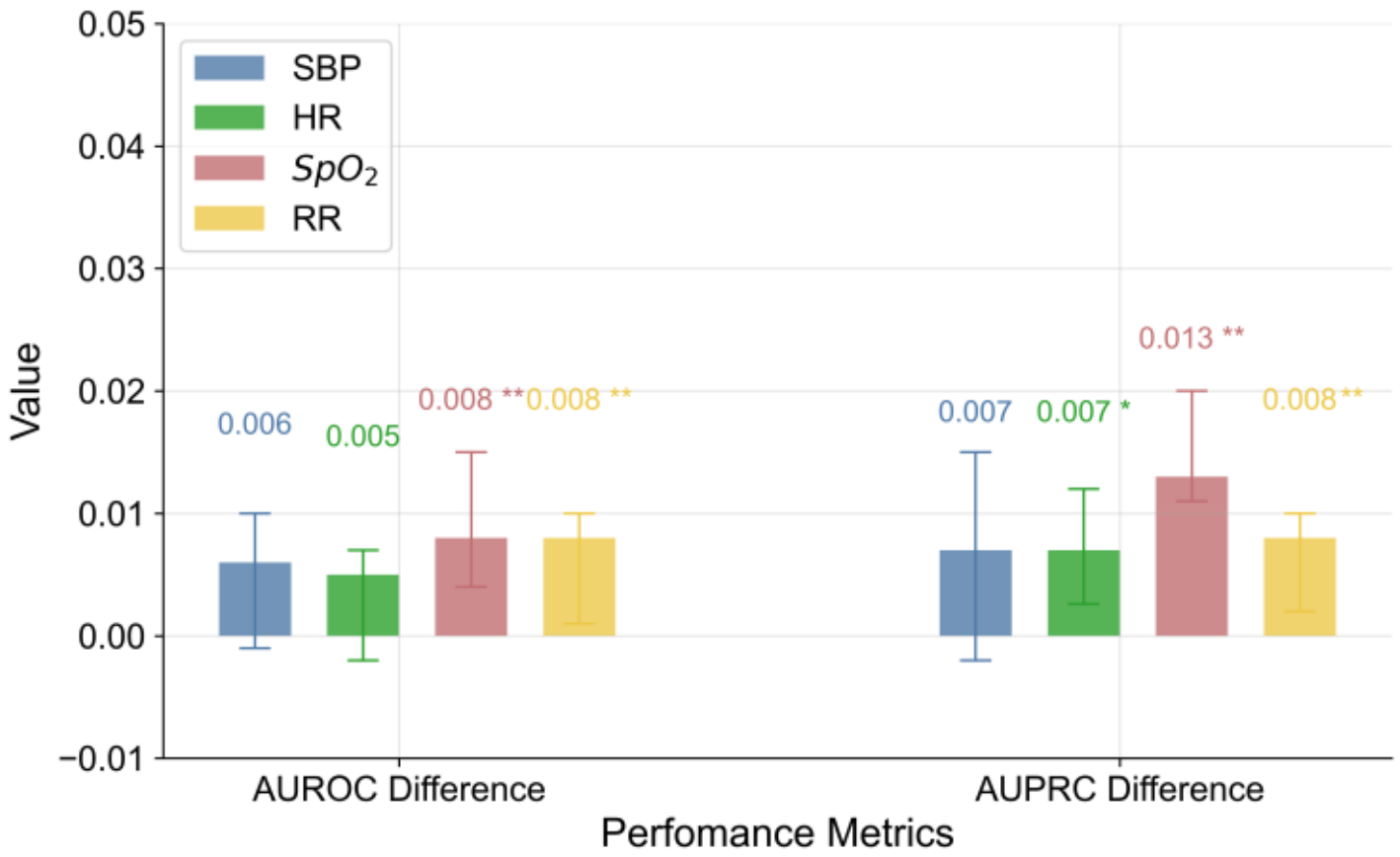

(A) AUROC and AUPRC improvement in random forest models with different vital-sign features from 1-minute to 1-second inputs in SYSU-ICU testing set. (B) AUROC and AUPRC improvement in random forest models with different vital-sign features from 1-minute to 1-second inputs in MIMIC-III. Random forest models were trained on the SYSU-ICU training set using all features derived from each individual vital sign. Error bars represent 95% confidence intervals (CIs) for the differences. *** Denotes the performance differences between models with 1-second and 1-minute resolution under SBP, HR, SpO2, HR, respectively. * p < 0.05, ** p < 0.01.**

Supplementary Figure 16. Sensitivity, PPV, and F1 score comparison between the circulatory instability (CI)-only and full EAEWS

A SYSU-ICU testing set

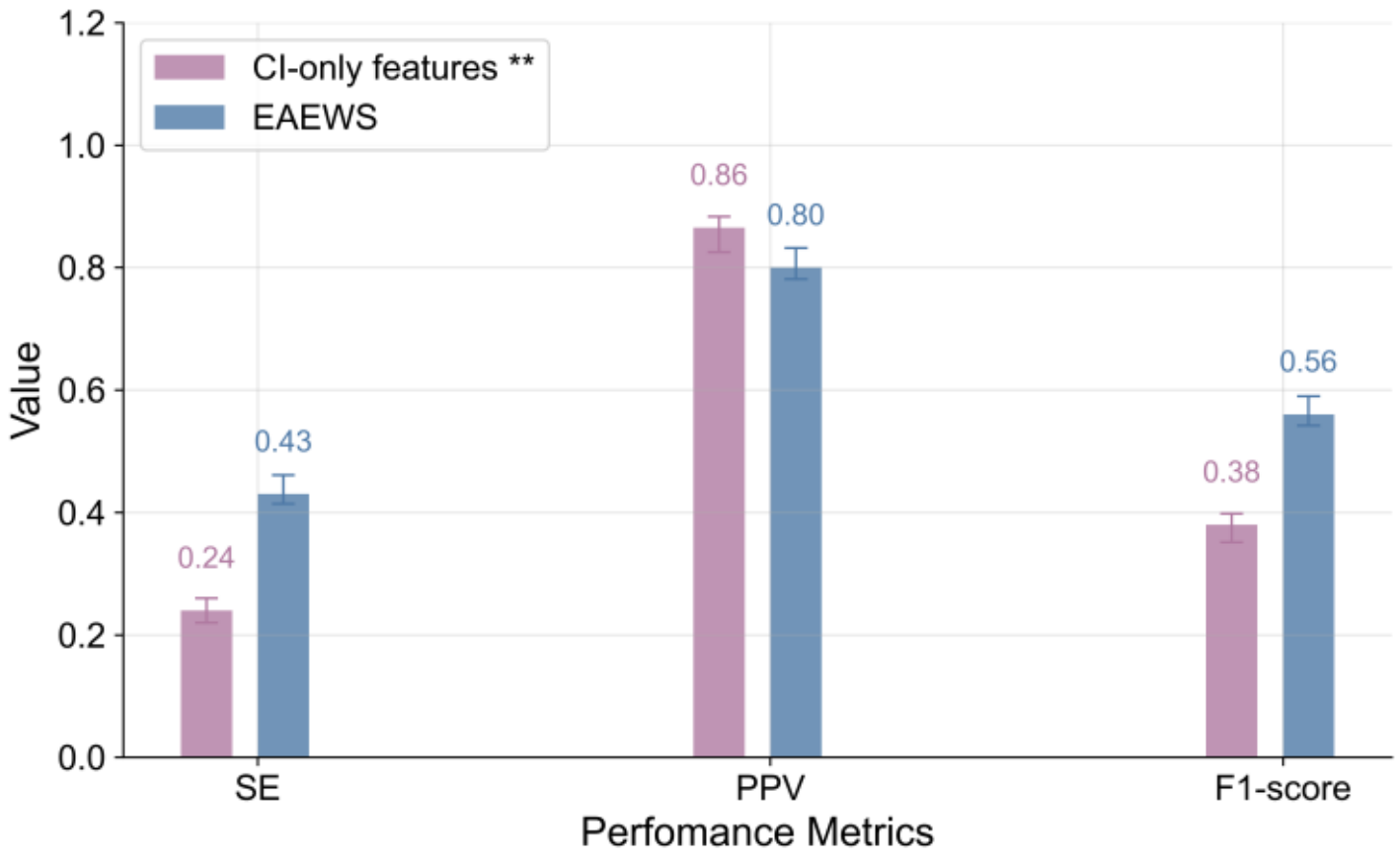

B MIMIC-III

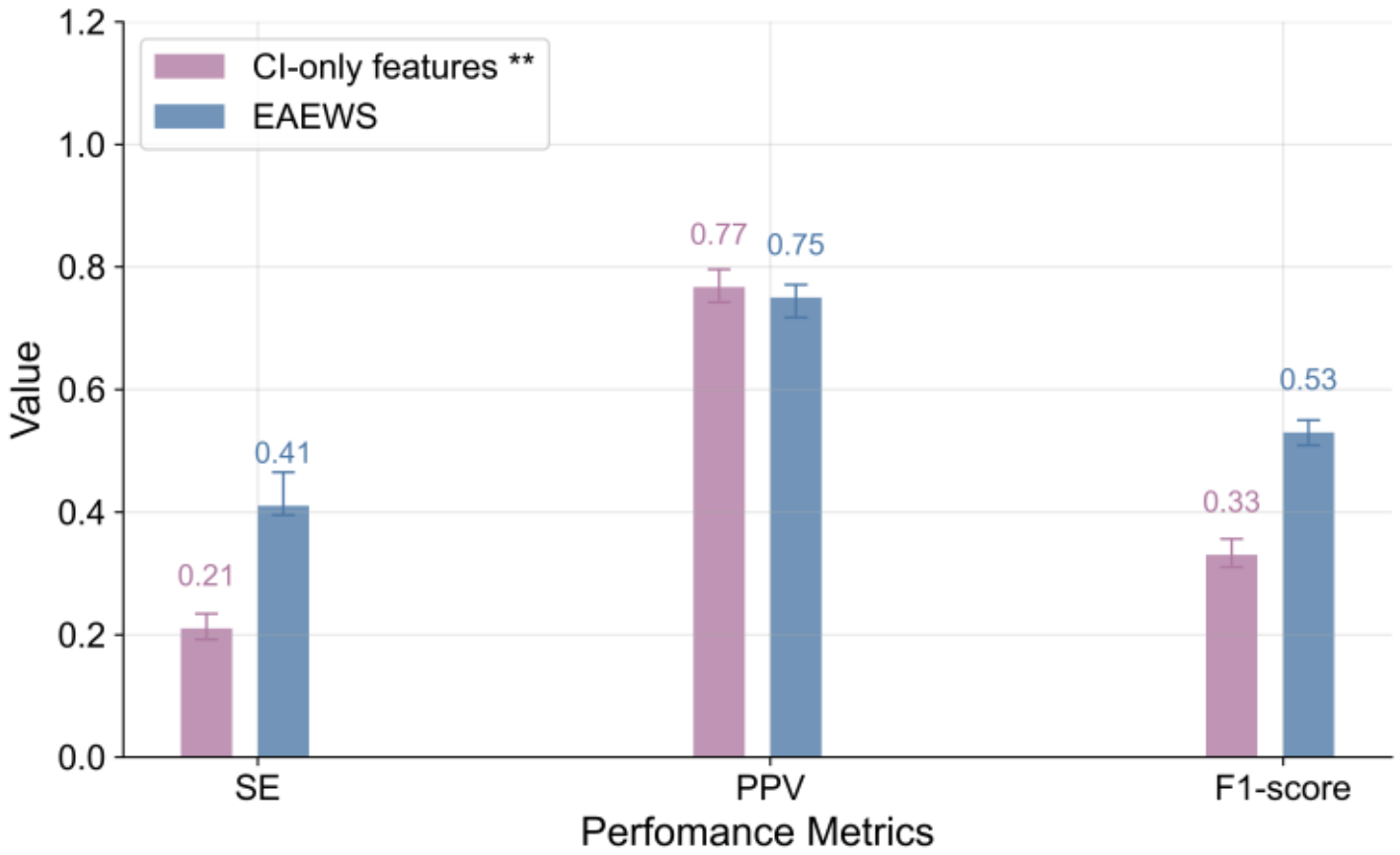

(A) Comparison of the performance of the CI-only rules and the full EAEWS in the testing set. (B) Comparison of the performance of the CI-only rules and the full EAEWS in MIMIC-III. Performance was assessed using sensitivity (SE), positive predictive value (PPV), and F1 score. Error bars represent 95% confidence intervals (CIs) for the differences. **McNemar test was applied to compare EAEWS with CI-only rules. *p < 0.05, ** p < 0.01.**

Mandated checklist: TRIPOD_AI

Section/Topic	Item	Development / evaluation	Checklist item	Reported on page
Title	1	D;E	Identify the study as developing or evaluating the performance of a multivariable prediction model, the target population, and the outcome to be predicted	Title
Abstract	2	D;E	See TRIPOD+AI for Abstracts checklist	Abstract
Background	3a	D;E	Explain the healthcare context (including whether diagnostic or prognostic) and rationale for developing or evaluating the prediction model, including references to existing models	Introduction paras 1-2
	3b	D;E	Describe the target population and the intended purpose of the prediction model in the context of the care pathway, including its intended users (e.g., healthcare professionals, patients, public)	Introduction paras 1-4
	3c	D;E	Describe any known health inequalities between sociodemographic groups	Introduction paras 2–3
Objectives	4	D;E	Specify the study objectives, including whether the study describes the development or validation of a prediction model (or both)	Introduction paras 4
Data	5a	D;E	Describe the sources of data separately for the development and evaluation datasets (e.g., randomised trial, cohort, routine care or registry data), the rationale for using these data, and representativeness of the data	Methods Data and Quality Control paras 2-6<br><br>Results Overview of study design and cohorts paras 1
	5b	D;E	Specify the dates of the collected participant data, including start and end of participant accrual; and, if applicable, end of follow-up	Methods Data and Quality Control paras 1-2, 6
Participants	6a	D;E	Specify key elements of the study setting (e.g., primary care, secondary care, general population) including the number and location of centres	Methods Data and Quality Control paras 1-2, 6
	6b	D;E	Describe the eligibility criteria for study participants	Methods

				Data and Quality Control paras 3
	6c	D;E	Give details of any treatments received, and how they were handled during model development or evaluation, if relevant	Methods<br>Data and Quality Control paras 4–6；Table 2
Data preparation	7	D;E	Describe any data pre-processing and quality checking, including whether this was similar across relevant sociodemographic groups	Methods<br>Data and Quality Control paras 2-6
Outcome	8a	D;E	Clearly define the outcome that is being predicted and the time horizon, including how and when assessed, the rationale for choosing this outcome, and whether the method of outcome assessment is consistent across sociodemographic groups	Methods<br>Data and Quality Control paras 4–6
	8b	D;E	If outcome assessment requires subjective interpretation, describe the qualifications and demographic characteristics of the outcome assessors	Methods<br>Data and Quality Control paras 4–6
	8c	D;E	Report any actions to blind assessment of the outcome to be predicted	-
Predictors	9a	D	Describe the choice of initial predictors (e.g., literature, previous models, all available predictors) and any pre-selection of predictors before model building	Methods<br>Development of Machine Learning Models for CRI Prediction Using High Resolution Vital Signs<br>paras 1-2
	9b	D;E	Clearly define all predictors, including how and when they were measured (and any actions to blind assessment of predictors for the outcome and other predictors)	Methods<br>Development of Machine Learning Models for CRI Prediction Using High Resolution Vital Signs<br>paras 1-2
	9c	D;E	If predictor measurement requires subjective interpretation, describe the qualifications and demographic characteristics of the predictor assessors	Methods<br>Development of Machine Learning Models for CRI Prediction Using

				High Resolution Vital Signs paras 1-2
Sample size	10	D;E	Explain how the study size was arrived at (separately for development and evaluation), and justify that the study size was sufficient to answer the research question. Include details of any sample size calculation	Results Overview of study design and cohorts paras 1–2
Missing data	11	D;E	Describe how missing data were handled. Provide reasons for omitting any data	Methods Data and Quality Control paras 3
Analytical methods	12a	D	Describe how the data were used (e.g., for development and evaluation of model performance) in the analysis, including whether the data were partitioned, considering any sample size requirements	Results Overview of study design and cohorts paras 1-2 Figure 2
	12b	D	Depending on the type of model, describe how predictors were handled in the analyses (functional form, rescaling, transformation, or any standardisation).	Methods Development of Machine Learning Models for CRI Prediction Using High Resolution Vital Signs Development of the Expert-Augmented Early Warning System
	12c	D	Specify the type of model, rationale , all model-building steps, including any hyperparameter tuning, and method for internal validation	Methods Development of Machine Learning Models for CRI Prediction Using High Resolution Vital Signs Development of the Expert-Augmented Early Warning System
	12d	D;E	Describe if and how any heterogeneity in estimates of model parameter values and model performance was handled	Results High-resolution vital-sign dynamics

			and quantified across clusters (e.g., hospitals, countries). See TRIPOD-Cluster for additional considerations	enhance CRI prediction paras 1-3
	12e	D;E	Specify all measures and plots used (and their rationale) to evaluate model performance (e.g., discrimination, calibration, clinical utility) and, if relevant, to compare multiple models	Methods statistical analysis paras 1-2
	12f	E	Describe any model updating (e.g., recalibration) arising from the model evaluation, either overall or for particular sociodemographic groups or settings	-
	12g	E	For model evaluation, describe how the model predictions were calculated (e.g., formula, code, object, application programming interface)	Methods Evaluation Method Methods Statistical analysis paras 1-2
Class imbalance	13	D;E	If class imbalance methods were used, state why and how this was done, and any subsequent methods to recalibrate the model or the model predictions	Methods Statistical analysis paras 1-2
Fairness	14	D;E	Describe any approaches that were used to address model fairness and their rationale	Discussion paras 4
Model output	15	D	Specify the output of the prediction model (e.g., probabilities, classification). Provide details and rationale for any classification and how the thresholds were identified	Figure 7
Training versus evaluation	16	D;E	Identify any differences between the development and evaluation data in healthcare setting, eligibility criteria, outcome, and predictors	Methods Data and Quality Control paras 5-6 Table 1
Ethical approval	17	D;E	Name the institutional research board or ethics committee that approved the study and describe the participant-informed consent or the ethics committee waiver of informed consent	Methods Data and Quality Control paras 1
Funding	18a	D;E	give the source of funding and the role of the funders for the present study	Declaration statements Acknowledgements

Conflicts of interest	18b	D;E	Declare any conflicts of interest and financial disclosures for all authors	Declaration statements<br>Competing Interests
Protocol	18c	D;E	Indicate where the study protocol can be accessed or state that a protocol was not prepared	-
Registration	18d	D;E	Provide registration information for the study, including register name and registration number, or state that the study was not registered	This study was not registered
Data sharing	18e	D;E	Provide details of the availability of the study data	Declaration statements<br>Data Availability
Code sharing	18f	D;E	Provide details of the availability of the analytical code	Declaration statements<br>Code Availability
patient & Public Involvement	19	D;E	Provide details of any patient and public involvement during the design, conduct, reporting, interpretation, or dissemination of the study or state no involvement.	-
Participants	20a	D;E	Describe the flow of participants through the study, including the number of participants with and without the outcome and, if applicable, a summary of the follow-up time. A diagram may be helpful	Methods<br>Data and Quality Control paras 5-6<br>Figure 2
	20b	D;E	Report the characteristics overall and, where applicable, for each data source or setting, including the key dates, key predictors (including demographics), treatments received, sample size, number of outcome events, follow-up time, and amount of missing data. A table may be helpful. Report any differences across key demographic groups	Results<br>Overview of study design and cohorts<br>Table 1
	20c	E	For model evaluation, show a comparison with the development data of the distribution of important predictors (demographics, predictors, and outcome).	Results<br>High-resolution vital-sign dynamics enhance CRI prediction

Model development	21	D;E	Specify the number of participants and outcome events in each analysis (e.g., for model development, hyperparameter tuning, model evaluation)	Result High-resolution vital-sign dynamics enhance CRI prediction; Expert-augmented rule-based modelling improves CRI prediction
Model specification	22	D	Provide details of the full prediction model (e.g., formula, code, object, application programming interface) to allow predictions in new individuals and to enable third-party evaluation and implementation, including any restrictions to access or re-use (e.g., freely available, proprietary)	Methods Development of the Expert-Augmented Early Warning System
Model performance	23a	D;E	Report model performance estimates with confidence intervals, including for any key subgroups (e.g.,	Result High-resolution vital-sign dynamics enhance CRI prediction; Expert-augmented rule-based modelling improves CRI prediction
	23b	D;E	If examined, report results of any heterogeneity in model performance across clusters. See TRIPOD Cluster for additional details	Result Complementary circulatory and respiratory contributions to CRI prediction
Model updating	24	E	Report the results from any model updating, including the updated model and subsequent performance	-
Interpretation	25	D;E	Give an overall interpretation of the main results, including issues of fairness in the context of the objectives and previous studies	Discussion paras 1-4
Limitations	26	D;E	Discuss any limitations of the study (such as a non-representative sample, sample size, overfitting, missing data)	Discussion paras 4

			and their effects on any biases, statistical uncertainty, and generalizability	
Usability of the model in the context of current care	27a	D	Describe how poor quality or unavailable input data (e.g., predictor values) should be assessed and handled when implementing the prediction model	Discussion paras 1
	27b	D	Specify whether users will be required to interact in the handling of the input data or use of the model, and what level of expertise is required of users	-
	27c	D;E	Discuss any next steps for future research, with a specific view to applicability and generalizability of the model	Discussion paras 5
